# Caregiver Reports of Interactions between Children up to 6 Years and Their Family Dog—Implications for Dog Bite Prevention

**DOI:** 10.3389/fvets.2017.00130

**Published:** 2017-08-30

**Authors:** Christine Arhant, Andrea Martina Beetz, Josef Troxler

**Affiliations:** ^1^Department for Farm Animals and Veterinary Public Health, Institute of Animal Husbandry and Animal Welfare, University of Veterinary Medicine, Vienna, Austria; ^2^Department for Special Education, University of Rostock, Rostock, Germany

**Keywords:** child–dog interaction, supervision, parents, dog bite, injury prevention, child safety

## Abstract

In children up to 6 years, interactions such as interfering with the dog’s resources and also benign behaviors (e.g., petting) commonly precede a bite incident with the family dog. Therefore, the aim of the present study was to explore the development of everyday interactions between children up to 6 years and their family dogs and whether parents’ attitudes to supervision are related to those interactions. Additionally, we investigated whether behavior of dogs that had lived in the family for longer than the child differed from those that grew up with children. A self-selected sample of caregivers living with a child up to 6 years and a family dog was surveyed *via* an online questionnaire (*N* = 402). Frequency of observed child behaviors directed toward the dog and dog behaviors directed toward the child were scored on a six-point scale (1—never and 6—very often). Data on characteristics of the caregiver, the child, and the dog were collected, and a section surveying attitudes to supervision of child–dog interactions was included. Additionally, we asked whether the dog already injured the child. Benign child behaviors toward dogs were most frequently reported (mean ± SD: 4.1 ± 1.2), increased with child age (*r*_s_ = 0.38, *p* < 0.001), and reached high levels from 6 months on. Overall, resource-related interactions were relatively infrequent (2.1 ± 1.1). Most common was the dog allowing the child to take objects from its mouth (4.1 ± 1.7). This behavior was more common with older children (*r*_s_ = 0.37, *p* < 0.001). Reported injuries during resource-related interactions occurred while feeding treats or taking objects from the dog during fetch play. Dogs that had lived in the family for longer than the child showed less affiliative behaviors toward the child (e.g., energetic affiliative: *U* = −7.171, *p* < 0.001) and more fear-related behaviors (*U* = −3.581, *p* < 0.001). Finally, the caregivers’ attitudes to supervision were related to all child behaviors (e.g., allow unsafe behaviors—benign child behavior: *r*_s_ = 0.47, *p* < 0.001). The results of this study underline the need for a dog bite prevention approach directed toward the caregivers very early in the child–dog relationship, taking into account the child’s age and individual needs of the dog.

## Introduction

Dogs are one of the favorite animals of preschool children (1). Many children are attracted to dogs, see them as their friends and especially like to cuddle with dogs (2). Children attribute to dogs sentience almost comparable to human beings and especially children who have pet dogs attribute high sentience to them (3). Growing up with a dog can have developmental benefits for children [for review see Ref. (4, 5)]. However, dogs are also the species that causes most injuries in humans (6–8). Although there are no global statistics of dog bite incidents, the WHO estimates that dog bites account for tens of millions of injuries annually and children are most at risk of being bitten (9). Dog bites to children are a significant public health problem (10, 11) and include some serious injuries (12, 13). The overall prevalence of dog bites in children in a telephone survey was 22 per 1,000 children per year and about 40% of those bites were minor, needing no medical care (14). A rise of 63% in dog-related injuries presenting to hospitals between 1998 and 2008 in the UK causes increasing concern (15). In younger children, most dog bites occur at home; often the bite is located on the face, head or neck and is inflicted by a familiar dog (14, 16–18). These incidents are most often preceded by a child-initiated interaction with the dog (16, 19) and one study found that parents were often present (17). These results show that having a family dog with young children poses a risk to the child and even parents might not be able to prevent a bite. Our own research about intervention of parents in child–dog interactions showed that in more than half of the cases, parents do not intervene in a potentially risky interaction with the family dog, whereas they would do so with an unfamiliar dog (20). Parents seem to trust their dog not to act aggressively with their child independent of the context of the interaction. Furthermore, even adults have problems understanding dog body language (21, 22), and dog owners were actually found to be *less* likely than non-owners to recognize dog behaviors indicating fear during an observed child–dog interaction (23).

There is only a very limited number of studies on child–family dog interactions: observations of 2- to 5-year-old children interacting with their family dog lasting about 20 min showed that the initiative came mostly from the child and that the interactions were of short duration compared to interactions with humans (24). In contrast to interactions with other children, the child more often sought body contact to the dog by touching the dog with the hand, petting, or hugging the dog. The tactile behaviors of children toward the dog were less diversified than those of adults. In response to tactile behaviors, the dogs commonly did not react or they approached the child, approached body parts of the child with their muzzle, or retreated from the child. Similarly in a study comparing interactions with a robot dog and a live dog, social touch was the most commonly observed child behavior with the live dog (2). Other common child behaviors were to give an object to the dog or retreat from the dog (24). Clearly threatening or painful child behaviors toward the dog were also observed. The child behavior that led to most attempts to bite was pulling on the dog’s tail, hair, or paw but in general, manifestly aggressive dog behaviors were seldom observed (24). However, more subtle dog behaviors that might indicate that a dog does not feel comfortable in an interaction such as ear and tail movements, body position, yawning, nose licking, or blinking (25) were not coded. The most commonly observed dog behaviors were to sniff the child, to take an object the child presented to the dog or to retreat from the child (24). Observations of child–family dog interactions have also revealed that the types of behaviors observed were related to the age of the child: children aged 2–3 years displayed more agonistic/aversive behaviors toward the dog, children aged 3–4 years more appeasing and linking behavior, and children aged 4–5 years more object-related interactions (24). Another possibly relevant link of child age with dog age was that children were often bitten by dogs that were older than the child (19).

Most dog bites by familiar dogs are preceded by a child–dog interaction (17, 19). Tactile child behaviors toward the dog that are intended to be friendly are also referred to as benign behaviors and can be precursors of a dog bite (16, 17). This type of interaction was found to be associated with an increased risk of a face or head bite (17). Other child–dog interactions preceding dog bites in children younger than 6 years were object- or resource-related interactions, disturbing the resting dog, painful interactions and other interactions that are aversive for the dog (16, 17). Although child–dog interactions are an essential factor contributing to the risk of being bitten, no studies about child–dog interactions in children younger than 2 years are available. We also have a shortage of knowledge about child–family dog interactions occurring during everyday life, how they develop depending on age of the child, and how they relate to the parent’s attitudes to supervision.

The aims of our exploratory study were to survey the occurrence of everyday child–dog interactions in children up to 6 years living with a family dog; to investigate how interactions with the dog develop depending on the age of the child; to explore the relationships of child–dog interactions with caregiver attitudes to supervision; and to investigate whether being accustomed to living with children impacts on the dogs behavior toward the child.

## Materials and Methods

### Questionnaire

To explore the daily lives of parents and other caregivers living with a child up to 6 years and a family dog, a questionnaire with a total of 160 questions in German was developed based on literature review, dog bite prevention programs, experiences of dog owners and experts working in dog bite prevention. Additionally, to identify relevant child–dog interactions, 35 YouTube videos showing child–dog interactions were viewed. The search terms were child dog, child plays with dog, child dog funny, kids and dogs, and 4-year-old plays dog. Selection criteria were that the child should be in the study’s age range and that only one child interacted with a single dog. A maximum of 3 min were screened for interactive behaviors (mean length of the videos: 141 s; range: 26–495). Based on these videos, on informal discussions with dog owners living with small children in their home and relevant literature [e.g., (16, 17, 24, 26)], a list of possible interactions was generated. It included child behaviors directed toward the dog and dog behaviors directed toward the child. The questions were kept as short and as simple as possible and did not distinguish between situations where children initiated an interaction on their own or situations where caregivers encouraged an interaction. Sample questions are “My child pulls on body parts of the dog, e.g., tail, ears”; “My child pets the dog on the head”; “My dog jumps up on the child”; and “My dog barks at the child.” These questions regarding the observed frequency of child–dog interactions were scored on six-point scales with the extremes labeled “Never” (score = 1) and “Very often” (score = 6). Further sections relevant to this work are characteristics of the participant, the child, and the dog. Child age was collected with the following categories: 0.25, 0.5, 0.75, 1, 1.5, 2, 2.5, 3, 3.5, 4, 4.5, 5, 5.5, and 6 years. Dog characteristics included the question whether the focal dog had already lived in the family (and grown up without children) before the focal child or a sibling was born. Additionally, we asked whether the caregiver had ever considered finding a new home for the dog because living with child and dog was too challenging, and whether this dog had already injured this child and in which context. The latter question was an open-ended question. Results on caregiver attitudes to supervision and daily management of child and dog are presented elsewhere (20). However, for two of the supervision attitudes subscales identified *via* principal component analyses—“attentiveness,” “allow unsafe behaviors”—relationships with child and dog interactive behavior and child age are explored in the present article. The items of these subscales were scored on a six-point scale ranging from “do not agree at all” (score = 1) to “totally agree” (score = 6). The “attentiveness” subscale represents the mean of six items such as “I always have an eye on the child and dog if they are in the same room.” The “allow unsafe behaviors” represents the mean of six items such as “as long as the child is nice to the dog, they can play or cuddle with the dog as much as they want” [for details see Ref. (20)]. A draft version of the questionnaire was tested with persons from the target group. Test persons needed about 30 min to complete the revised final questionnaire.

### Survey

The survey was carried out as an online survey *via* “Survey Monkey.” It was open from July 21 to November 26, 2014. The questionnaire was advertised *via* facebook (e.g., facebook page of the Vetmeduni Vienna), newsletters (e.g., Royal Canin Austria), and a German dog magazine. Participants had to own a dog (“family dog”) and be living with a child 6 years old or younger. If respondents had multiple children or dogs, they were asked to choose a focus child and dog, namely the child and dog that they observed to have the most interactions.

### Data Analyses

All statistical analyses were carried out with IBM SPSS Statistics 20 or 22 (SCR_002865). For the descriptive presentation of the frequency of observed child–dog interactions in the text of the results section scores 5 and 6 were grouped and termed “frequently.”

As principal component analyses did not result in easily interpretable subscales, the items concerning child interactive behaviors were grouped according to the grouping of antecedents of dog bites in children from Reisner et al. (16) (Table 1). We added a scale for dog care activities but left grooming within the original classification of Reisner in the aversive non-painful activities scale. Dog interactive behaviors were grouped according to functional or emotional similarities in behavior and potential risk for the child (Table 2). The scores of the “scales” were obtained by calculating the mean of the items in each of the scales.

**Table 1:** Caregiver reports of child behaviors toward the family dog and Spearman rank correlations with child age (*N* ranges between 347 and 365).

	Mean	SD	Min	Perc. 25	Median	Perc. 75	Max	Child age *r*_s_
**Child—benign**	4.14	1.18	1.00	3.43	4.29	5.14	6.00	0.38***
Speak to dog	4.48	1.66	1.00	3.00	5.00	6.00	6.00	0.51***
Pet dog on body	5.05	1.26	1.00	5.00	6.00	6.00	6.00	0.42***
Pet dog on head	4.74	1.46	1.00	4.00	5.00	6.00	6.00	0.43***
Hug dog	3.78	1.94	1.00	2.00	4.00	6.00	6.00	0.47***
Kiss dog	3.07	1.82	1.00	1.00	3.00	5.00	6.00	0.29***
Reach for dog	3.95	1.71	1.00	2.00	4.00	5.00	6.00	−0.23***
Approach or follow dog	4.05	1.70	1.00	3.00	4.00	6.00	6.00	0.03^ns^
**Child—resting**	2.27	1.10	1.00	1.33	2.00	3.00	6.00	0.19***
Wake sleeping dog	1.97	1.25	1.00	1.00	2.00	2.00	6.00	0.17**
Lay down near to resting dog	2.73	1.76	1.00	1.00	2.00	4.00	6.00	0.29***
Leave resting dog alone^a^	4.88	1.38	1.00	4.00	5.00	6.00	6.00	0.05^ns^
**Child—resources**	2.07	1.06	1.00	1.00	1.75	2.75	6.00	0.27***
Attempt to take away dog food or bowl	1.56	1.21	1.00	1.00	1.00	1.00	6.00	−0.03^ns^
Attempt to pet feeding dog	1.73	1.26	1.00	1.00	1.00	2.00	6.00	0.09^ns^
Take child toys from dog	2.92	1.89	1.00	1.00	3.00	5.00	6.00	0.37***
Attempt to take dog toys/chews from dog	2.07	1.48	1.00	1.00	1.00	3.00	6.00	0.12*
**Child—aversive non-painful**	2.10	0.80	1.00	1.57	2.00	2.57	5.29	0.45***
Restraint by collar	2.76	1.68	1.00	1.00	2.00	4.00	6.00	0.20***
Grooming	2.33	1.60	1.00	1.00	2.00	4.00	6.00	0.52***
Child yells or screams during interaction	3.29	1.67	1.00	2.00	3.00	5.00	6.00	0.00^ns^
Verbal scolding	2.05	1.24	1.00	1.00	2.00	3.00	6.00	0.45***
Dress dog	1.21	0.70	1.00	1.00	1.00	1.00	5.00	0.25***
Involve dog in child play, e.g., doctor game	1.79	1.30	1.00	1.00	1.00	2.00	6.00	0.37***
Lift dog	1.35	0.95	1.00	1.00	1.00	1.00	6.00	0.30***
**Child—aversive painful**	1.86	0.77	1.00	1.20	1.70	2.40	4.60	−0.01^ns^
Sit, lie or ride on dog	2.15	1.60	1.00	1.00	1.00	3.00	6.00	0.08^ns^
Pull on body parts of dog, e.g., tail, ears	2.34	1.55	1.00	1.00	2.00	3.00	6.00	−0.18***
Inflict pain accidentally, e.g., stepping on	2.08	1.00	1.00	1.00	2.00	3.00	6.00	0.06^ns^
Inflict pain deliberately, e.g., hitting	1.40	0.75	1.00	1.00	1.00	2.00	5.00	0.20***
Throw objects on dog	1.37	0.77	1.00	1.00	1.00	2.00	6.00	0.00^ns^
**Child—dog care**	3.27	1.44	1.00	2.00	3.33	4.33	6.00	0.59***
Feed dog	3.64	1.77	1.00	2.00	4.00	5.00	6.00	0.25***
Lead dog on leash	2.62	1.71	1.00	1.00	2.00	4.00	6.00	0.53***
Request obedience from dog/give commands	3.51	1.87	1.00	1.00	4.00	5.00	6.00	0.65***

*^a^Has been reversed scored for inclusion in scale “child—resting.”*

^ns^p > 0.05; ***p ≤ 0.001; **p ≤ 0.01; *p ≤ 0.05.

**Table 2:** Caregiver reports of dog behaviors toward the child and Spearman rank correlations with child age (*N* ranges between 338 and 352).

	Mean	SD	Min	Perc. 25	Median	Perc. 75	Max	Child age *r*_s_
**Dog leaves alone/ignores child**	4.24	1.65	1.00	3.00	5.00	6.00	6.00	0.05^ns^
Dog—affiliative calm	4.21	1.26	1.00	3.33	4.33	5.33	6.00	0.06^ns^
Sniffs child	4.65	1.36	1.00	4.00	5.00	6.00	6.00	−0.05^ns^
Lick hand or feet	4.08	1.75	1.00	2.00	4.00	6.00	6.00	−0.10^ns^
Lies down with body contact to child	3.91	1.72	1.00	3.00	4.00	5.00	6.00	0.23***
**Dog—affiliative energetic**	2.44	0.91	1.00	1.83	2.33	3.00	5.67	0.33***
Runs toward child	4.17	1.57	1.00	3.00	4.00	6.00	6.00	0.21***
Runs after child	3.55	1.80	1.00	2.00	4.00	5.00	6.00	0.34***
Gentle mouthing	2.01	1.46	1.00	1.00	1.00	3.00	6.00	0.18**
Sits or lies on child	1.48	1.04	1.00	1.00	1.00	1.00	6.00	0.14*
Jumps up	1.74	1.38	1.00	1.00	1.00	2.00	6.00	0.25***
Knocks child over	1.73	1.04	1.00	1.00	1.00	2.00	6.00	0.09^ns^
**Dog—resources**	2.13	0.74	1.00	1.60	2.00	2.60	5.60	−0.27***
Takes food away from child	2.34	1.54	1.00	1.00	2.00	3.00	6.00	0.01^ns^
Takes child toys from environment	2.00	1.33	1.00	1.00	1.00	3.00	6.00	−0.06^ns^
Takes child toys away from child	1.44	0.93	1.00	1.00	1.00	1.00	6.00	0.01^ns^
Allows child to take things from dog mouth^a^	4.08	1.73	1.00	3.00	5.00	6.00	6.00	0.37***
**Dog—fear**	1.92	1.04	1.00	1.00	1.50	2.50	6.00	−0.15**
Withdraw from child	2.32	1.48	1.00	1.00	2.00	3.00	6.00	−0.17**
Startled by child	1.52	0.88	1.00	1.00	1.00	2.00	6.00	−0.05^ns^
**Dog—aggression**	1.17	0.40	1.00	1.00	1.00	1.20	3.60	0.08^ns^
Barks at child	1.33	0.75	1.00	1.00	1.00	1.00	6.00	0.11*
Growls during frontal approach	1.15	0.62	1.00	1.00	1.00	1.00	6.00	−0.08^ns^
Growls during passing by	1.08	0.38	1.00	1.00	1.00	1.00	5.00	−0.07^ns^
Growls with resources	1.18	0.70	1.00	1.00	1.00	1.00	6.00	−0.04^ns^
Snaps at child	1.11	0.43	1.00	1.00	1.00	1.00	5.00	0.04^ns^

*^a^Has been reversed scored for inclusion in scale “Dog—resources.”*

^ns^p > 0.05; ***p ≤ 0.001; **p ≤ 0.01; *p ≤ 0.05.

To assess relationships with child age as collected in the questionnaire, Spearman rank correlations were calculated for the averaged scales and the individual items of child and dog interactive behaviors. Additionally, child age was categorized (up to 6 months, 6–12 months, 1.5–2 years, 2.5–3 years, 3.5–4 years, 4.5–5 years, and 5.5–6 years) and Kruskal–Wallis tests were used to assess differences in child–dog interactions related to child age. The use of the two different tests was considered suitable to also identify non-monotonous relationships between child age and interactive behaviors.

Relationships between child and dog interactive behaviors and the two subscales assessing attitudes to supervision in caregivers were analyzed using Spearman rank correlations. Finally, we explored whether being accustomed to live with children before the focal child was born, differently effects on dog interactive behavior using a Mann–Whitney *U*-test. Due to the explorative nature of this work, we did not correct for multiple testing and only interpret significant correlations ≥0.2.

## Results

### The Participants

Most of the respondents (*N* = 402) were mothers (82.4%) followed by grandmothers (7.1%), fathers (5.3%), other women (3.4%), grandfathers (1.5%), and one other man. The mean age of the participants was 33 ± 9 years (mean ± SD). A high proportion of participants had an academic degree (47%). Two persons 15 years and older (“adult”) lived in 82.5% of the households (one adult: 4.4%, three or more adults: 13%). In 61% of the households, there was one child, two children were present in 32%, and three or more in 7% of the participating households. Of the children chosen as focus child, 53% were girls and 47% were boys and their mean age was 2.5 ± 1.7 years. The households were situated in rural (55.6%), provincial (22.4%), and metropolitan (22%) areas with half of the participants living in Austria, 46.3% in Germany, and the remaining participants in other European countries.

### The Dogs

Of the dogs chosen as focus-dogs, 56% were females (67% spayed) and 44% were males (54% neutered). The mean age of the dogs was 5.5 ± 3.3 years and their mean weight was 23 ± 13 kg. The most common breeds were mixed breeds (26%) followed by Labrador Retriever (9.4%), Golden Retriever (4%), Australian Shepherd (4%), Rhodesian Ridgeback (3%), Jack Russell Terrier (3%), and 80 other breeds. A large majority of the dogs had lived in the household before the child was born (70.4%). Only one respondent admitted that she often thought about finding a new home for the dog because living with child and dog was very difficult. Another 3% thought about this possibility sometimes and 9% rarely. The majority of the participants (87%; *N* = 325) had never considered rehoming the dog.

### Child Behaviors Directed toward the Dog

**Figure 1:**
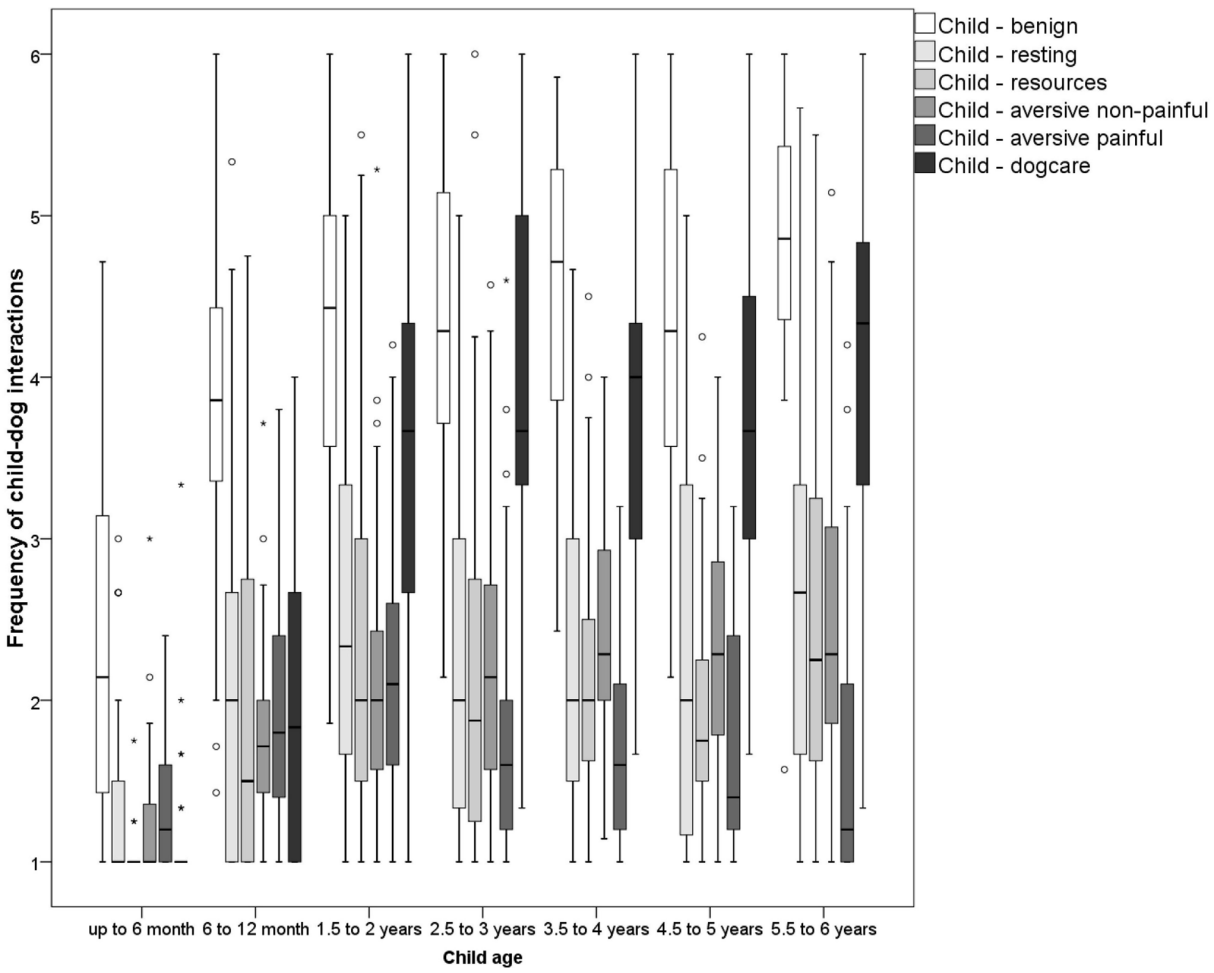
Frequency of reported child behaviors directed toward the dogs grouped by age of child (*N* ranges between 347 and 365).

The commonest observed interactions between children and dogs can be assigned to the category benign behaviors (Table 1). Petting dogs on the body (scores 5 and 6: 75%) and on the head (67%), speaking to the dog (60%) and approaching or following the dog (49%) were frequently observed by caregivers. Child behaviors considered as more problematic from a dog bite prevention point of view, such as hugging (scores 5 and 6: 46%) and kissing the dog (27%) were somewhat less frequent and 21 or 32% of the caregivers, respectively, never observed them (score 1). All these behaviors, except approaching or following the dog, were positively correlated with age of the child (Table 1). A Kruskal–Wallis test showed a significant effect of child age categories on benign child behaviors and graphical inspections revealed an increase in frequency in particular in the first 2 years of life (Chi^2^ = 70.41, *p* < 0.001; Figure 1). Only reaching for the dog showed a small negative correlation with age of the child (Table 1). Overall, 46% frequently observed their child reaching for the dog.

Child behaviors toward a resting dog or a dog interacting with resources were observed rarely by most respondents (Table 1). 49% of the caregivers never observed the child waking the dog, while 7% observed this frequently, and 39% never observed the child lying down near to/beside the resting dog, though 21% observed it frequently. Similarly, 71% reported that the child leaves the resting dog alone frequently (never: 2%). A small positive correlation with child age was present for lying down near to the resting dog (Table 1) and overall children at the age of 1.5–2 years and 5.5–6 years interfered more often with the resting dog (Chi^2^ = 35.31, *p* < 0.001; Figure 1).

Interfering with the dog’s food or food bowl and attempting to pet the feeding dog were rare, being never observed by 76 and 65% of respondents, whereas 6% of caregivers observed these behaviors frequently. Taking child toys from the dog was more common, observed frequently in 27% of the children, but 39% never observed the child retrieving its toys from the dog. About half of the participants (53%) reported that the child never took dog toys or chews from the dog and 10% observed this behavior frequently. The child taking its own toys back from the dog was seen more frequently in older children (Table 1). The other resource-related interactions were at most marginally related to child age. Children were observed interfering with dog resources from the second half of their first year of life onward, with an increase until the second year of life (Chi^2^ = 55.10, *p* < 0.001; Figure 1).

The most commonly reported child behavior classified to be aversive for the dogs was the child yelling or screaming during an interaction (frequently: 28%, never: 20%). Very rare behaviors were dressing the dog (frequently: 1%, never: 89%), involving the dog in child play (frequently: 6%, never: 66%), and lifting the dog (frequently: 3%, never: 84%). Attempts to lift the dog were mostly reported for children 4 years and older (Supplementary Material). Intermediate numbers of respondents reported children restraining the dogs by the collar (frequently: 21%, never: 33%), grooming (frequently: 12%, never: 49%), and verbal scolding (frequently: 5%, never: 46%). All these behaviors, except yelling or screaming during an interaction, correlated positively with child age (Table 1). Weak associations were found for restraining the dog by the collar, dressing the dog, and lifting the dog. A more pronounced increase with age was found for grooming, verbal scolding, and involvement of the dog in child play. Overall, aversive interactions increased until the age of 3.5–4 years and then their occurrence seems to remain stable until the age of 6 years (Chi^2^ = 80.83, *p* < 0.001; Figure 1).

Child–dog interactions with a high risk of inflicting pain on the dog were rarely observed child behaviors. Least commonly reported was throwing objects at the dog (frequently: 2%, never: 75%) and deliberately inflicting pain, e.g., by hitting or kicking the dog (frequently: 1%, never: 71%). More frequently observed were to pull on body parts of the dog such as the tail or ears (frequently: 15%, never: 42%), to sit, lie, or ride on the dog (frequently: 14%, never: 55%), or to inflict pain accidentally by stepping on the dog (frequently: 1%, never: 31%). Painful interactions were barely correlated with age—only deliberately inflicting pain correlated somewhat with child age (Table 1). This child behavior is most prominent in children between 1.5 and 5 years (Supplementary Material). Overall, a significant effect of age was found (Chi^2^ = 29.68, *p* < 0.001; Figure 1): graphical inspection revealed that painful interactions rise in frequency until the age of 1.5–2 years and decline afterward until the age of 6 years.

Involvement of children in dog care correlated strongly with the age of the child (Table 1). The median peaked with 5.5–6 years and a sharp rise was already found in the second year of life (Chi^2^ = 146.77, *p* < 0.001; Figure 1). The more common behaviors reported were feeding the dog (frequently: 37%, never: 19%) and giving commands to the dog (frequently: 39%, never: 27%). Leading the dog on a leash (frequently: 19%, never: 41%) was less common and barely present in children 1 year and younger. For data on grooming, see aversive non-painful interactions.

### Dog Behaviors Directed toward the Child

**Figure 2:**
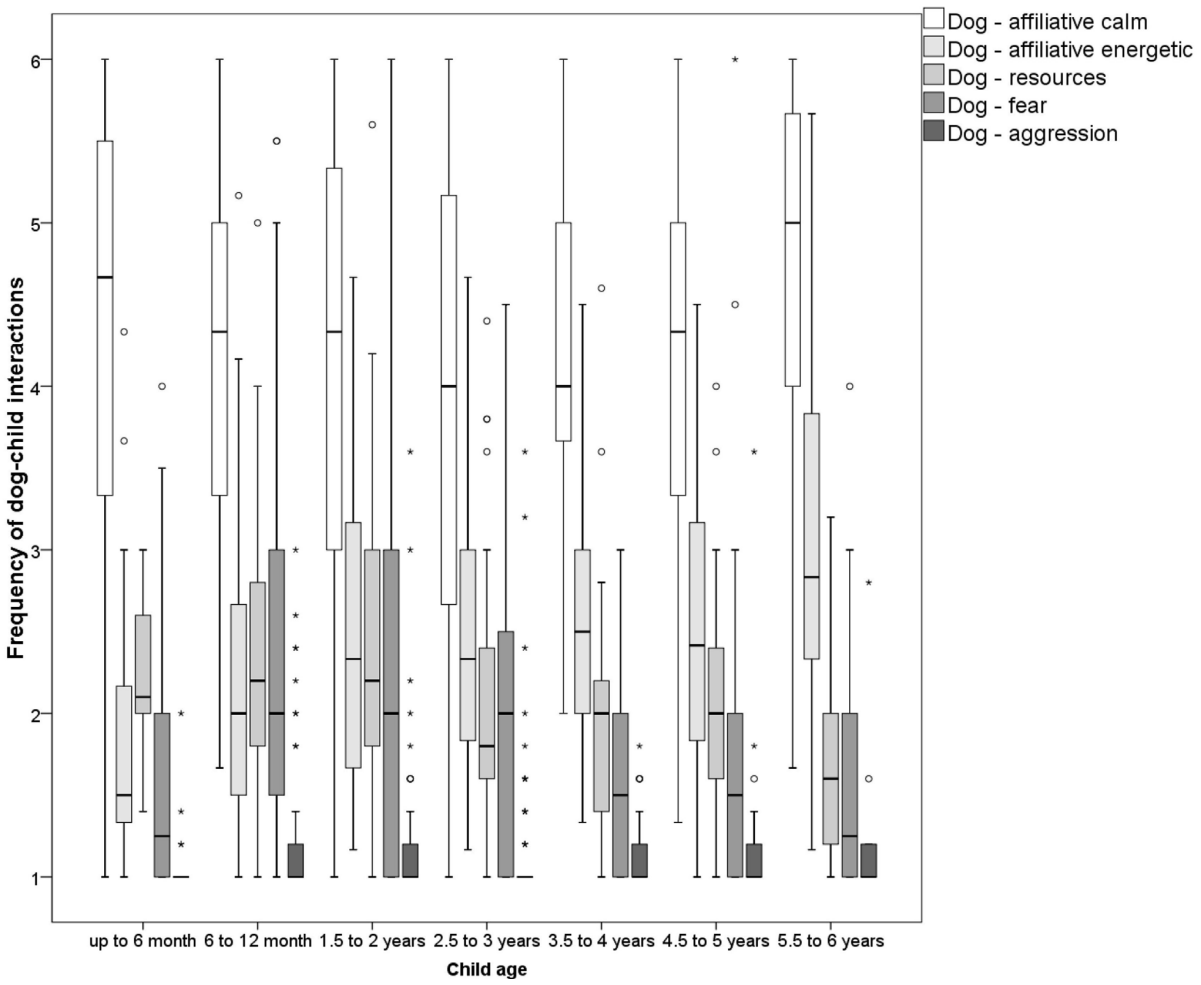
Frequency of reported dog behaviors directed toward the child grouped by age of child (*N* ranges between 338 and 352).

The most common interactions directed by the dog toward the child were calm affiliative behaviors or non-interaction, i.e., ignoring the child (Table 2). Most commonly reported was sniffing the child. Only 2% never observed this behavior and 62% of the participants observed it frequently (scores 5 and 6). A small positive relationship with child age was found for lying with body contact to the child (Table 2) whereas overall there seems to be no relationship of calm affiliative dog behaviors with child age (Chi^2^ = 8.072, *p* = 0.233; Figure 2).

More energetic affiliative behaviors of the dog directed toward the child observed rather frequently were running toward (frequently: 48%, never: 5%) and after the child (frequently: 36%, never: 20%). Running after the child was more often observed with older children (Table 2). Potentially risky dog behaviors such as jumping up (frequently: 8%, never: 69%) or knocking the child over (frequently: 3%, never: 55%) were reported at low levels. Jumping up seemed to increase with child age whereas knocking the child over was not related to child age. Again, intense body contact initiated by the dog such as sitting or lying on the child (frequently: 3%, never: 76%) or even contact with the dogs’ mouth (frequently: 10%, never: 57%) was rarely observed by our participants. Overall, energetic affiliative behaviors were more common with older children (Chi^2^ = 37.540, *p* < 0.001; Figure 2).

Turning to resource-related behaviors, respondents reported on average that dogs sometimes steal food from the child (frequently: 13%, never: 43%). They are less often observed taking child toys from near the child (environment: frequently: 7%, never: 50%) and even more rarely take them directly from the child (frequently: 2%, never: 75%). The most commonly reported behavior in this category was the dog allowing the child to take objects out of the dogs mouth (frequently: 51%, never: 14%). This behavior was more often observed with older children; and this association resulted in an overall correlation of resource-related dog–child interactions with child age (Chi^2^ = 34.249, *p* < 0.001; Figure 2); interfering with the child’s food or toys did not correlate with child age (Table 2).

Fear-related dog behaviors during interactions with the child were not common, but 12% of the dogs frequently withdrew from the child (never: 39%) and 2% frequently exhibited a startle reaction during a child–dog interaction (never: 64%). A small non-monotonous relationship between child age and fear-related dog behavior was found (Chi^2^ = 23.662, *p* = 0.001; Figure 2). Graphical inspection showed a peak of fear-related dog behavior toward children during child age of 6 months to 3 years.

Aggressive behavior toward the child was very rarely observed. The most common behavior that may indicate aggression was barking at the child (frequently: 1%, never: 79%). Although rarely observed, growling at the child occurred more often during a frontal approach of the child (frequently: 1.1%, never: 92%) and in the context of resources (frequently: 1.9%, never: 91%). Growling when the child was passing by was the least often observed context of growling at the child (frequently: 0.5%, never: 95%). Snapping at the child was reported with similar frequencies (frequently: 0.5%, never: 92%). No relationship with child age was found (Table 2; Chi^2^ = 8.156, *p* = 0.227; Figure 2).

#### Injuries Resulting from Child–Dog Interaction

Of the dogs, 53 (16%) had already injured the focus child (*N* = 326). All these injuries were minor and did not need medical attention according to the respondent. Most of the injuries were scratches from the dog’s paws or hematomas when the dog knocked the child over. 11 (3%) instances of biting were reported which resulted in scratches or hematomas: four of these involved disturbing the resting dog, three resulted from a resource-related interaction, two from a painful interaction, and two were reported as occurring during play with the dog as a puppy. None of the reported injuries during resource-related interactions were due to aggression: they were injuries to the child’s fingers and occurred while feeding treats or playing fetch games.

### Relationships between Child and Dog Behavior

All child behavior scales were found to be positively related to the dog behavior scale energetic affiliative dog behavior (Table 3). The strongest relationship was found with benign child–dog interactions (*r*_s_ = 0.58). The weakest relationship of energetic affiliative dog behaviors was found with aversive painful child–dog interactions (*r*_s_ = 0.26). Calm affiliative dog behavior was also related to most of the child behavior scales; only aversive painful interactions and dog care activities resulted in correlations smaller than 0.2. Correlations of child behavior with aggressive dog behavior toward the child resulted in three positive relationships larger than 0.2: these were the child interfering with the resting dog (*r*_s_ = 0.22), aversive painful (*r*_s_ = 0.22), and aversive non-painful child behaviors (*r*_s_ = 0.25) (Table 3).

**Table 3:** Relationships (Spearman rank correlations^a^) between child behaviors directed toward the dog and dog behaviors directed toward the child.

		Child—benign	Child—resting	Child—resources	Child—aversive non-painful	Child—aversive painful	Child—dog care
**Dog—affiliative calm**	*r*_s_	**0.39**	**0.26**	**0.23**	**0.26**	0.15	0.18
	*p*	**<0.001**	**<0.001**	**<0.001**	**<0.001**	0.006	0.001
	*N*	**346**	**359**	**353**	**347**	356	356
**Dog—affiliative energetic**	*r*_s_	**0.58**	**0.44**	**0.44**	**0.54**	**0.27**	**0.43**
	*p*	**<0.001**	**<0.001**	**<0.001**	**<0.001**	**<0.001**	**<0.001**
	*N*	**343**	**354**	**349**	**341**	**351**	**351**
**Dog—resources**	*r*_s_	−0.15	0.02	−0.01	−0.17	0.11	−0.17
	*p*	0.007	0.678	0.811	0.002	0.038	0.001
	*N*	341	355	348	341	352	351
**Dog—fear**	*r*_s_	−0.17	−0.02	0.01	−0.08	0.13	−0.13
	*p*	0.002	0.679	0.794	0.151	0.018	0.014
	*N*	348	361	355	349	359	358
**Dog—aggression**	*r*_s_	0.13	**0.22**	0.18	**0.25**	**0.22**	0.17
	*p*	0.014	**<0.001**	0.001	**<0.001**	**<0.001**	0.001
	*N*	342	**353**	349	**342**	**351**	349

*^a^Results with correlation coefficients ≥0.2 are in bold type*.

### Relationships with Attitudes to Supervision in Caregivers

All reported child behaviors toward the dog were related to the caregivers’ attitudes to supervision (Table 4). Participants who reported being more attentive during supervision overall reported less frequent child–dog interactions. The strongest negative relationships with attentiveness were found for aversive non-painful interactions (*r*_s_ = −0.35) and dog care activities (*r*_s_ = −0.36). Respondents who reported allowing more unsafe behaviors toward the dog reported all child behaviors toward the dog to be more frequent. The strongest relationships were found with the child interfering with the resting dog (*r*_s_ = 0.55) and with benign child–dog interactions (*r*_s_ = 0.47). Least related to this parent supervision subscale were dog care activities (*r*_s_ = 0.23).

**Table 4:** Relationships (Spearman rank correlations^a^) between caregiver attitudes to supervision of child–dog interactions and child and dog interactive behaviors.

		Attentiveness	Allow unsafe behavior
**Child—benign**	*r*_s_	−**0.30**	**0.47**
	*p*	**<0.001**	**<0.001**
	*N*	**322**	**318**
**Child—resting**	*r*_s_	−**0.29**	**0.55**
	*p*	**<0.001**	**<0.001**
	*N*	**335**	**326**
**Child—resources**	*r*_s_	−**0.31**	**0.43**
	*p*	**<0.001**	**<0.001**
	*N*	**330**	**324**
**Child—aversive non-painful**	*r*_s_	−**0.35**	**0.43**
	*p*	**<0.001**	**<0.001**
	*N*	**322**	**318**
**Child—aversive painful**	*r*_s_	−**0.21**	**0.35**
	*p*	**<0.001**	**<0.001**
	*N*	**331**	**325**
**Child—dog care**	*r*_s_	−**0.36**	**0.23**
	*p*	**<0.001**	**<0.001**
	*N*	**330**	**323**
**Dog—affiliative calm**	*r*_s_	−0.10	**0.23**
	*p*	0.080	**<0.001**
	*N*	338	**330**
**Dog—affiliative energetic**	*r*_s_	−**0.27**	**0.39**
	*p*	**<0.001**	**<0.001**
	*N*	**333**	**325**
**Dog—resources**	*r*_s_	0.09	0.01
	*p*	0.106	0.850
	*N*	333	324
**Dog—fear**	*r*_s_	0.19	−0.17
	*p*	<0.001	0.002
	*N*	341	332
**Dog—aggression**	*r*_s_	0.03	0.08
	*p*	0.625	0.143
	*N*	331	323

*^a^Results with correlation coefficients ≥0.2 are in bold type*.

The caregivers’ attitudes to supervision were markedly less related to dog behavior directed toward the child than to child behavior directed toward the dog (Table 4). We found that participants who rated themselves as supervising more attentively reported lower levels of energetic affiliative dog behaviors (*r*_s_ = −0.27), while caregivers who allowed more unsafe behaviors reported more calm (*r*_s_ = 0.23) and energetic affiliative (*r*_s_ = 0.39) dog behaviors toward the child.

#### Child Age and Attitudes to Supervision

Attentiveness during supervision of child–dog interactions decreased with age of the child (*r*_s_ = −0.38, *p* < 0.001, *N* = 325; Chi^2^ = 47.673, *p* < 0.001) whereas allowing unsafe interactions with the dog was not associated with child age (*r*_s_ = 0.09, *p* = 0.102, *N* = 318; Chi^2^ = 11.475, *p* = 0.075).

### Do Dogs That Lived in the Family Earlier Than the Children Behave Differently?

Dogs that had lived with the family before the child or the children were present were reported to display less calm affiliative (*U* = −5.238, *p* < 0.001, *N* = 365; before child: mean ± SD: 4.0 ± 1.3, with child: 4.7 ± 1.1) and energetic affiliative behaviors (*U* = −7.171, *p* < 0.001, *N* = 358; before child: 2.2 ± 0.8, with child: 3.0 ± 1.0) and to show more fear-related behavior toward the child (*U* = −3.581, *p* < 0.001, *N* = 368; before child: 2.1 ± 1.1, with child: 1.6 ± 0.9). No differences were found for aggressive behavior (*U* = −0.216, *p* = 0.829, *N* = 356; before child: 1.2 ± 0.5, with child: 1.1 ± 0.3) and resource-related dog behavior (*U* = −1.853, *p* = 0.064, *N* = 358; before child: 2.2 ± 0.7, with child: 2.0 ± 0.9).

## Discussion

This exploratory study reporting data from a self-selected sample recruited *via* facebook (e.g., Vetmeduni Vienna), newsletters (e.g., Royal Canin Austria), and a German dog magazine shows that a wide range of interactions are already observed in infants of up to 6 months; only interactions related to resources and dog care were almost never present at this age. A steep rise in the frequency of most interactions is seen in the second half of the first year of life. Specific child–family dog interactions such as hugging the dog, grooming the dog or taking objects from the dog’s mouth were found to increase with the child’s age in our sample of children up to 6 years. All the child’s behaviors directed toward the dog were related to the caregivers’ attitudes to supervision. This suggests that parental supervision quite effectively shapes the child’s interactive behavior. Dog interactive behavior seems to depend, besides individual characteristics, on prior experience with children and did show some relationships with child behavior and caregiver supervision. A limitation of this exploratory study might be that respondents admitted leaving their child and dog alone for a moment (20) and this might result in underreporting of interactive behaviors occurring during their absence. In particular, behaviors that are not tolerated by the caregivers might occur more commonly when they are not looking.

Child motor development proceeds quickly and our results show that even in the youngest age group up to 6 months benign interactions are reported. Overall, benign interactions are the most commonly reported child behaviors and they already occur quite frequently in children 1.5 years old. Most of the benign behaviors are more often observed in older children, except reaching for the dog, which was reported less in older children. More than half of the participants in this survey reported allowing the child to interact with the dog as long as she or he is nice to the dog (20) which implies that they do not see benign behaviors as a risk for a bite incident. However, hugging the dog, for example, is a child behavior that is considered to cause discomfort or even fear in many dogs. In a study observing child–dog interactions, about 18% of observed instances of hugging or kissing the dog and about 10% of petting interactions led to the dog retreating (24). Benign behaviors were the third most common type of interactions preceding a dog bite in children 6 years or younger (16) and the risk of a bite to the face was found to be three times higher when a benign interaction preceded a dog bite (17). Benign interactions preceding a bite are seldom initiated by the dog (only 16%) and when the bite incident was preceded by petting, parents were present in about four-fifth of the cases (17). As benign interactions are the most common interactions observed by our respondents, only a minority of them seem to lead to a bite incident. The outcome of a child–dog interaction depends on how the dog perceives the situation. Therefore, a key to avoiding bites during this type of interaction might be the ability to recognize the dog’s emotional state. With young children, parents have to guide any interaction and it is their duty to recognize the dog’s warning signals and intervene in or even prevent a benign (or other) child–dog interaction. However, it has been shown that in particular low-intensity warning signals such as yawning, nose licking, turning or walking away are frequently not recognized even by adults (21). Although experience can improve perception and recognition of fearful dog behaviors (22), in a study asking participants to categorize the emotional state of a dog in a child–dog interaction, non-dog owners actually identified fear-related dog behavior more accurately than dog owners (23). Our own study results showed that most parents trust their family dog in contexts that experts would recommend avoiding (20). Children have even more difficulties in recognizing dog facial expressions and body language (27, 28). Children from about 3 years on can be trained to interpret dog body language (21, 29, 30) but recognition of the low-intensity signals (21) long-term retention of the knowledge (29) were problematic. Therefore, it remains a priority to train parents and dog owners to recognize dog body language and to intervene in or prevent an interaction with the child if a dog signals that it feels uncomfortable.

Although low compliance with the probably best known general recommendation “Never leave the child alone with the dog” was found in this sample (20), more of the respondents seem to be aware of and want to follow the recommendation that a resting dog should not be disturbed: the child interfering with the resting dog was seldom reported by our participants. However, a similar child behavior, lying down near the resting dog (with body contact), was reported with higher frequency, although this has the same effect of disturbing the dog. This could be a result of poor attention to contextual cues of interactions. During this survey, participants were also asked to rate pictures of child–dog interactions and whether they would intervene in these interactions (20). One of them depicted a child sitting in the dog’s basket with the dog. Most participants stated that they would not intervene with their family dog but would do so if the child interacted with an unfamiliar dog. Familiarity of the dog was more important to the respondents than the context of the picture, although interfering with a resting dog is a common precursor of bite incidents (16). One effort to educate parents and 3- to 6-year-old children to be attentive to the context of a child–family dog interaction and how to act is “The Blue Dog” dog bite prevention program (31–33). Being attentive to contextual cues of interactions instead of the child’s intent (e.g., disturb versus lie down near resting dog) seems to be another important aspect of guiding interactions between children and dogs.

Interactions involving objects that might be considered as resources by the dog occur at rather low levels. However, resource-related interactions were found to be the most common interactions preceding a dog bite in children younger than 6 years, so that this type of interaction involves a high risk of injury (16). Our respondents reported the lowest frequencies for the child interfering with dog food or dog food bowls, attempts to pet the feeding dog and attempts to take chew objects or dog toys from the dog. More frequently, children retrieved their own toys from the dog (median: 3) and even more frequently, respondents stated that the dog allowed children to take objects from its mouth (median: 5). The latter two behaviors were more often observed in the older children in our sample. Similarly, during observations of child–dog interactions more object-related interactions were found in children between 4 and 5 years (24). An activity that might account for this higher frequency of resource-related interactions in older children could be playing fetch games with the dog. Repetitive fetch games are considered to cause high arousal in the dog (34) and this might be a factor increasing the risk of a bite incident. Sometimes, it is recommended to play food-related games instead. However, this does not necessarily reduce the risk, as it could lead to resource-guarding aggression by the dog (35). Also, in our sample minor injuries of the child’s fingers occurred during feeding treats or playing fetch games. Therefore, the only resource-related “interactions” that can safely be recommended to parents are indirect interactions. For example children can prepare food stuffed toys or cardboard boxes with treats for the dog and then watch the dog exploring the toy they prepared, safely separated by a baby gate.

In general, aversive interactions, in particular those that might cause pain, were rarely observed by our respondents. The two types, non-painful and painful interactions show different patterns of development in our study sample. The aversive non-painful interactions steadily rise and are most frequent in children from 2.5 years on. In contrast, the aversive painful interactions reported by our respondents were most frequent in children between 6 months and 2 years old and declined thereafter until the age of 6 years. This decline of aversive painful interactions might reflect the development of motor skills and/or empathy on the part of the children [for review see Ref. (36)]. Although infants in their first year of life show emotional arousal in reaction to distress of others humans (37), the cognitive appraisal of pain has been shown to increases in children aged between 3 and 9 years (38). Because young children might inflict pain inadvertently by pulling the dogs’ hair, tail or ears, dog bite prevention programs such as “Dogs and Toddlers” recommend guiding the hand of the child during petting (39). In our sample, this child behavior was most prevalent from 6 months to 1 year. Also, providing dogs with resting places separated (but not isolated) from child play areas, can prevent incidents of inadvertent falls on the dog. Our data confirm that this seems particularly important in children up to 2 years. Consistent with our study, data from an observational study showed that the youngest observed age group, namely children aged between 2 and 3 years showed the highest frequency of aversive behaviors toward dogs—comparable to the frequency of aversive behaviors toward other children (24). Additionally, Millot et al. reported that it seems that young children tend to pass aggression on to the dog. Dogs were more likely to retreat from such encounters than other children and were considered to serve as an outlet for the child’s emotions (24). Although aversive painful interactions were more common in younger children, intentionally inflicting pain was most commonly reported for children between 1.5 and 5 years in our sample. At this age, children show behaviors that are harmful for animals out of “curiosity about and exploration of their natural world” (40). Ascione states that it is very unlikely that these behaviors are intended to be cruel and that they should be seen as opportunities to teach children how to treat animals kindly (40). Another factor that might contribute to these behaviors is that aversive child behaviors elicit the most reactions in the dogs (24). The dogs either reacted by retreating from the child, with appeasing behavior or with aggressive behavior. From the child’s point of view any reaction of the dog might be more rewarding than a non-reaction and this might positively reinforce risky child behavior. However, aversive painful interactions were the second most common type of interactions preceding a dog bite in children up to 6 years (16). An aggressive response due to pain can be very fast and intense (41) and this may leave very little time for an intervention to protect the child. It also causes a stress response in the dog (42). Therefore, the goal should be to prevent all pain-related interactions by management and guided interactions.

In the older children of our sample, higher levels of benign and aversive non-painful interactions, and in particular, increased dog care activities were reported. Correlations of benign and aversive non-painful interactions resulted in the highest relationships with affiliative energetic dog behaviors and these dog behaviors were also observed more often with older children. These results might reflect the emergence of a more complex overall repertoire of interaction. Indeed, parents often report that children of this age group develop more complex relationships with dogs, involving more affectionate attachment and also making more demands on the dog. Correlations with single items support this view, as behaviors like speaking to the dog, hugging the dog, grooming the dog, leading it on a leash as well as behaviors such as requesting obedience or scolding the dog verbally are more often present. As the child grows older, its interest in social play grows (43) and this might lead to inclusion of the dog in role play activities of the child which were observed by our respondents more often in children from 2.5 up to 6 years. Another aspect of playing with the dog—attempting to dress it—was rare overall but observed most often in 5.5- to 6-year-old children. Dog care activities reached high levels by the time the children were 2.5 years old and the dog allowing the child to take objects from its mouth was more prevalent in the 2.5–6 years old. All these behaviors can induce emotions such as fear in dogs and many of them have the potential to inflict pain. Nevertheless we found that parental attention decreases with increasing age of the child. Taken together, in particular the older age groups of our sample might be at risk from interactions that are from the child’s perspective playful, caring or “just necessary” but are potentially aversive or even painful to the dog. Parents should be prepared for this change in child–dog interactions. Beginning from the age of about 2.5 years on, guiding play activities of the child and the dog might be even more challenging as the play becomes more complex. There does not seem to be any justification for reducing attentiveness. Activities that both child and dog enjoy but are not too arousing will be highly individual and care should be taken to provide resting times and to respect the dog’s body language and needs.

Fear-related dog behaviors probably play a very important role for bite incidents with family dogs. We found that parents of children from 6 month to 3 years reported the highest levels of avoidance of the child and being startled by the child in their family dog. At this age, children start to explore their world, first crawling, then by uncoordinated walking, emitting sounds of pleasure or anger that can be quite different from adult human behavior. We found that dogs that experienced the focal child as the first child in the family and that had lived in the family before children arrived more frequently showed fear-related behaviors toward the child. These dogs had also an overall lower level of dog-initiated interactions with the child (calm and energetic affiliative dog behaviors). Study results support our finding that growing up without a child and being fearful or anxious might be a risk for dog bites, as dogs that bit children were often older than the child they bit (19) and more than two thirds of dogs that had bitten a child exhibited anxiety in other contexts such as separation from the owner or thunderstorms (16). Overall, this shows that these dogs might need more time, possibilities to withdraw and proactive supervision to cope with the arrival of the new family member and that caregivers should learn to recognize and respect the emotional state of their dog. The common assumption that, in dogs that are fearful of children, frequent (benign) interactions with the child will lead to habituation, might even be a factor leading to exacerbation of the fear (44). In fact, a fearful dog encountering the stimuli that cause the fear might even sensitize the dog, and sooner or later the growing fear and “forcing” the dog into an interaction with the child might lead to a bite incident that effectively terminates the fear-inducing interaction (45). The two most important principles of behavior modification in fearful dogs are to avoid exposing the animal to the (full intensity) fear-inducing stimuli and to use the techniques of desensitization and counterconditioning to change the emotional state of the animal (44). The consultation of a professional, e.g., a veterinary behaviorist, which always should include a risk assessment and implementation of safety measures (46), can identify the most suitable treatment options for an individual case. One treatment option might also be rehoming of the dog if risk to the child or animal welfare necessitates (44, 47). In particular, if the dog has already displayed aggressive behavior toward family members, immediate measures to assure safety of the people involved have to be taken (10, 35) and the dog owner should be aware that lifelong management may be required (44, 47, 48).

The dogs in our sample generally showed low levels of aggressive behavior toward the child. Many kinds of aggressive behavior such as growling are normal signaling behavior (25) that should be seen as valuable, easy-to-recognize warning signals that should never be punished (49). It is important to note that young children can misinterpret showing teeth as friendly dog behavior (28). However, underlying causes of aggressive behavior such as growling should be addressed with the help of a professional (35, 50). Our study results show that a number of child behaviors could contribute to increased irritability of the dog toward the child, e.g., more frequent disturbance of the resting dog, aversive painful and aversive non-painful interactions. This underlines the need to prevent those child behaviors even if the dog seems to be very tolerant toward the child in general. Also, in our sample a frontal approach by the child was more likely to elicit growling than just passing by. This is in accordance with the recommendation that children should avoid approaching a stationary dog and instead should call the dog and leave it alone if it does not approach (17).

The respondents of our survey stated that minor injuries needing no medical attention were inflicted by about one fifth of the dogs. Scratches by the dog’s paws or hematomas from being knocked over by the dog were more common than injuries caused by the dog’s teeth. About 3% of the child–dog pairs were involved in bite incidents causing minor injuries. This number is similar to the total number of bites in another report (51) although our self-selected sample did not report medically attended dog bite incidents. The low total number of bite incidents (11 in total) does not allow a direct comparison of causes to other studies (16, 17, 52). Most common were incidents of aggression involving a resting dog that could easily have been prevented if the resting place of the dog had been inaccessible to the child. Interestingly the resource-related incidents were all not due to aggressive dog behavior but occurred while feeding treats or playing fetch games with the dog. Also, our respondents did not report incidents during benign child–dog interactions. Overall, these reports of minor injuries support the need for educating parents to use temporal and/or spatial separation of child and dog (e.g., during resting or at times when the dogs is exited, e.g., during greeting) and to teach dogs calm behaviors around children to prevent jumping up or knocking the child over. To avoid injuries by the paws, items used for separation should have openings small enough to prevent the dog reaching through with its paws and footwear can be used to protect the child’s feet from the dog’s claws.

On the one hand, there is evidence that children profit regarding their development from contact with pets and in particular pet dogs (5, 52); on the other hand children interacting with dogs at this young age are at risk of being bitten and the consequences of the bite can be serious, e.g., facial scarring or post-traumatic stress disorders (11, 15, 18, 53). Obviously, there is a trade-off between limiting interactions for safety reasons and the opportunity for developmental benefits from contact with the dog. Therefore ways need to be found to enhance positive effects that at the same time minimize the risk of being bitten. Humans have an affinity to nature and animals (biophilia) (54) and the presence of a calm dog seems to signal a safe environment and was found to promote relaxation (5, 55). Therefore, measures that promote relaxation in the dog such as a safely separated but not isolated resting place, structured positive interactions, and fulfillment of other needs are likely to have relaxing effects on the child and probably the whole family.

Benign behaviors such as petting a dog, in particular if a familiar animal is involved, can activate the oxytocin system (56). Even visual contact with the dog can lead to an increase of oxytocin in the dog’s owner, facilitate affiliative behavior toward the dogs, and in turn increase oxytocin in the dog (57, 58). Oxytocin correlates with affiliative behaviors (59, 60) and was found to buffer stress responses (34, 35). Therefore, it seems reasonable that this type of interaction can be beneficial to the child. However, the positive effects will outweigh the risks only if parents are trained to recognize the dogs signaling and are able to guide interactions in such a way that the dog also enjoys the interaction, and recognize when the dog needs a rest. Caregiving behaviors also activate the oxytocin system and are generally associated with positive emotions (5, 61). Together with benign behaviors these interactions likely promote attachment to the pet (62) and pet attachment was found to be more important than ownership in terms of developmental benefits in many studies (4). “Indirect” dog care activities such as preparing food or food stuffed toys or getting an additional blanket for resting times or the dogs leash before a walk can be carried out safely while the dog is separated by a baby gate. Under the supervision of dog-competent parents, it might be possible to involve even preschool children more in dog care activities. However, possible risk factors have to be considered every time the child is involved in the activity. Examples for relevant aspects are: could it cause pain to the dog; might the dog feel threatened; are valuable resources of the dog involved; what is the dog’s emotional and health state at present; how compliant is the child, etc. These same aspects have to be considered for every child–dog interaction and supervision of children in the studied age group is recommended at all times (33). Involving children in activities that can induce pain, fear, anger, or high arousal (negative and positive!) in the dog or that may startle the dog should always be avoided.

Although the current evidence does not allow definite conclusions, positive effects of young children growing up with pets and in particular with a dog have been shown for empathy and perspective taking, self-esteem, anxiety, cognition and problem solving, social competence, and positive attitudes toward pets (4). Parental guidance in pet care promoted pet attachment and positive effects on cognition in 10- to 14-year-old children (63). A similar effect with younger children would also be plausible: teaching them about dog behavior and needs and carefully guiding interactions might enhance positive effects of having a dog in the family. Parental input was found to be particularly important for retention of knowledge up to the age of 3 years (31). Presumably, the best outcome can be expected when children and dogs are supervised and guided by knowledgeable, emphatic, and responsible adults.

Parent attitudes to supervision were highly correlated with and probably shape the child’s behavior toward the dog. Especially child behaviors potentially aversive for the dog were highly related to parent attitudes. As the score on the attitude subscale “allow unsafe behaviors” was not related to the child’s age, we assume that allowing or not allowing unsafe behaviors might be based on general beliefs. These beliefs could be targeted by dog bite prevention programs. It seems important to teach parents that no interaction can be considered safe as everything depends on the circumstances. To be effective, dog bite prevention programs should be widespread and caregivers should be engaged at early stages of the child–dog relationship. Fatal incidents with dogs can already occur in newborns and were most common in children of up to 4 years (13, 64). Besides involvement of caregivers in dog bite prevention, there are programs that attempt to teach children from the age of 3 years on safety with dogs (29, 31, 65) and how to read dog body language (21, 30). Evaluating behavior changes with dogs in real-life contexts and low participation in the programs seem to be some of the challenges that still have to be resolved (31, 32, 65, 66).

Recommendations on the age of the child at which it seems safe to get a dog differ: one example says that the child should be at least 4 years old (67), another that a combination of a dog younger than 1 year and a child younger than 5 years should be avoided (48) or that having a dog should be postponed until the children are of school age (18). However, it seems difficult to give a global recommendation, as everything depends on the individuals involved and their ability to handle the situation.

## Conclusion

Interactions between children and family dogs begin very early in a child’s life and most of the behaviors are already reported at high levels in 1-year-old children. Supervision by caregivers seems to have a strong influence on the behavior of the child toward the dog. Therefore, our results underline that in the first place parents must be educated about supervision of child–dog interactions and monitoring of dog body language at a very young age of the child or ideally even before a child is born or a dog is acquired. Parents and dog owners should also learn to pay attention to contextual cues of interactions and which interactions should be totally avoided. Another important aspect is that management measures to increase safety when active supervision is not possible should be promoted. Regarding the dog, notably those that did not grow up with children might need more time to adapt to living with children and they should be carefully observed for signs of fear or stress in particular when the child begins to explore the environment on its own. The results of this exploratory study further underline the need for an early dog bite prevention approach directed toward the caregivers that is tailored to the child’s age and to particular needs of individuals involved.

## Ethics Statement

The study did not include live animals, therefore the ethics committee of the University of Veterinary Medicine Vienna stated that the study did not require a vote. The study involved self-selected anonymous human respondents on aspects of living with children and dogs, therefore the ethics committee of the University of Medicine Vienna stated that the study did not require a vote. The survey included no mandatory questions and participants could quit at any time.

## Author Contributions

The study was designed by CA, AB, and JT. The data collection was carried out by CA. The data were analyzed by CA. The article was drafted by CA. It was revised and critically discussed by AB and JT. All authors approved the final version and are accountable for all aspects of the work.

## Conflict of Interest Statement

The authors declare that the research was conducted in the absence of any commercial or financial relationships that could be construed as a potential conflict of interest.
