# Supplementary material for: Caregiver Reports of Interactions between Children up to 6 Years and Their Family Dog—Implications for Dog Bite Prevention
Source: Front Vet Sci. 2017 Aug 30;4:130. doi: 10.3389/fvets.2017.00130 (PMC5582199; doi:10.3389/fvets.2017.00130)
Supplement: Supplementary file 1 [file Image_1.PDF]

Supplementary Material

Caregiver reports of interactions between children up to six years and their family dog – implications for dog bite prevention

Christine Arhant*, Andrea Beetz, Josef Troxler

*** Correspondence:** Corresponding Author: Christine.Arhant@vetmeduni.ac.at

Supplementary Figures

These supplementary figures are based on data of a cross-sectional online survey on child-dog interactions in 402 caregivers living with a child up to six years old and a family dog. Each interaction was rated by the participants on a six-point scale ranging from “Never” = 1 to “Very often” = 6 and was grouped according to child age for presentation in the supplementary figures. Mean numbers of valid answers in the child age categories were for child or dog behaviors respectively: up to 6 month = 29/28, 6 to 12 month = 69/69, 1.5 to 2 years = 78/74, 2.5 to 3 years = 68/66, 3.5 to 4 years = 47/44, 4.5 to 5 years = 37/37, 5.5 to 6 years = 35/33.

1 Child - benign

1.1 Speak to dog

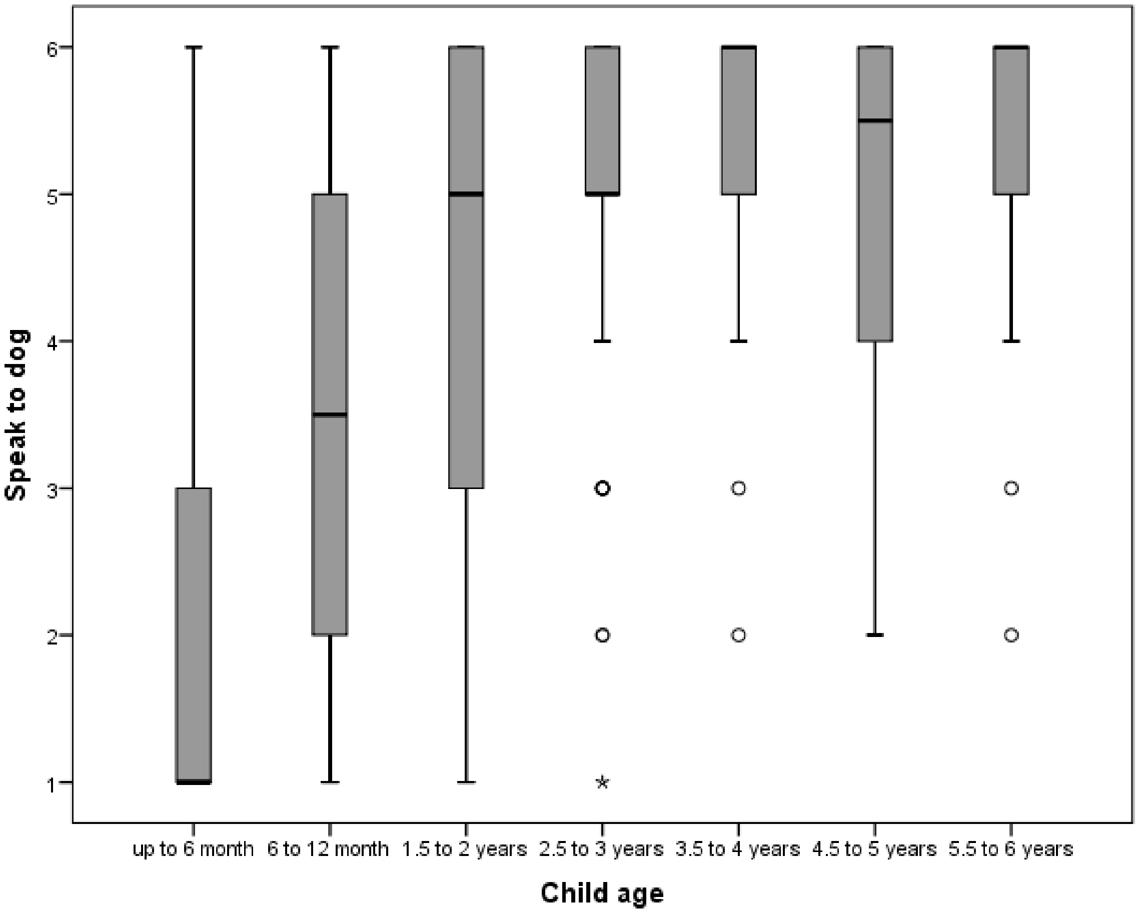

Supplementary Figure 1: Frequency of speaking to the dogs grouped by age of child ("Never" = 1 to "Very often" = 6)

1.2 Pet dog on body

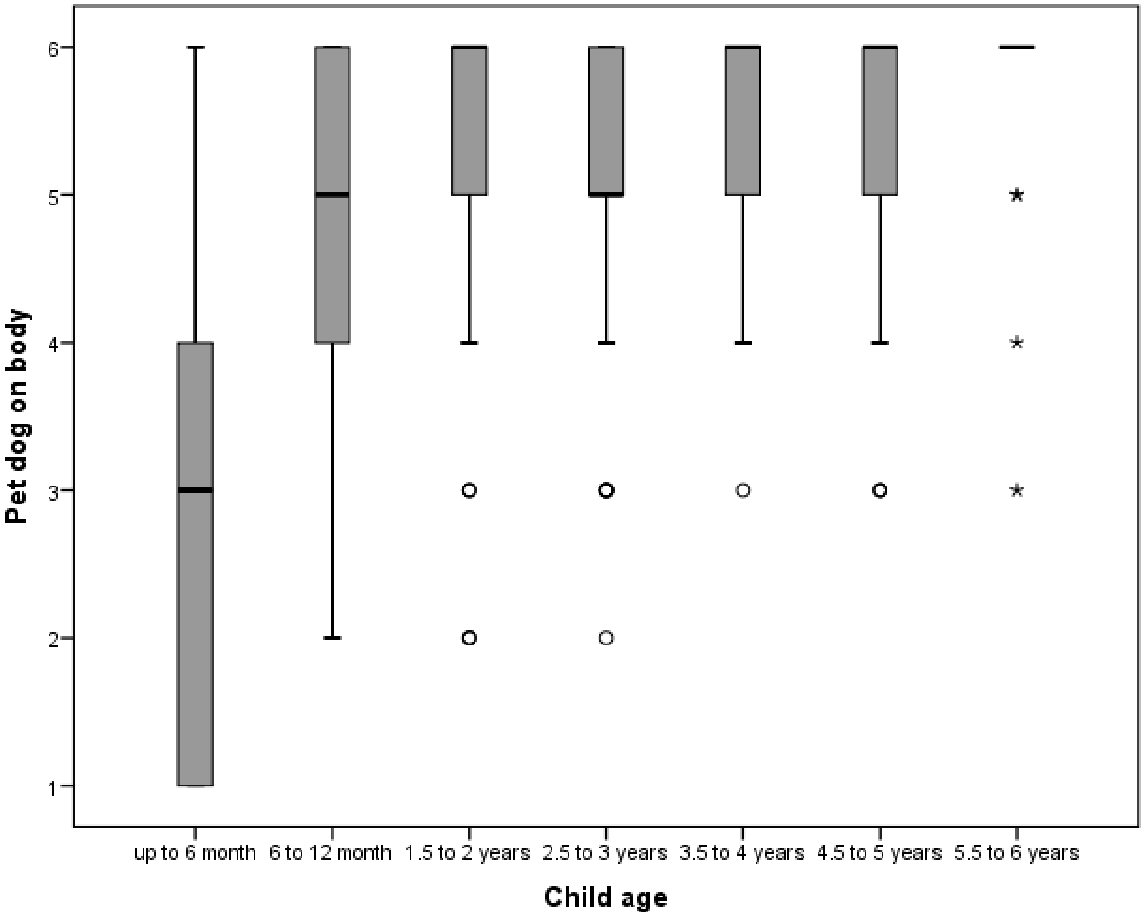

Supplementary Figure 2: Frequency of petting the dog on the body grouped by age of child ("Never" = 1 to "Very often" = 6)

1.3 Pet dog on head

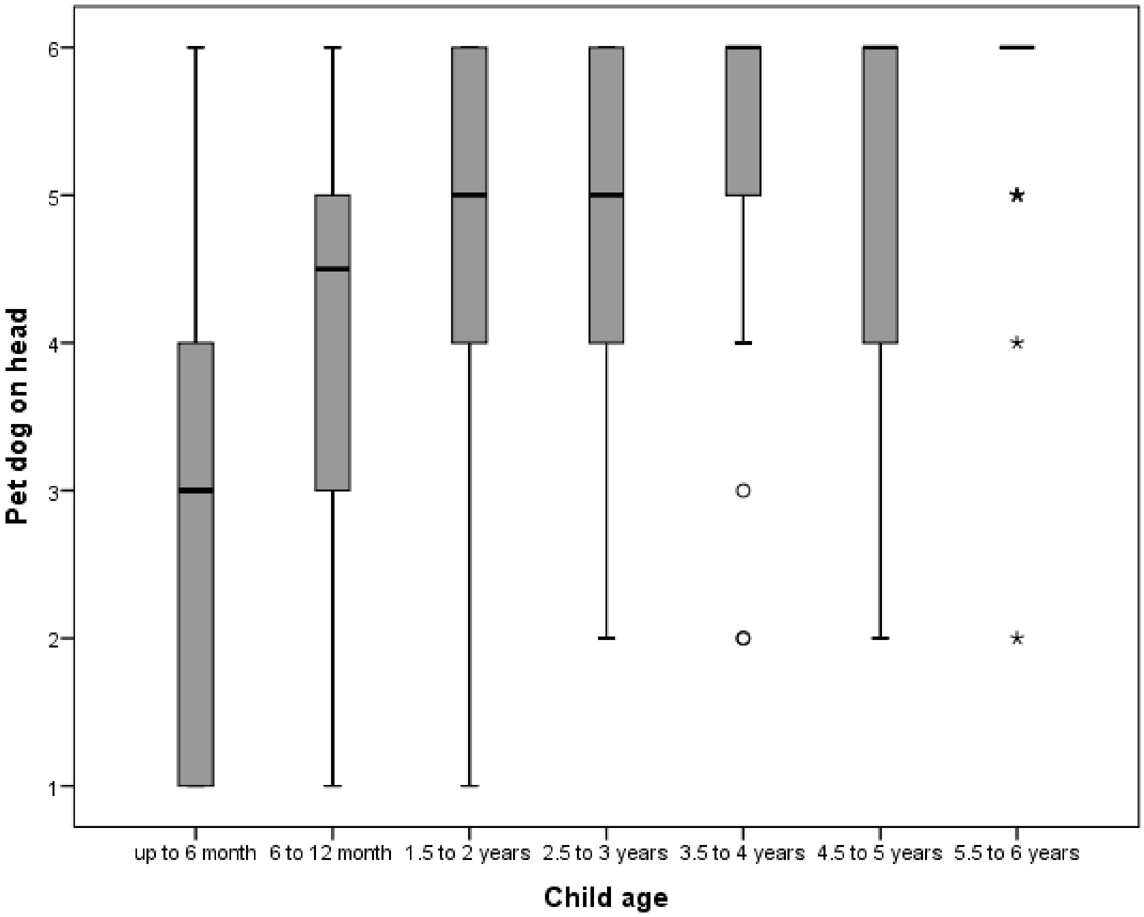

Supplementary Figure 3: Frequency of petting the dog on the head grouped by age of child ("Never" = 1 to "Very often" = 6)

1.4 Hug dog

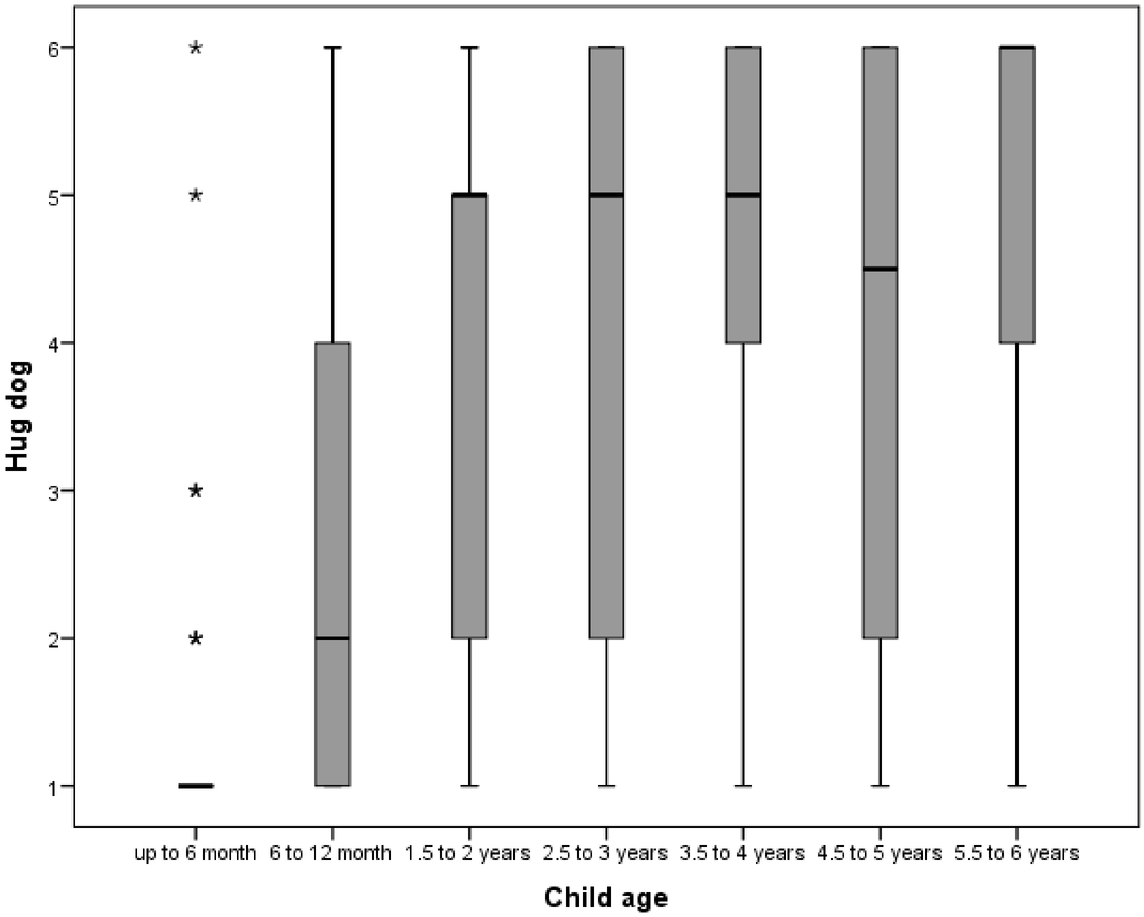

Supplementary Figure 4: Frequency of hugging the dog grouped by age of child (“Never” = 1 to “Very often” = 6)

1.5 Kiss dog

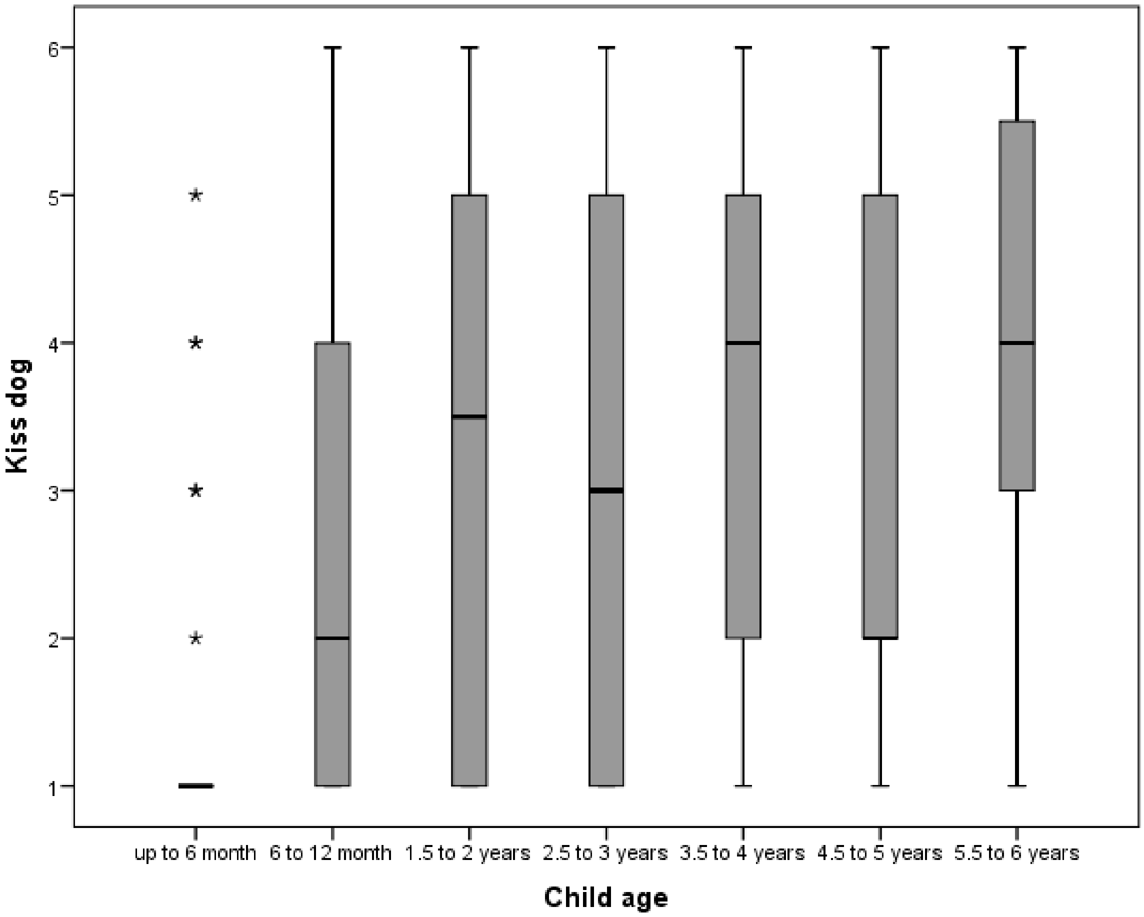

Supplementary Figure 5: Frequency of kissing the dog grouped by age of child ("Never" = 1 to "Very often" = 6)

1.6 Reach for dog

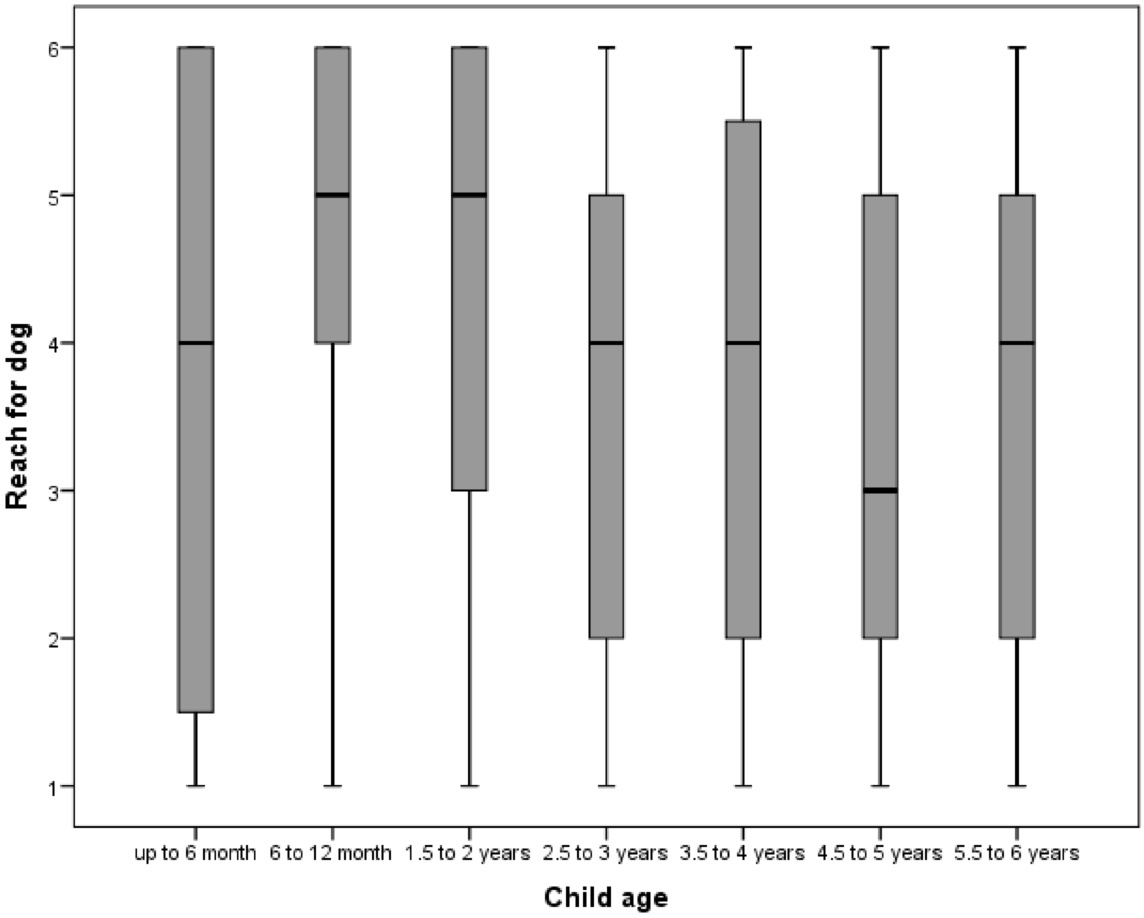

Supplementary Figure 6: Frequency of reaching for the dog grouped by age of child (“Never” = 1 to “Very often” = 6)

1.7 Approach or follow dog

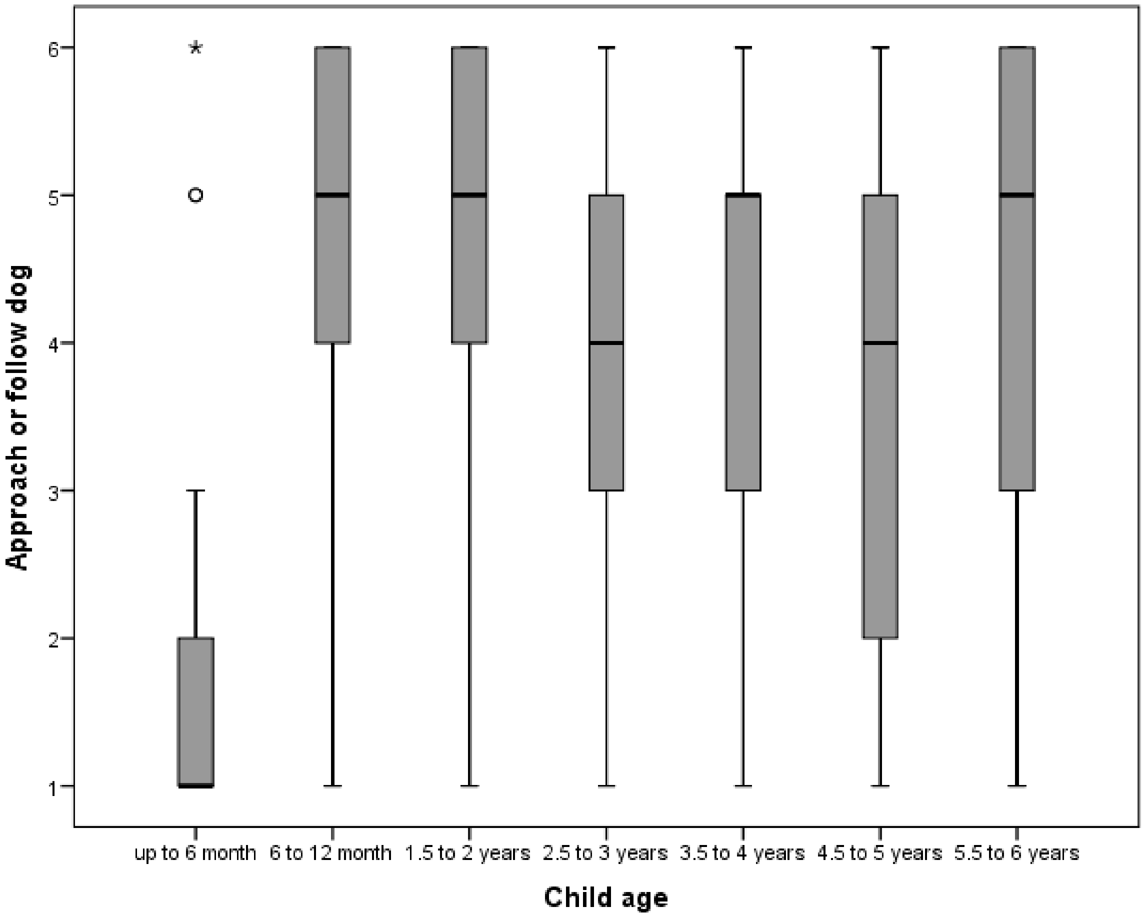

Supplementary Figure 7: Frequency of approaching or following the dog grouped by age of child (“Never” = 1 to “Very often” = 6)

2 Child - resting

2.1 Wake sleeping dog

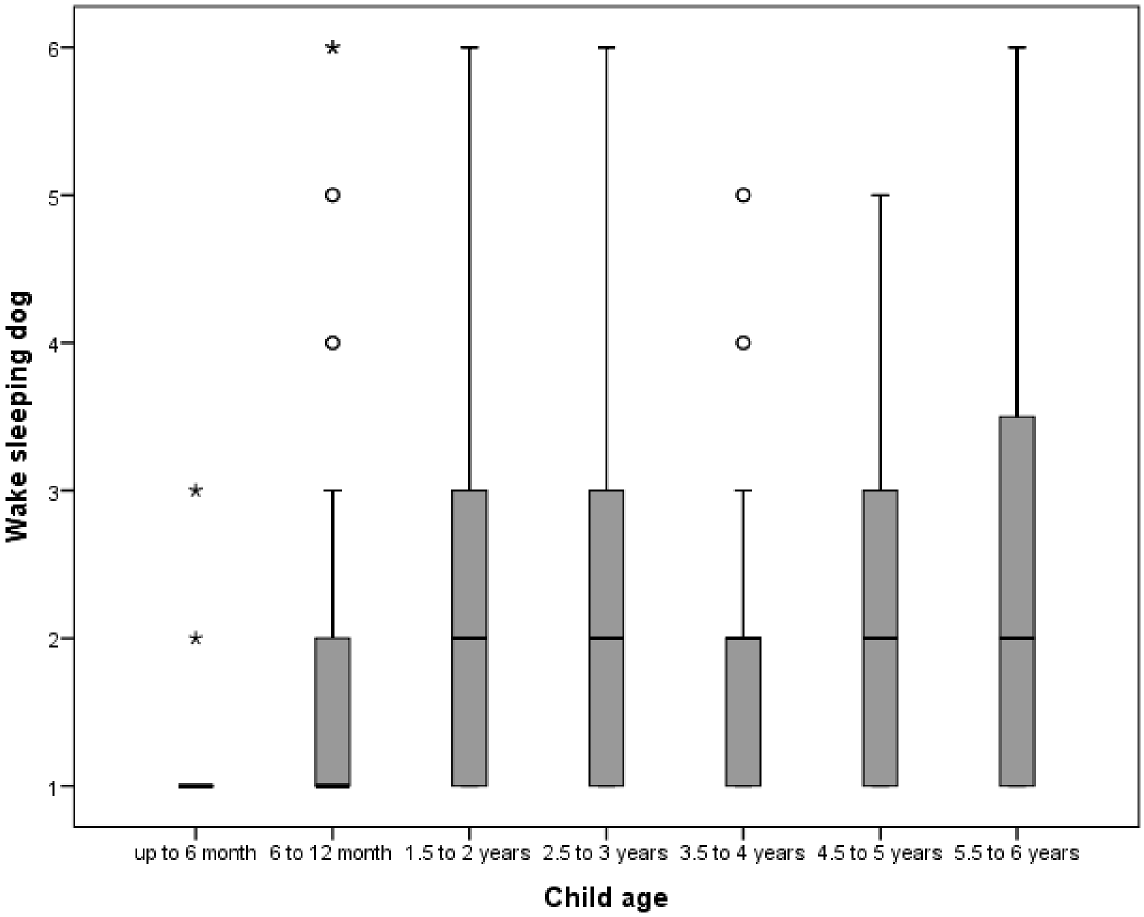

Supplementary Figure 8: Frequency of waking the sleeping dog grouped by age of child (“Never” = 1 to “Very often” = 6)

2.2 Lay down near to resting dog

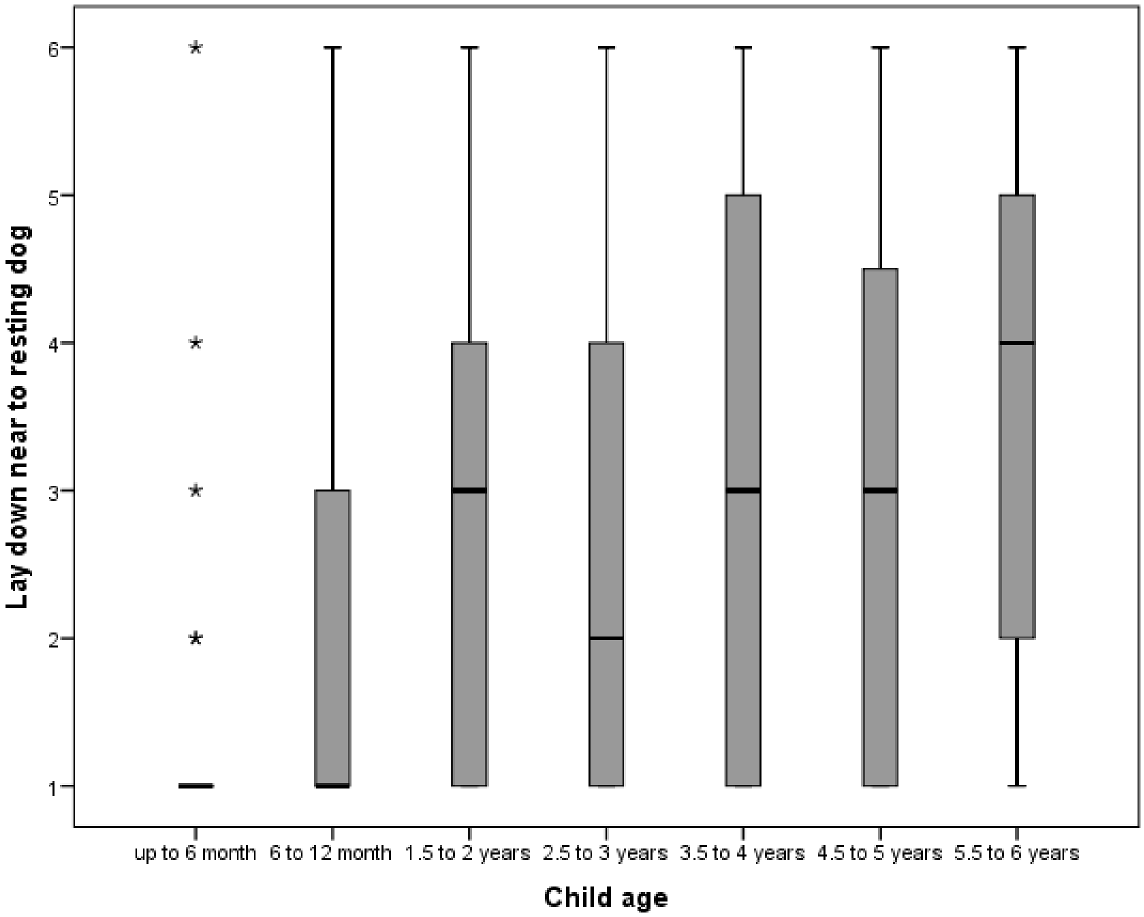

Supplementary Figure 9: Frequency of lying down near to the resting dog grouped by age of child (“Never” = 1 to “Very often” = 6)

2.3 Leave resting dog alone

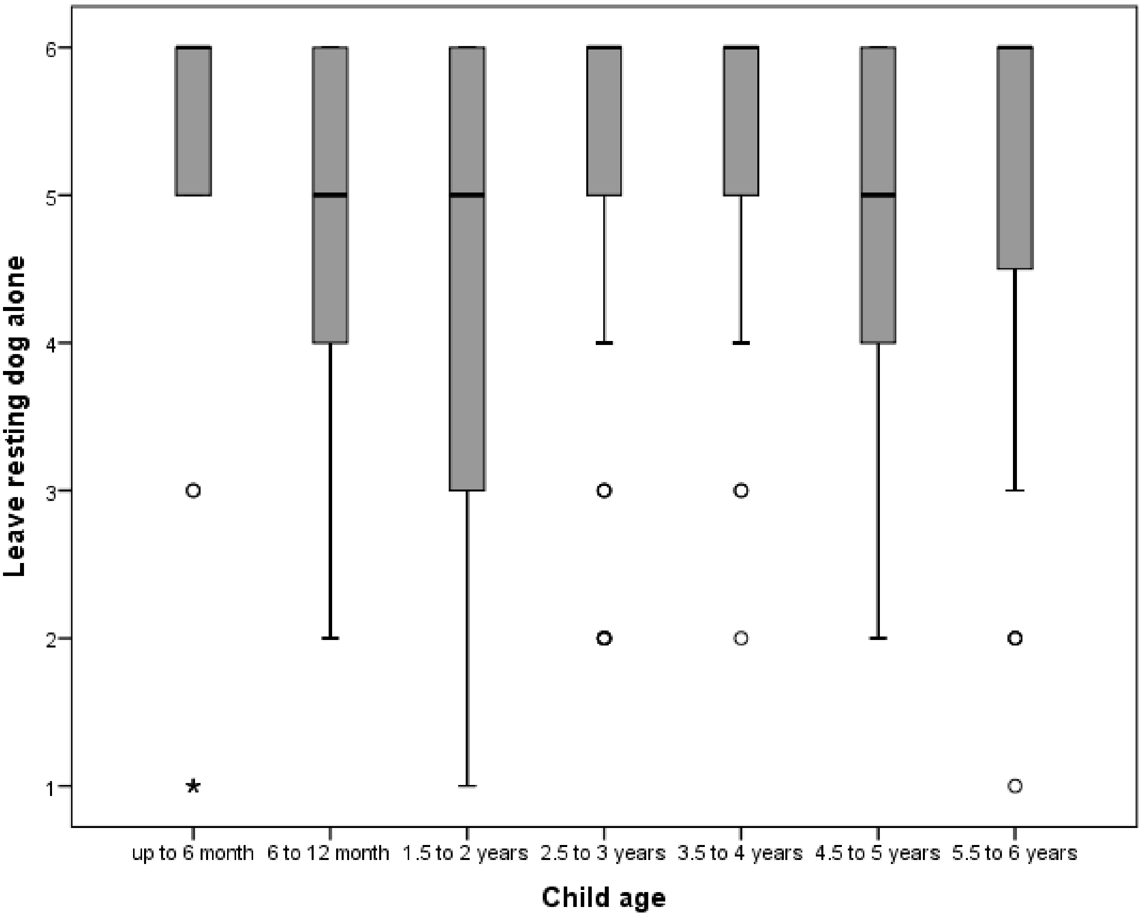

Supplementary Figure 10: Frequency of leaving the resting dog alone grouped by age of child ("Never" = 1 to "Very often" = 6)

3 Child - resources

3.1 Attempt to take away dog food or bowl

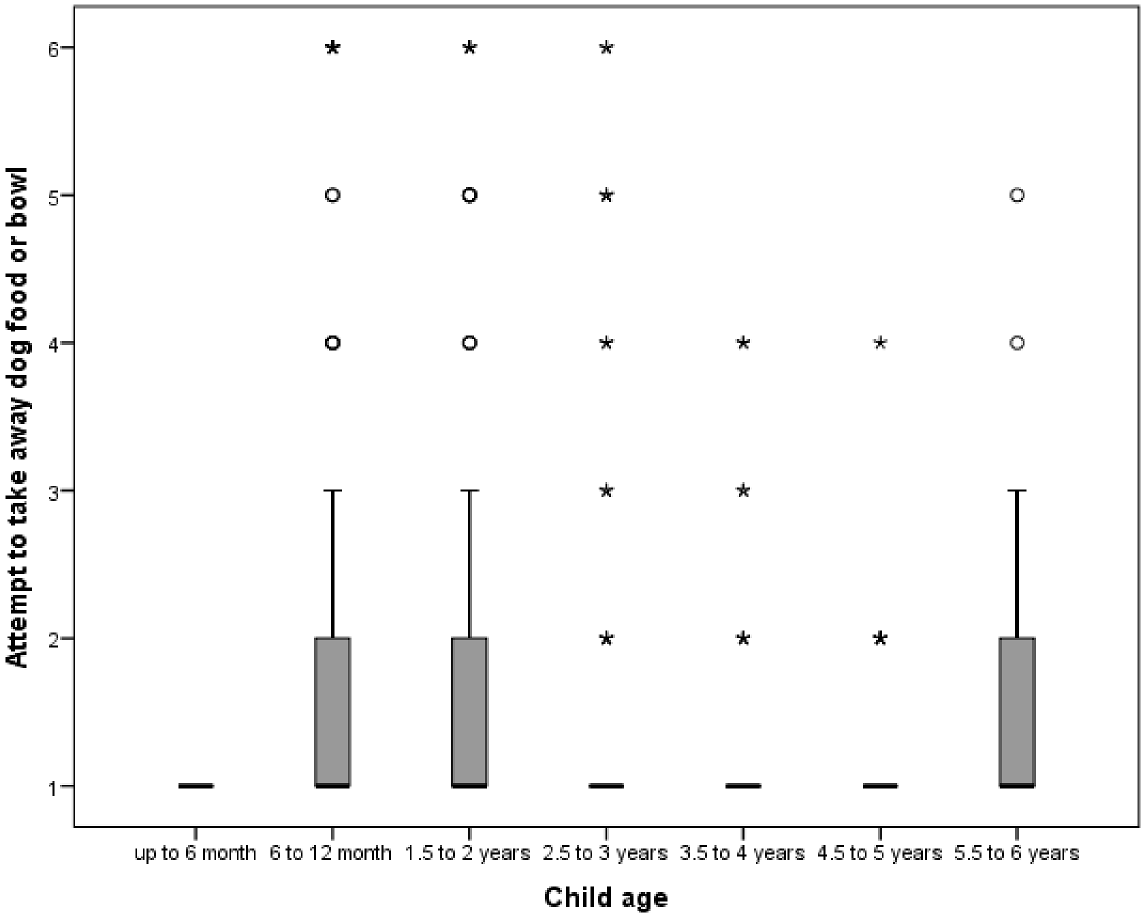

Supplementary Figure 11: Frequency of attempts to take away dog food or the food bowl from the feeding dog grouped by age of child (“Never” = 1 to “Very often” = 6)

3.2 Attempt to pet feeding dog

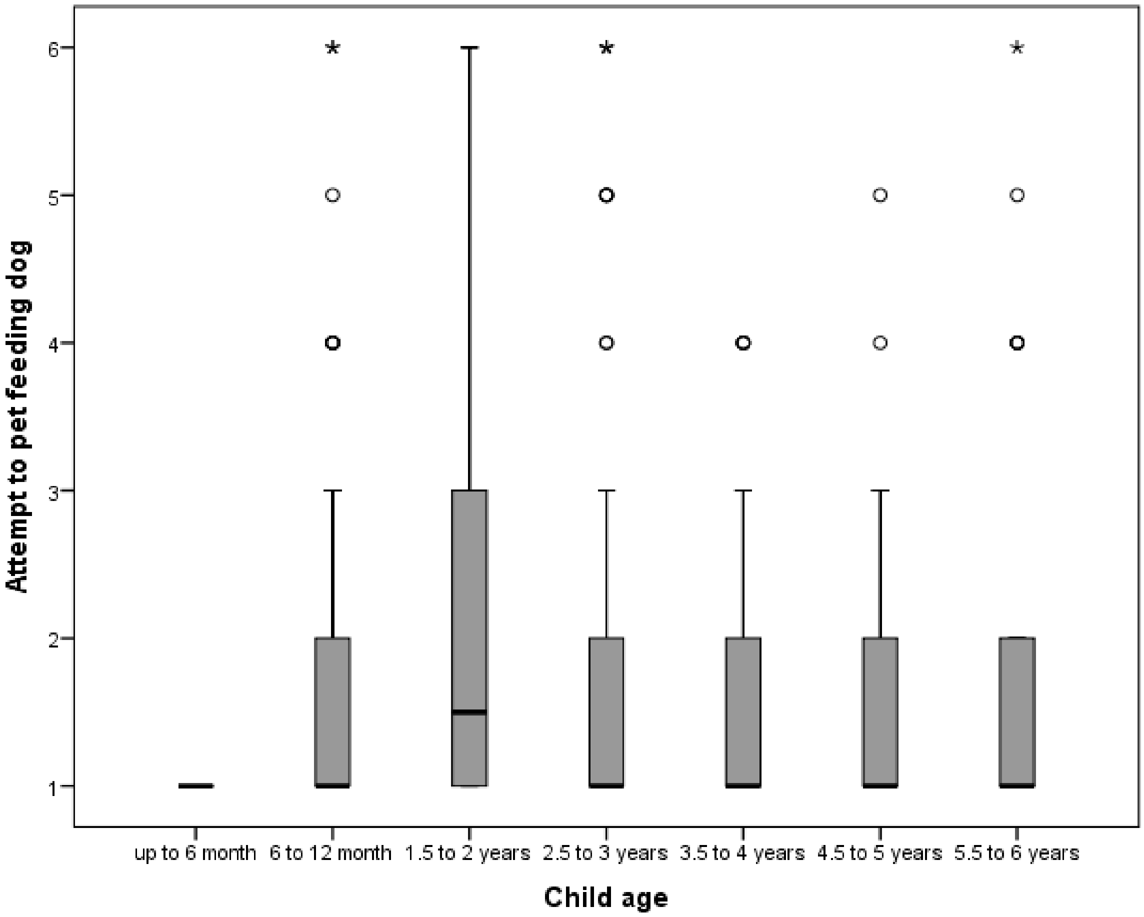

Supplementary Figure 12: Frequency of attempts to pet the feeding dog grouped by age of child ("Never" = 1 to "Very often" = 6)

3.3 Take child toys from dog

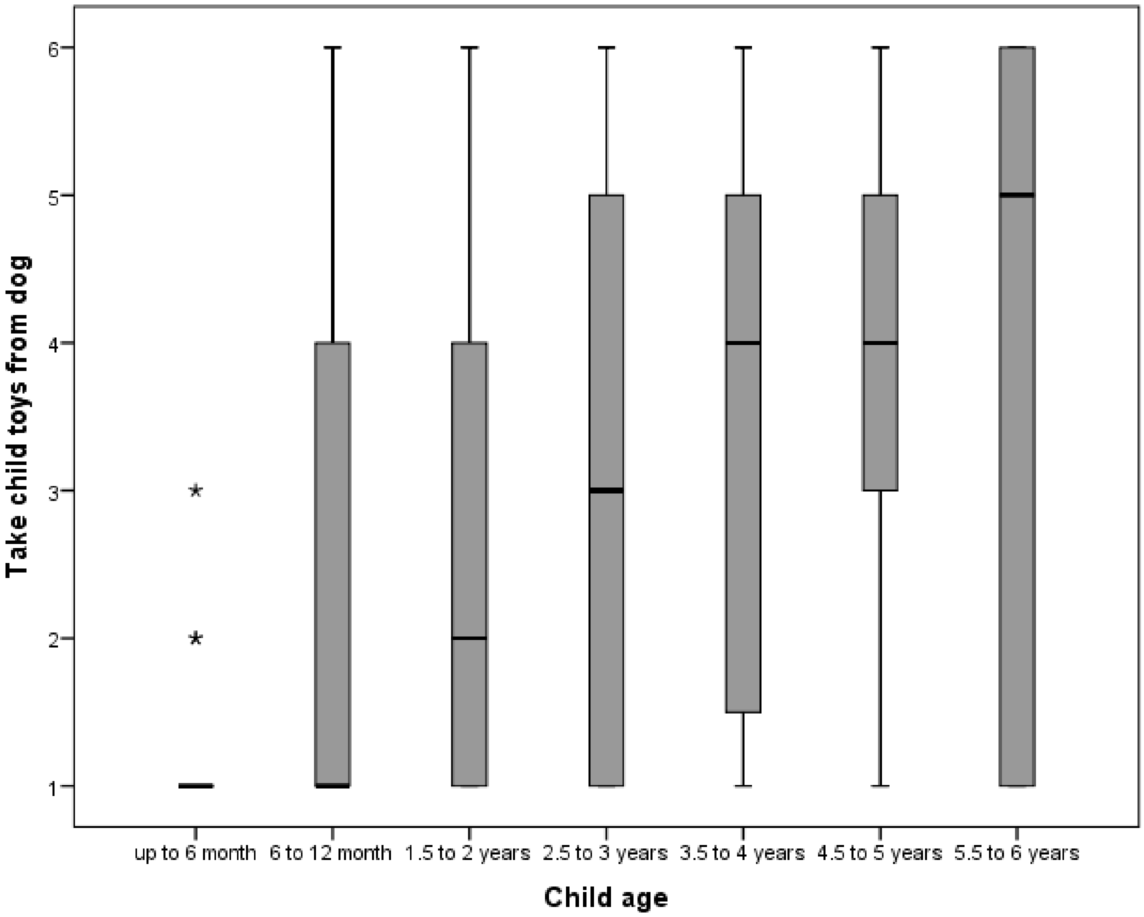

Supplementary Figure 13: Frequency of taking child toys from the dog grouped by age of child ("Never" = 1 to "Very often" = 6)

3.4 Attempt to take dog toys/chews from dog

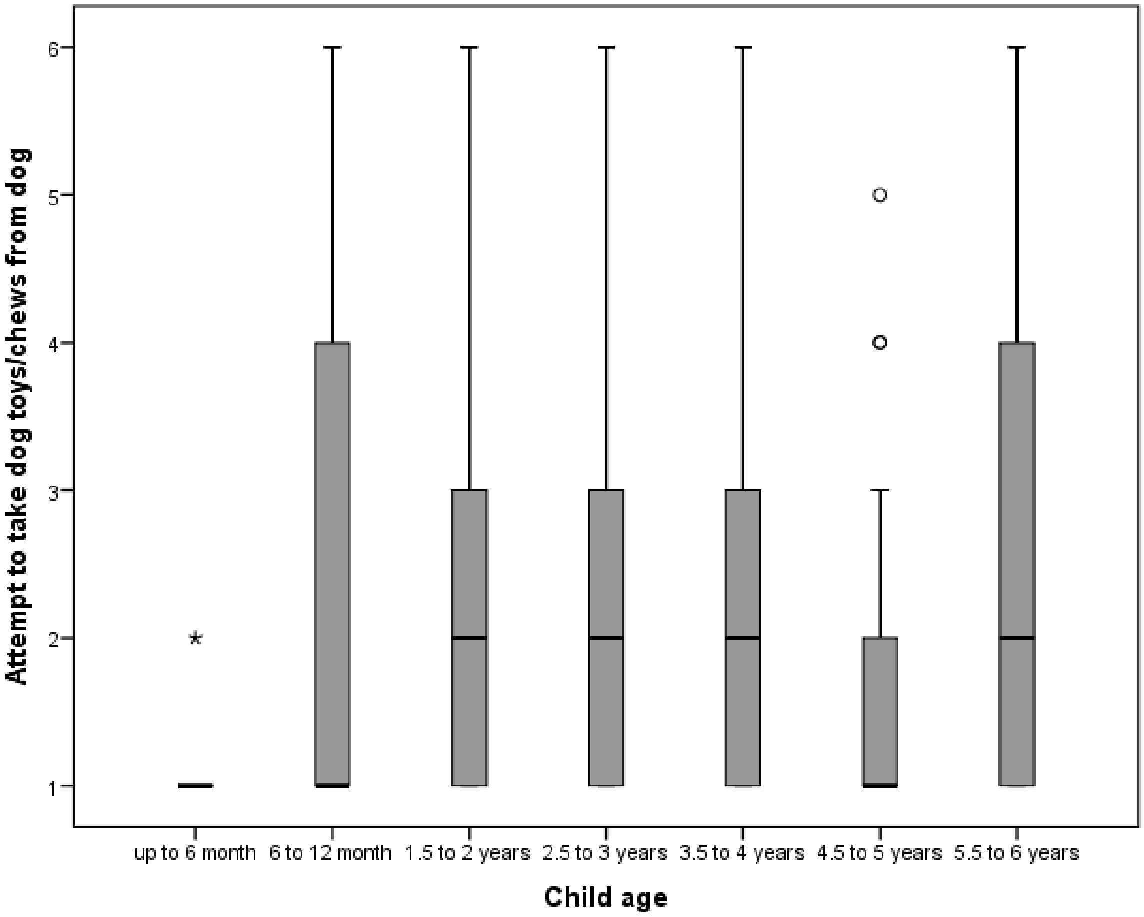

Supplementary Figure 14: Frequency of attempts to take dog toys or chews from the dog grouped by age of child ("Never" = 1 to "Very often" = 6)

4 Child – aversive non-painful

4.1 Restraint by collar

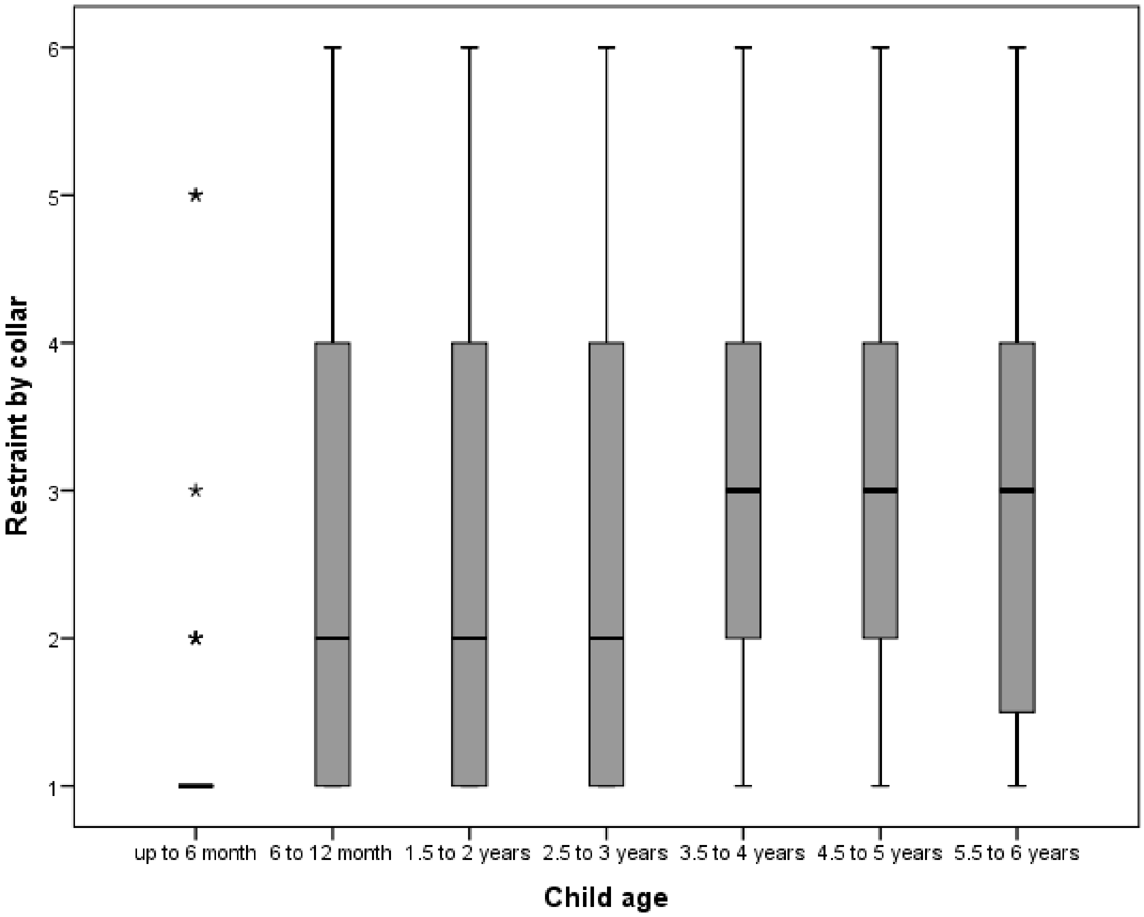

Supplementary Figure 15: Frequency of restraining the dog by the collar grouped by age of child (“Never” = 1 to “Very often” = 6)

4.2 Grooming

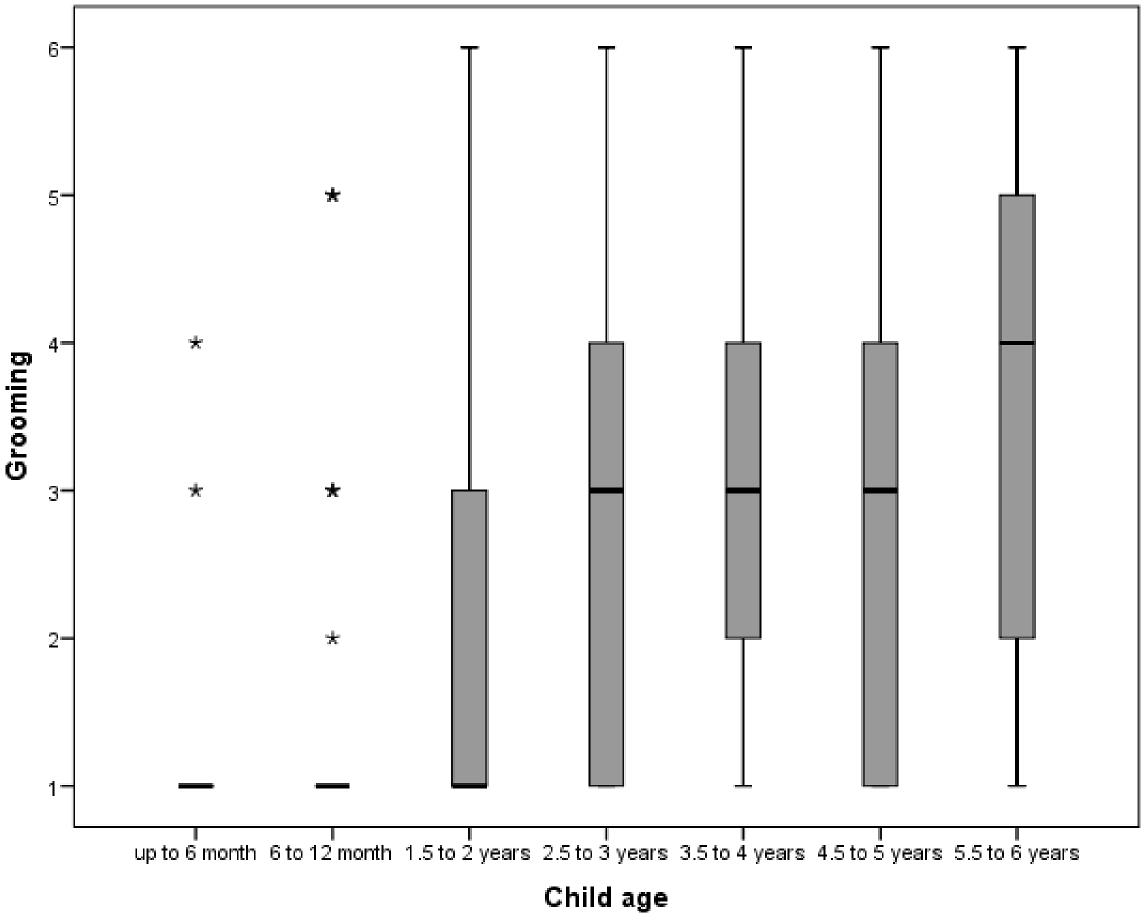

Supplementary Figure 16: Frequency of grooming the dog grouped by age of child (“Never” = 1 to “Very often” = 6)

4.3 Child yells or screams during interaction

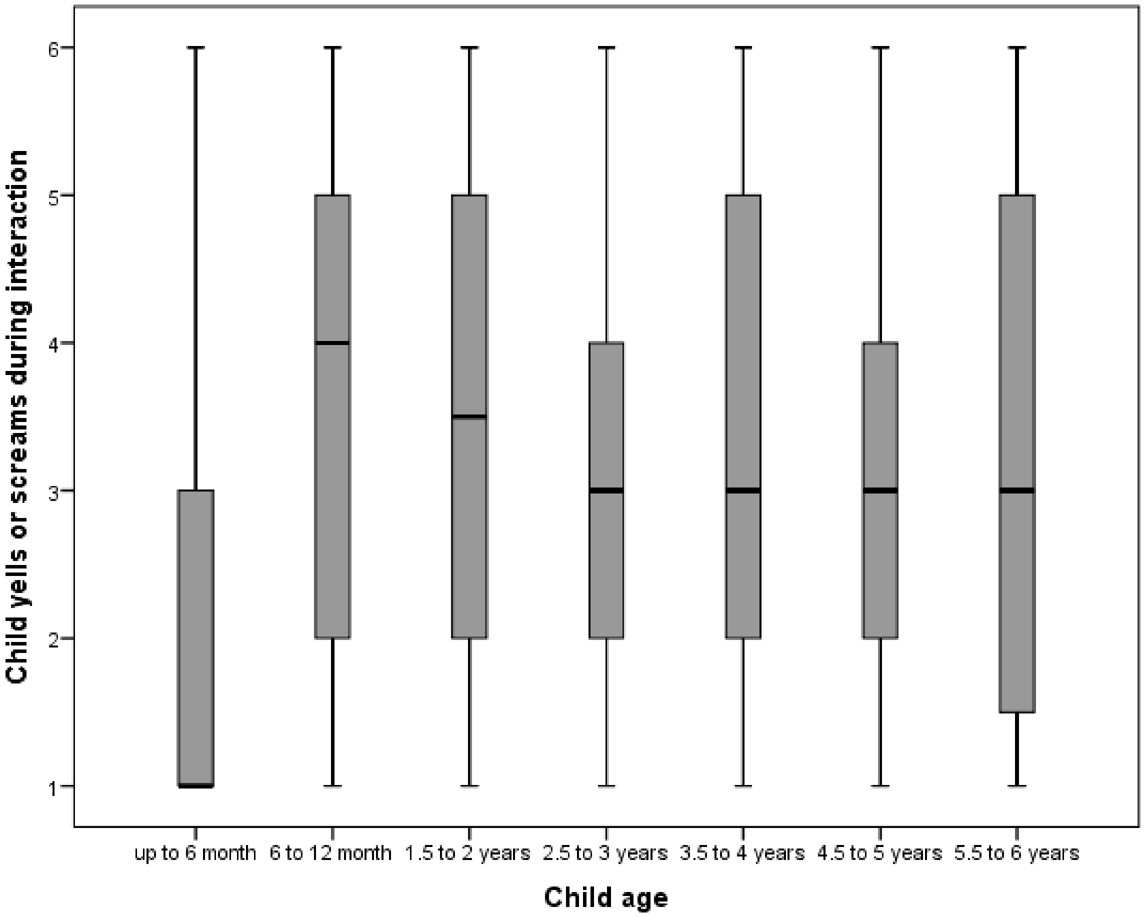

Supplementary Figure 17: Frequency of yelling or screaming during an interaction with the dog grouped by age of child (“Never” = 1 to “Very often” = 6)

4.4 Verbal scolding

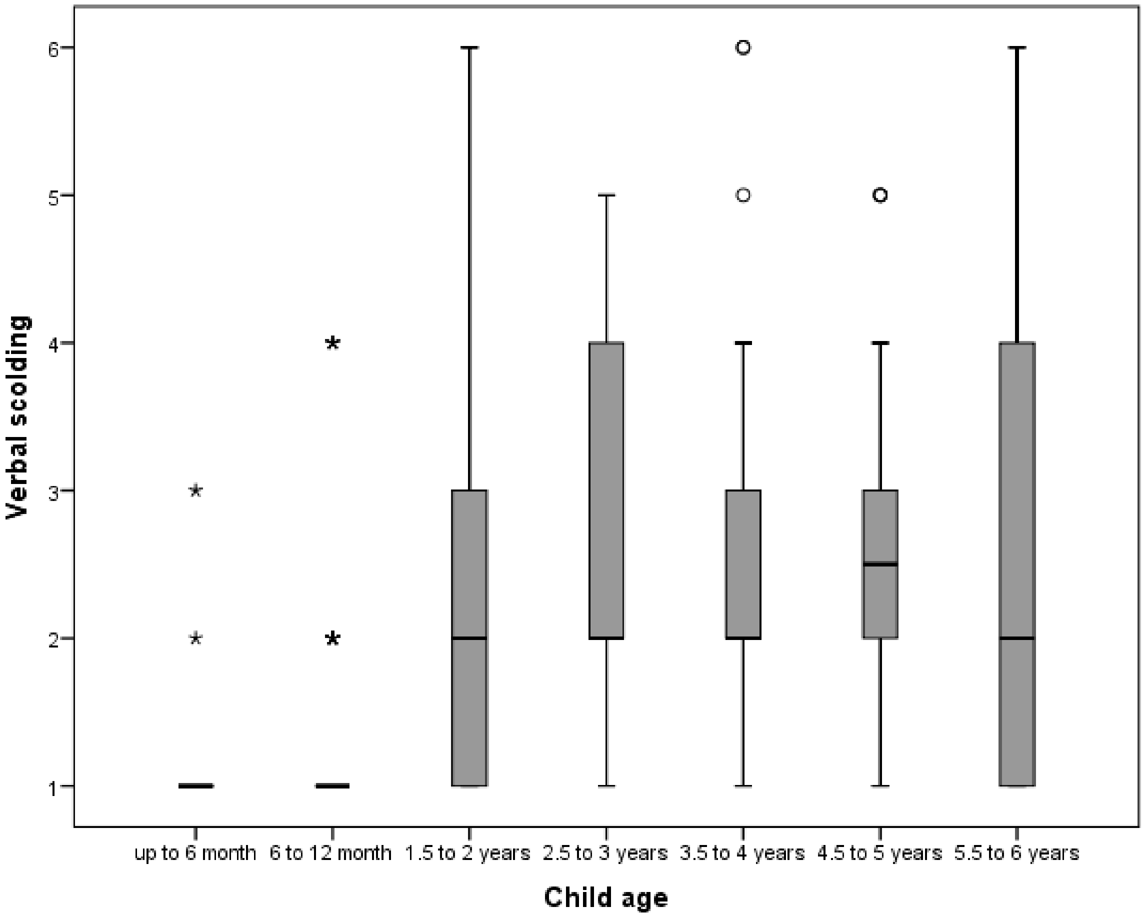

Supplementary Figure 18: Frequency of verbally scolding the dog grouped by age of child (“Never” = 1 to “Very often” = 6)

4.5 Dress dog

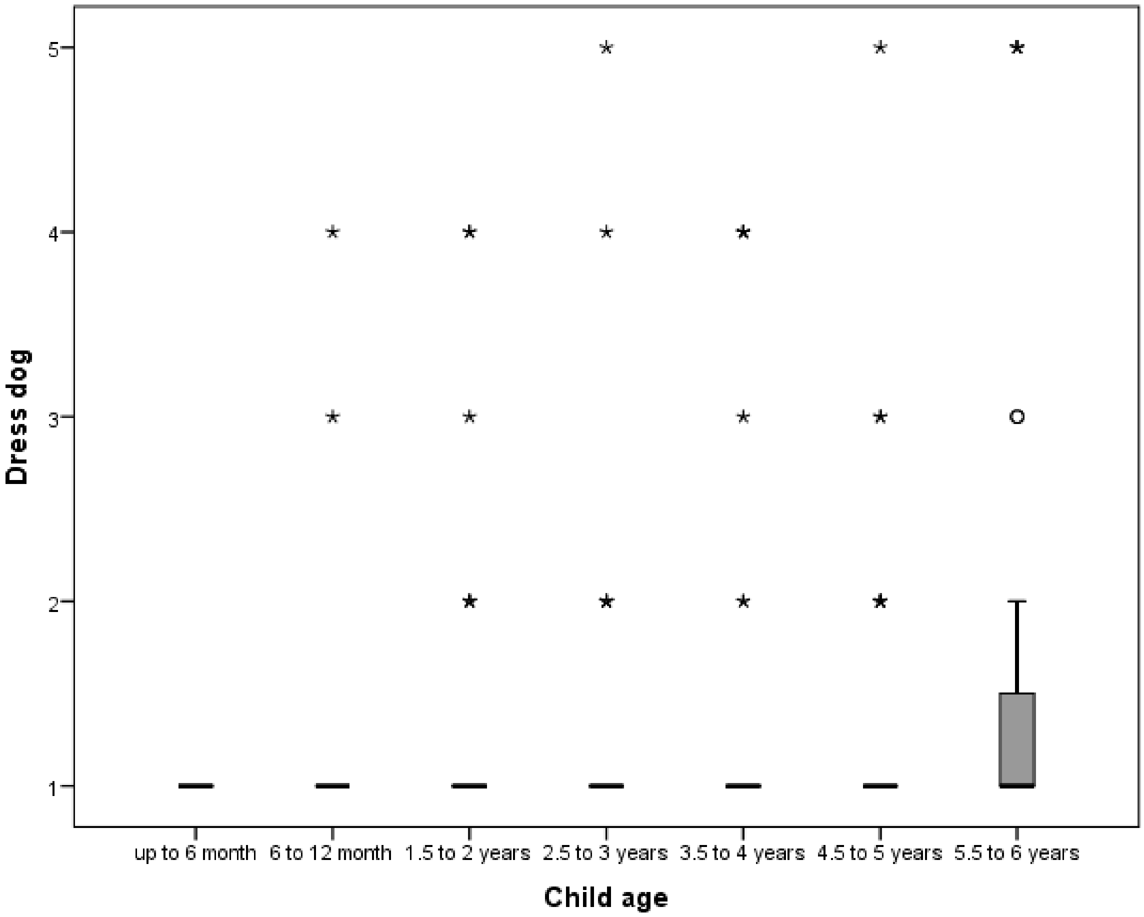

Supplementary Figure 19: Frequency of dressing the dog grouped by age of child (“Never” = 1 to “Very often” = 6)

4.6 Involve dog in child play e.g. doctor game

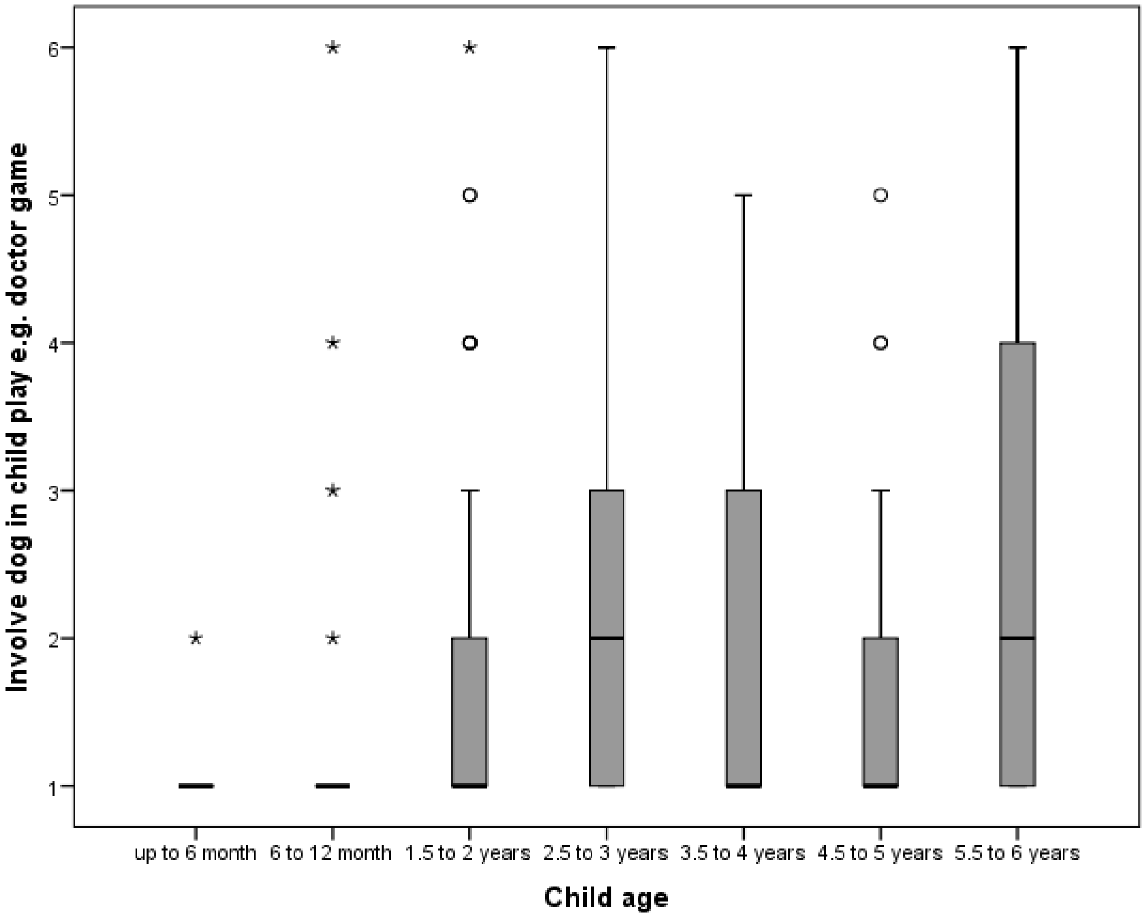

Supplementary Figure 20: Frequency of involving the dog in child play activities grouped by age of child ("Never" = 1 to "Very often" = 6)

4.7 Lift dog

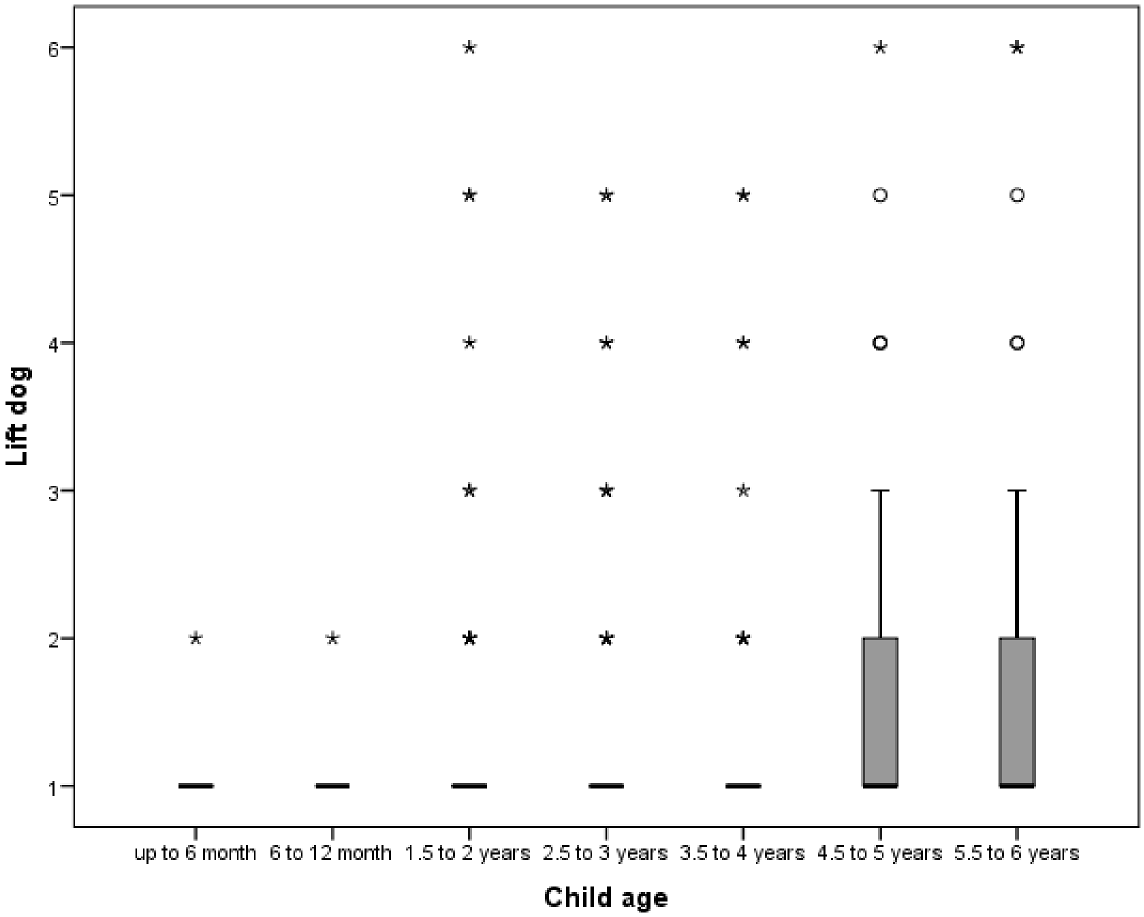

Supplementary Figure 21: Frequency of lifting the dog grouped by age of child (“Never” = 1 to “Very often” = 6)

5 Child – aversive painful

5.1 Sit, lie or ride on dog

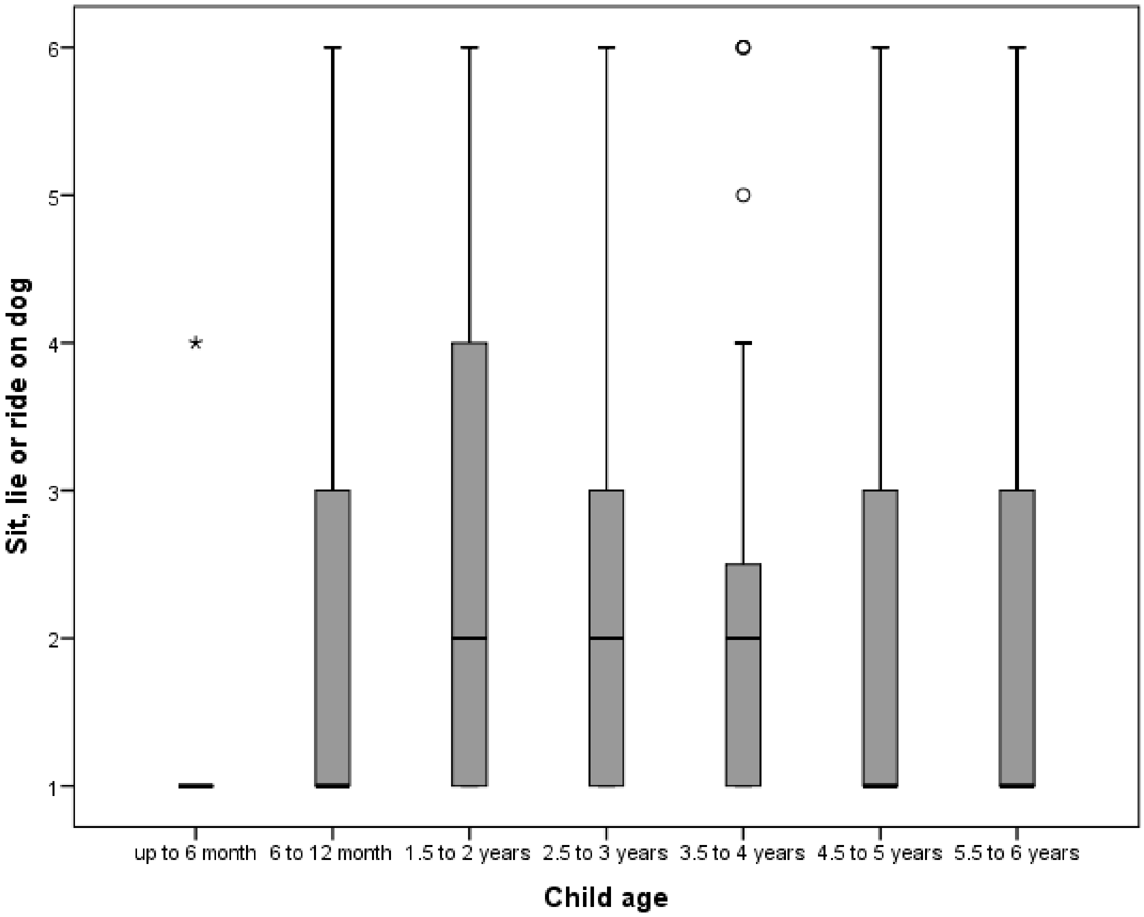

Supplementary Figure 22: Frequency of sitting, lying or riding on the dog grouped by age of child (“Never” = 1 to “Very often” = 6)

5.2 Pull on body parts of dog e.g. tail, ears

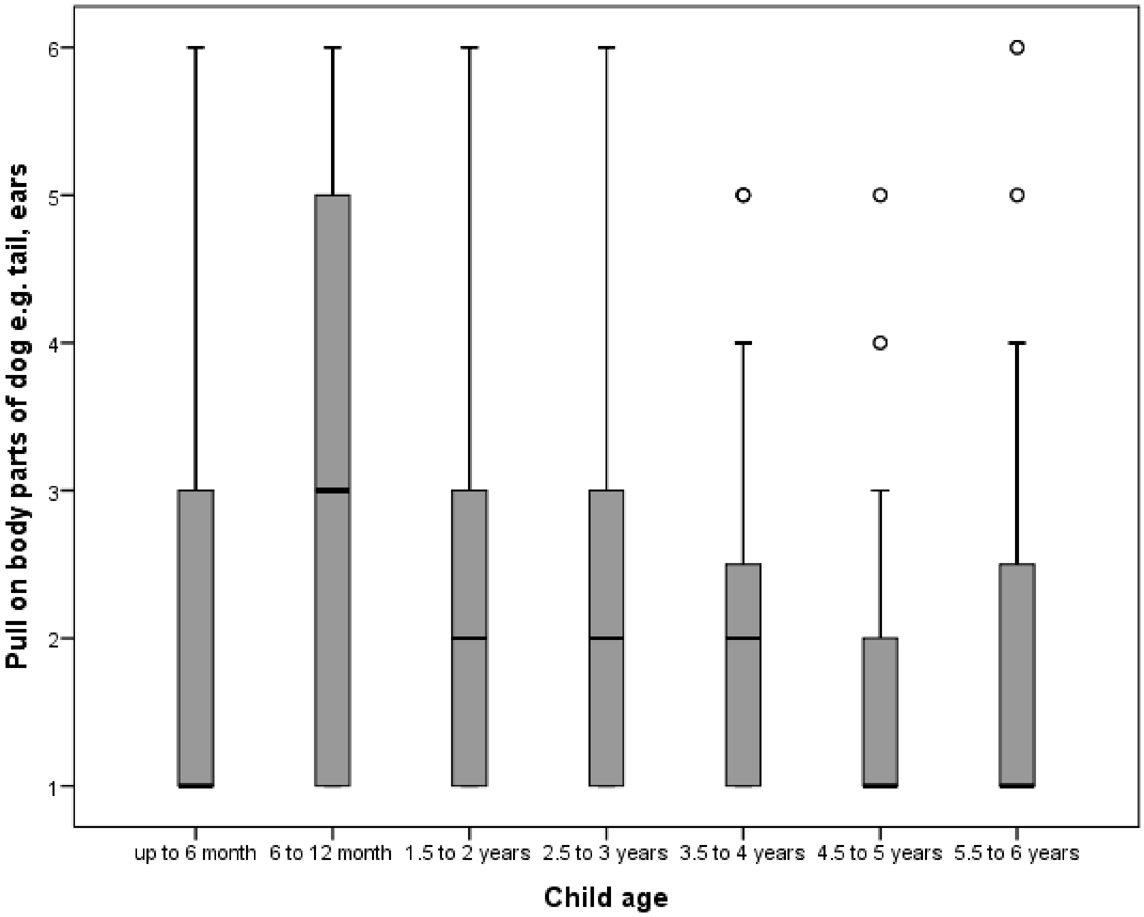

Supplementary Figure 23: Frequency of pulling on body parts of the dog grouped by age of child (“Never” = 1 to “Very often” = 6)

5.3 Inflict pain accidentally e.g. stepping on

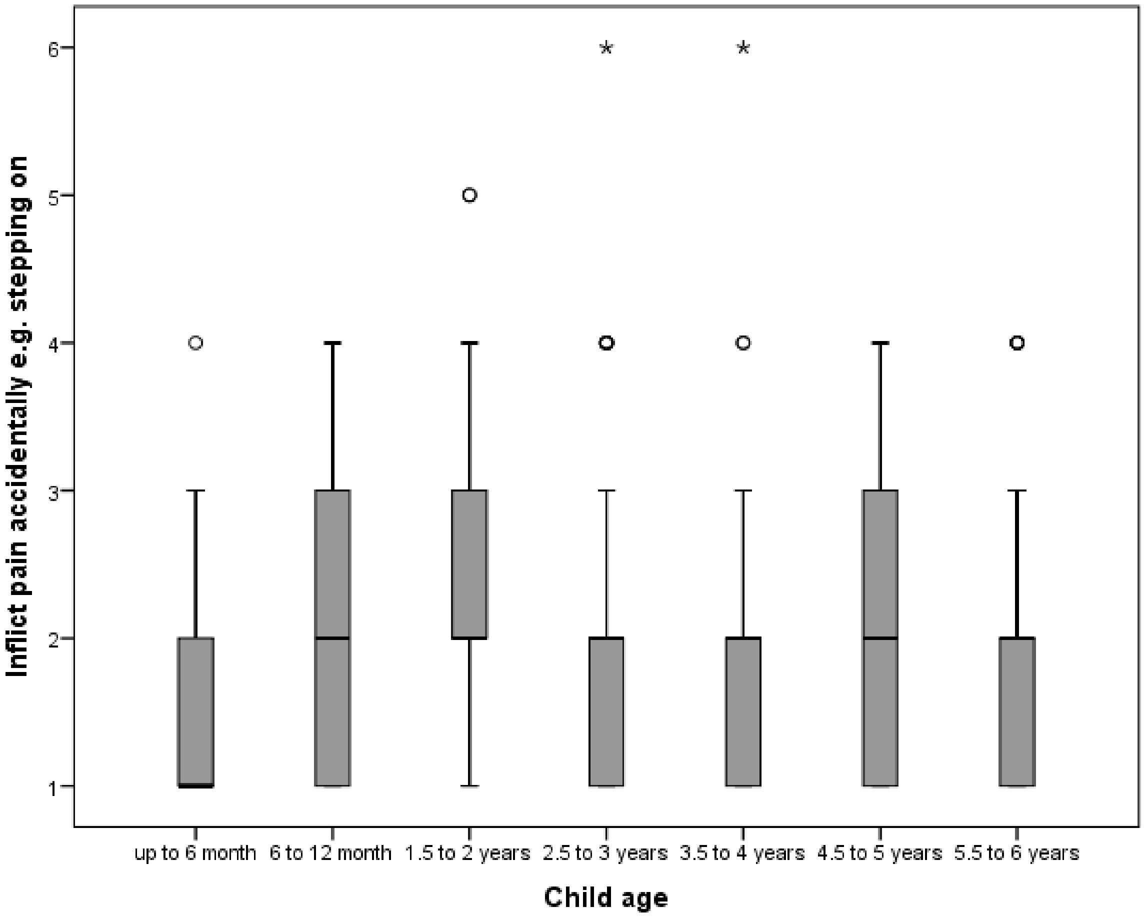

Supplementary Figure 24: Frequency of inflicting pain accidentally to the dog grouped by age of child ("Never" = 1 to "Very often" = 6)

5.4 Inflict pain deliberately e.g. hitting

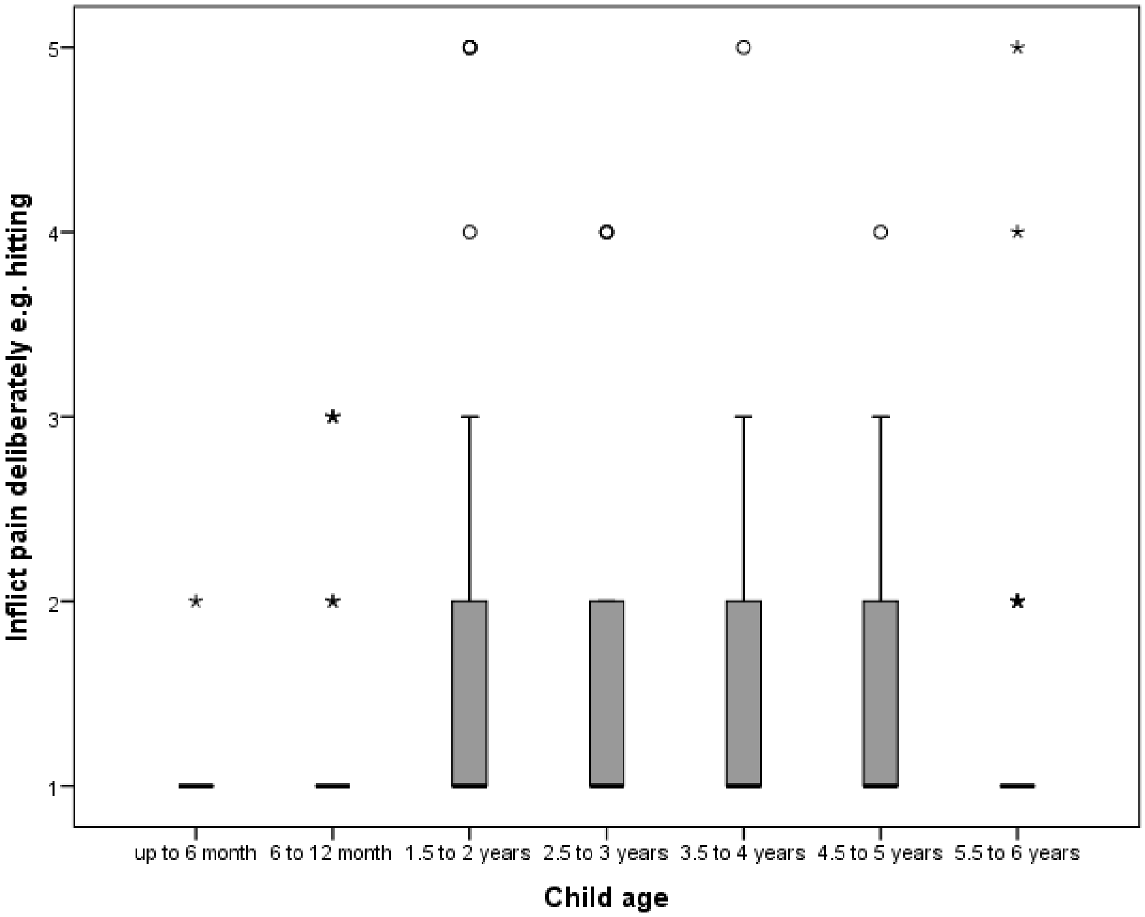

Supplementary Figure 25: Frequency of inflicting pain deliberately to the dog grouped by age of child (“Never” = 1 to “Very often” = 6)

5.5 Throw objects on dog

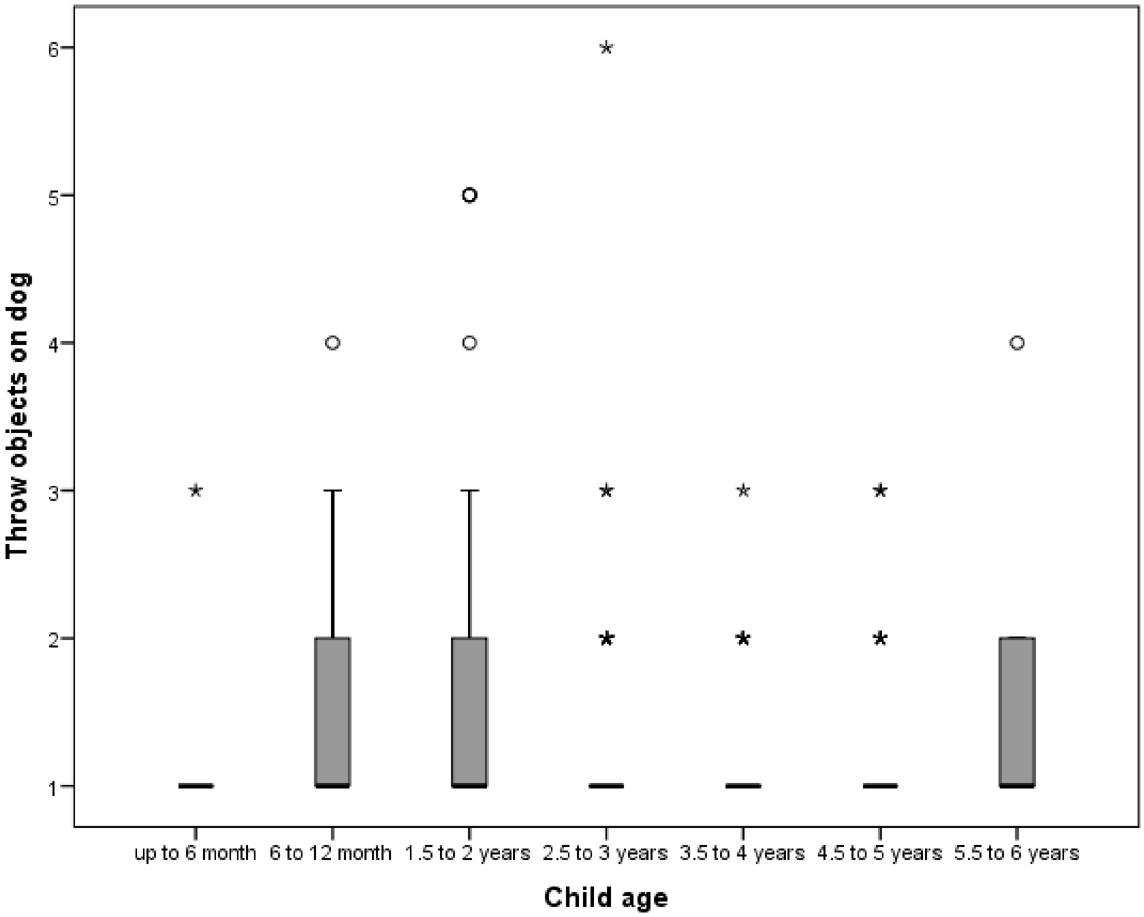

Supplementary Figure 26: Frequency of throwing objects on the dog grouped by age of child (“Never” = 1 to “Very often” = 6)

6 Child - dogcare

6.1 Feed dog

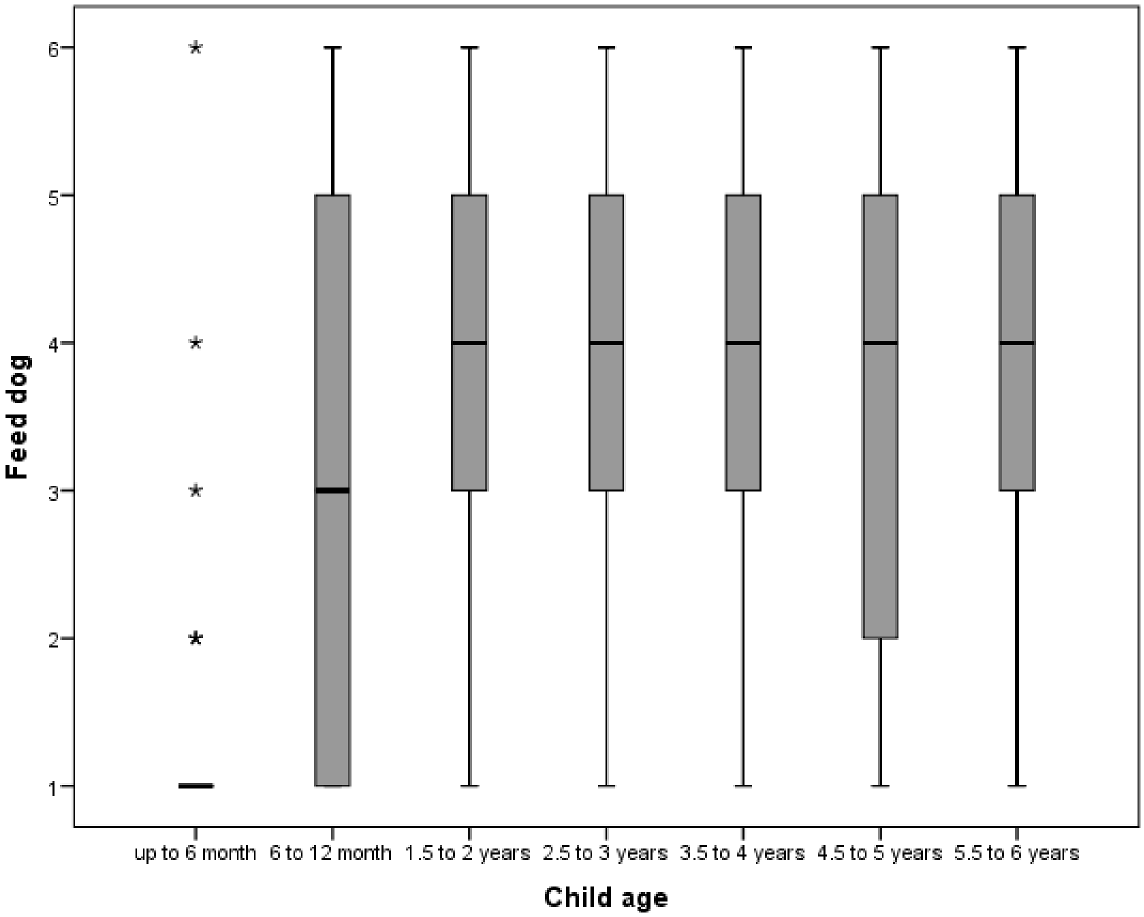

Supplementary Figure 27: Frequency of feeding the dog grouped by age of child ("Never" = 1 to "Very often" = 6)

6.2 Lead dog on leash

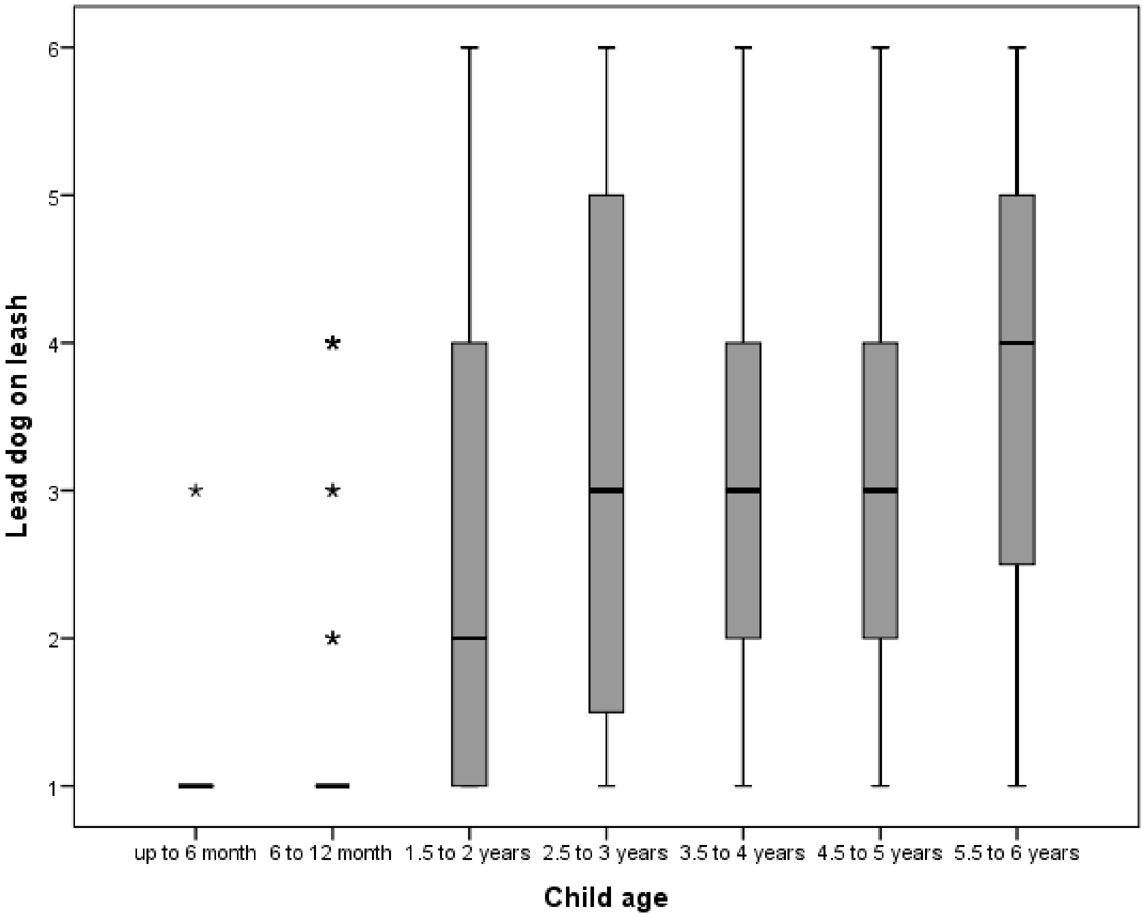

Supplementary Figure 28: Frequency of leading the dog on leash grouped by age of child (“Never” = 1 to “Very often” = 6)

6.3 Request obedience from dog/Give commands

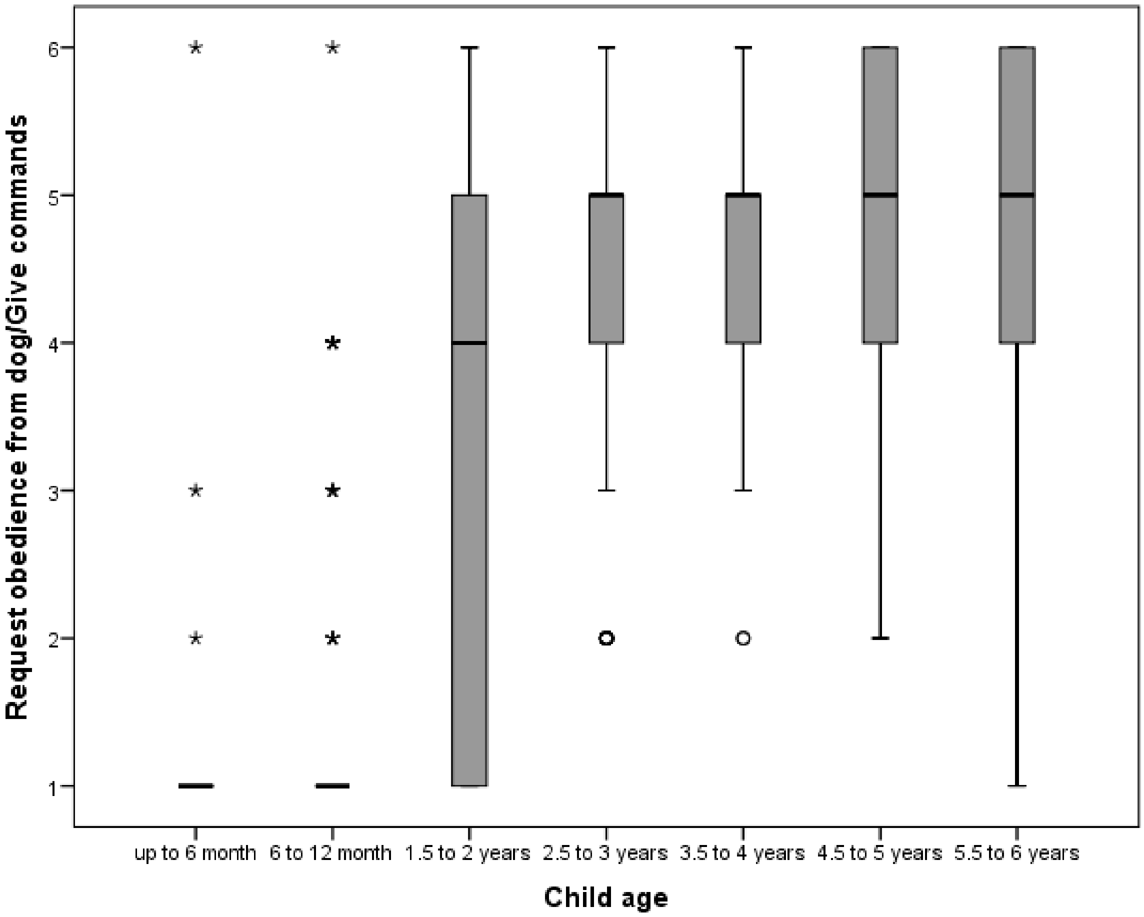

Supplementary Figure 29: Frequency of requesting obedience or giving the dog commands grouped by age of child (“Never” = 1 to “Very often” = 6)

7 Dog leaves alone/ignores child

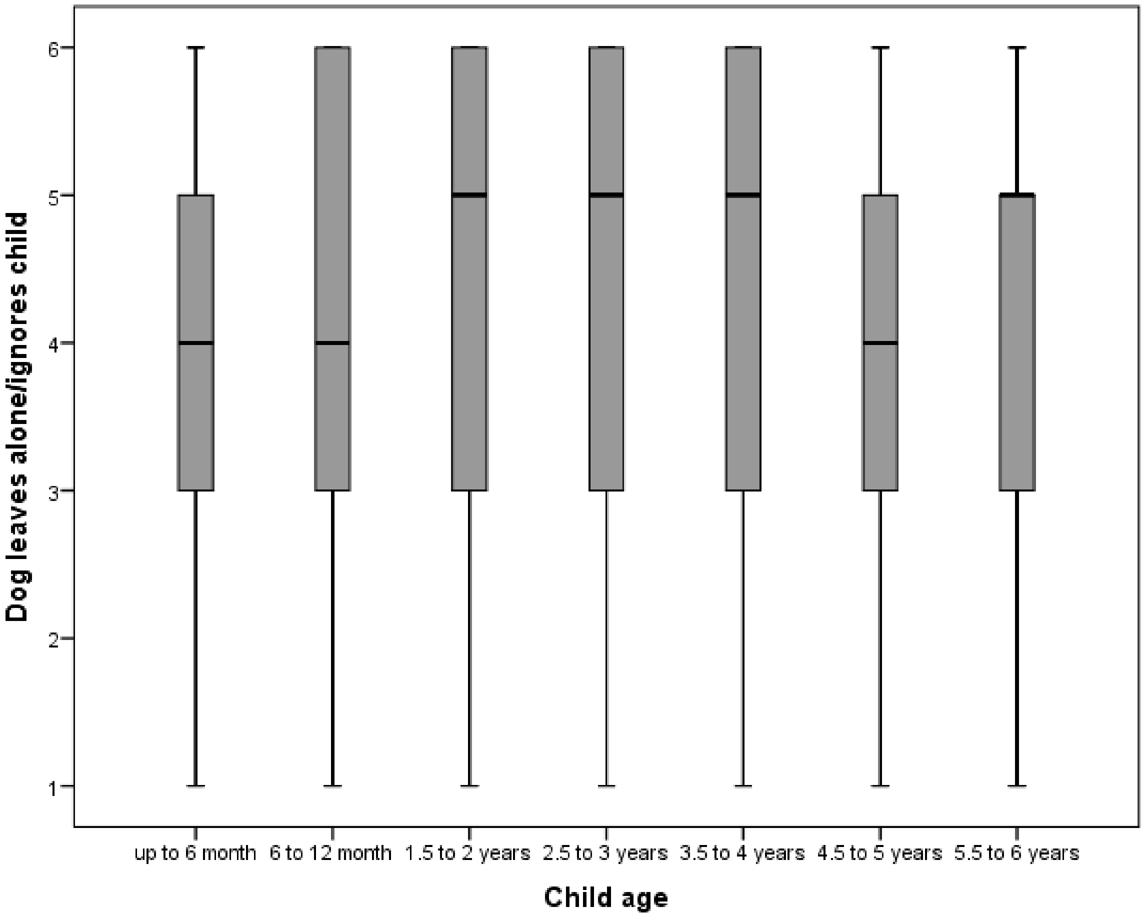

Supplementary Figure 30: Frequency of the dog leaving the child alone or ignoring the child grouped by age of child ("Never" = 1 to "Very often" = 6)

8 Dog – affiliative calm

8.1 Sniffs child

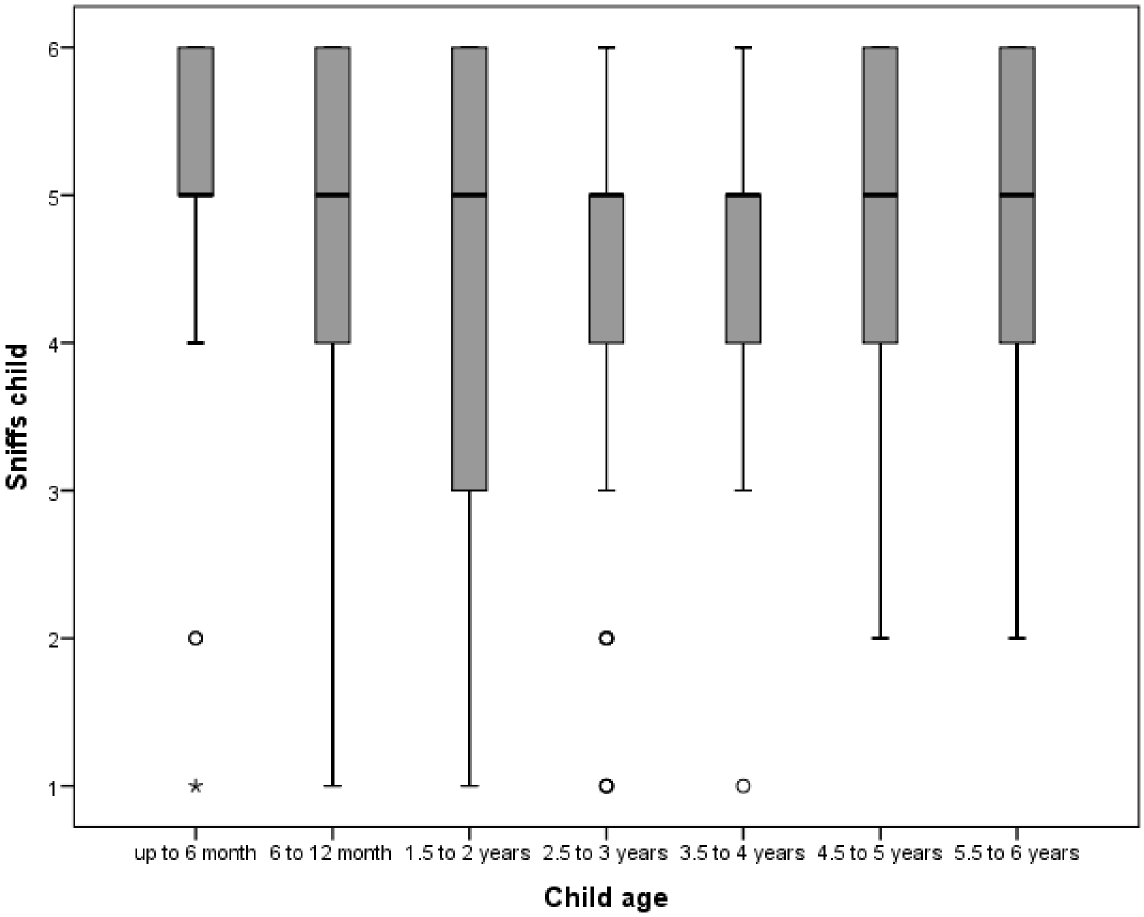

Supplementary Figure 31: Frequency of the dog sniffing the child grouped by age of child (“Never” = 1 to “Very often” = 6)

8.2 Lick hand or feet

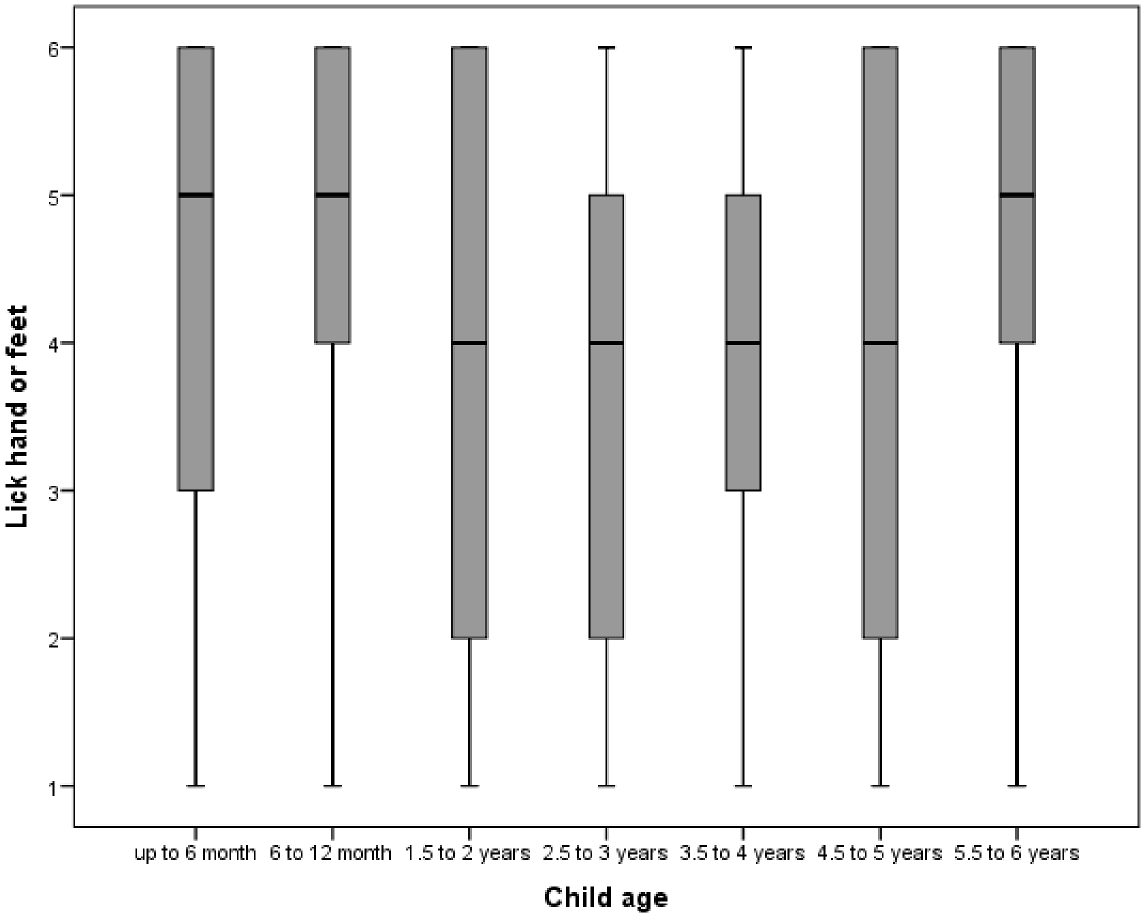

Supplementary Figure 32: Frequency of the dog licking the hands or feet of the child grouped by age of the child ("Never" = 1 to "Very often" = 6)

8.3 Lies down with body contact to child

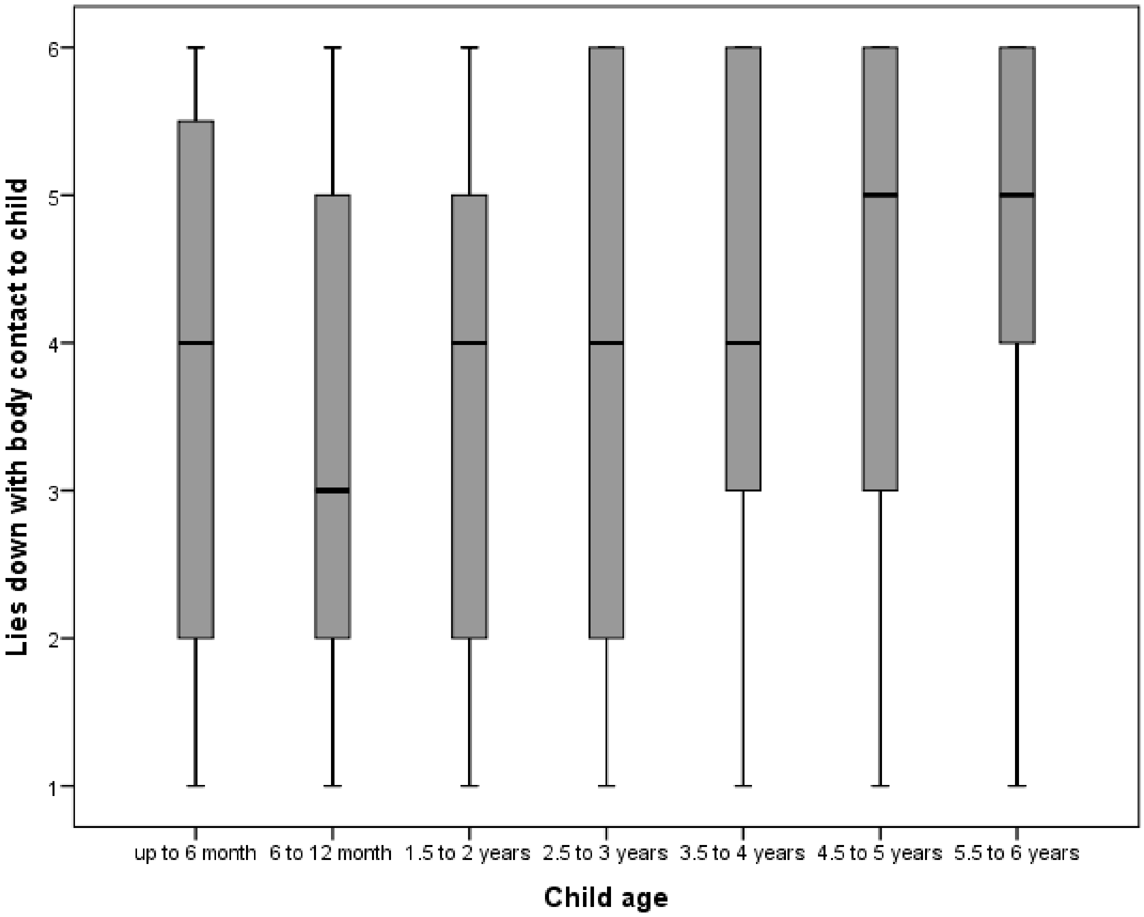

Supplementary Figure 33: Frequency of the dog lying down with body contact to the child grouped by age of the child (“Never” = 1 to “Very often” = 6)

9 Dog – affiliative energetic

9.1 Runs toward child

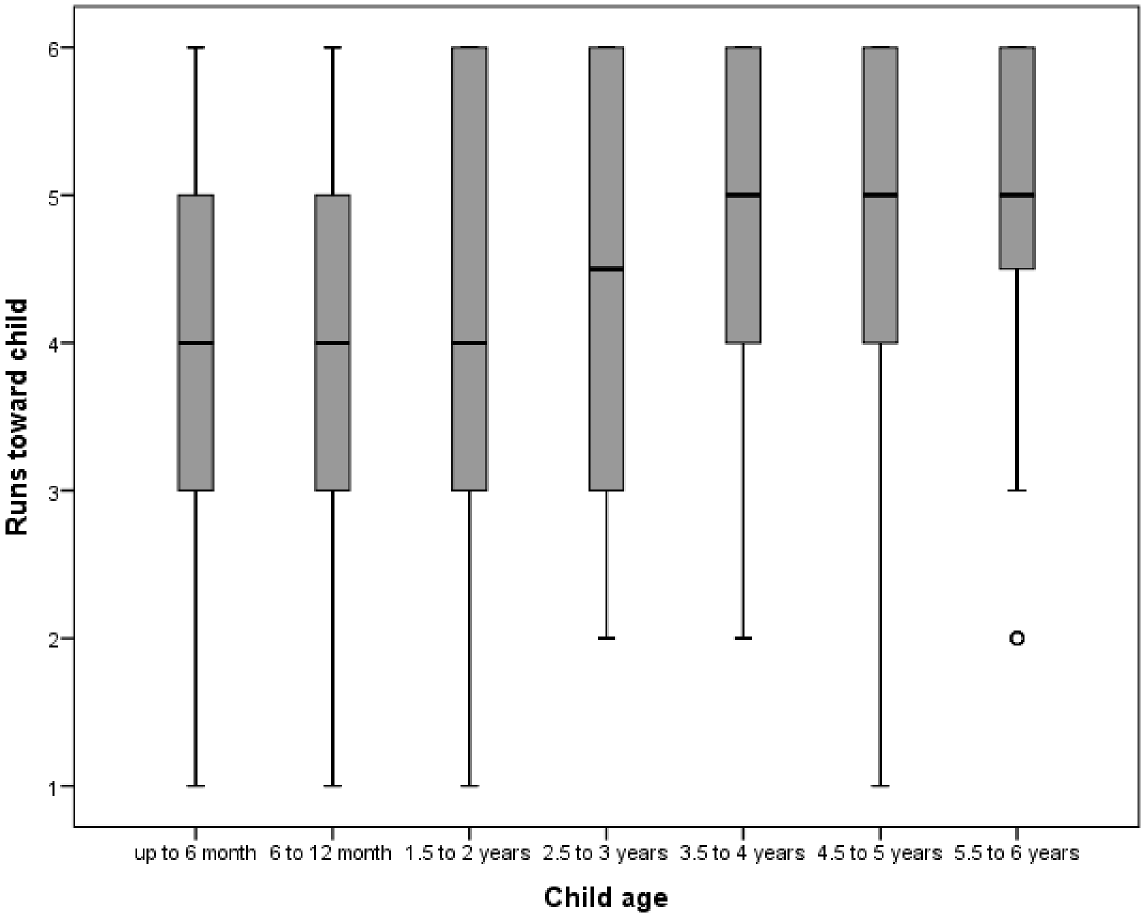

Supplementary Figure 34: Frequency of the dog running towards the child grouped by age of the child (“Never” = 1 to “Very often” = 6)

9.2 Runs after child

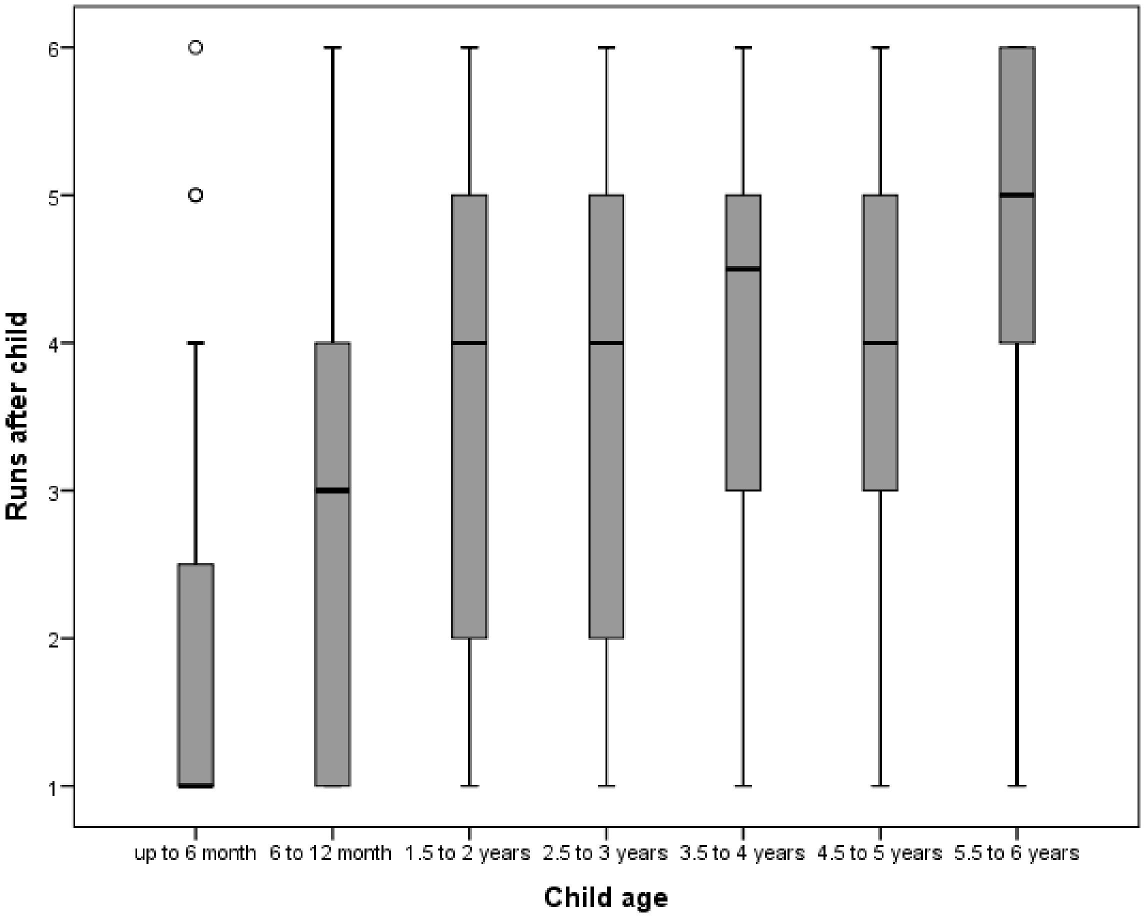

Supplementary Figure 35: Frequency of the dog running after the child grouped by age of the child ("Never" = 1 to "Very often" = 6)

9.3 Gentle mouthing

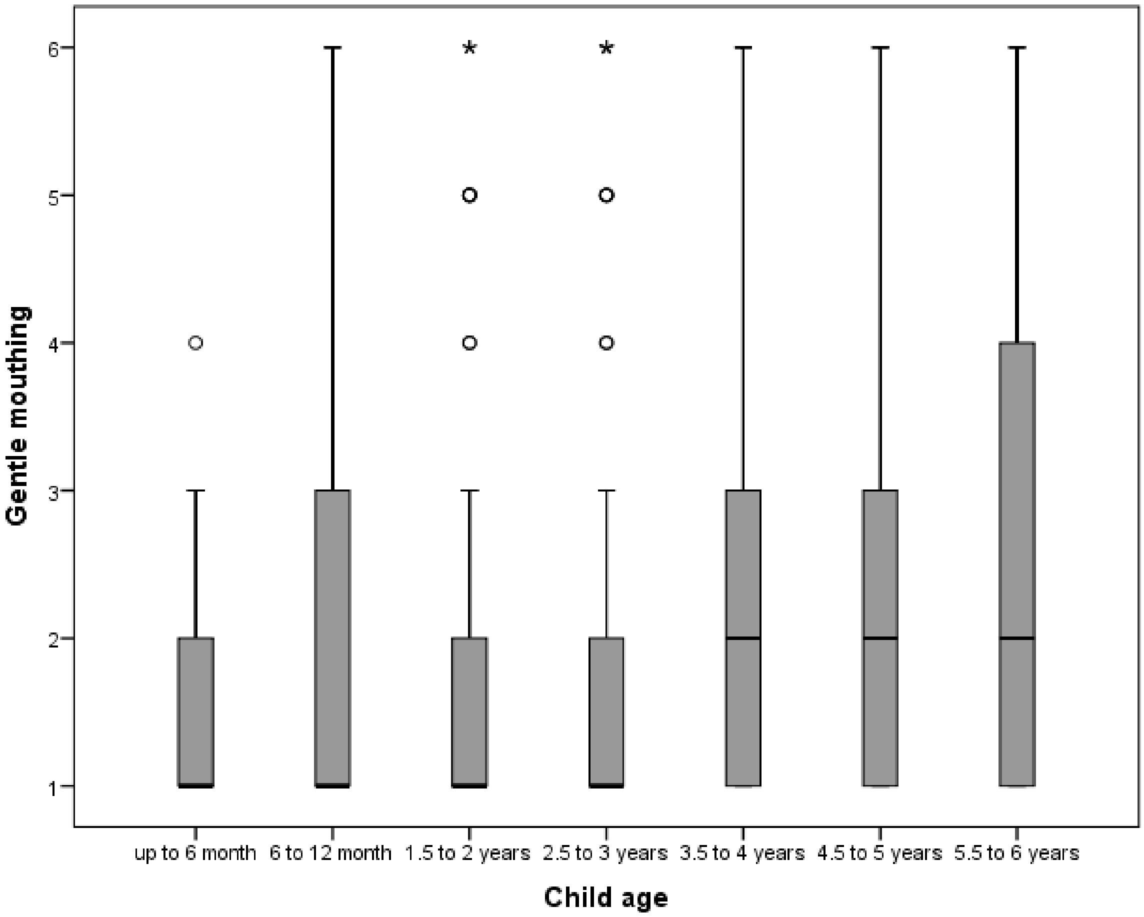

Supplementary Figure 36: Frequency of the dog gently mouthing body parts of the child grouped by age of the child (“Never” = 1 to “Very often” = 6)

9.4 Sits or lies on child

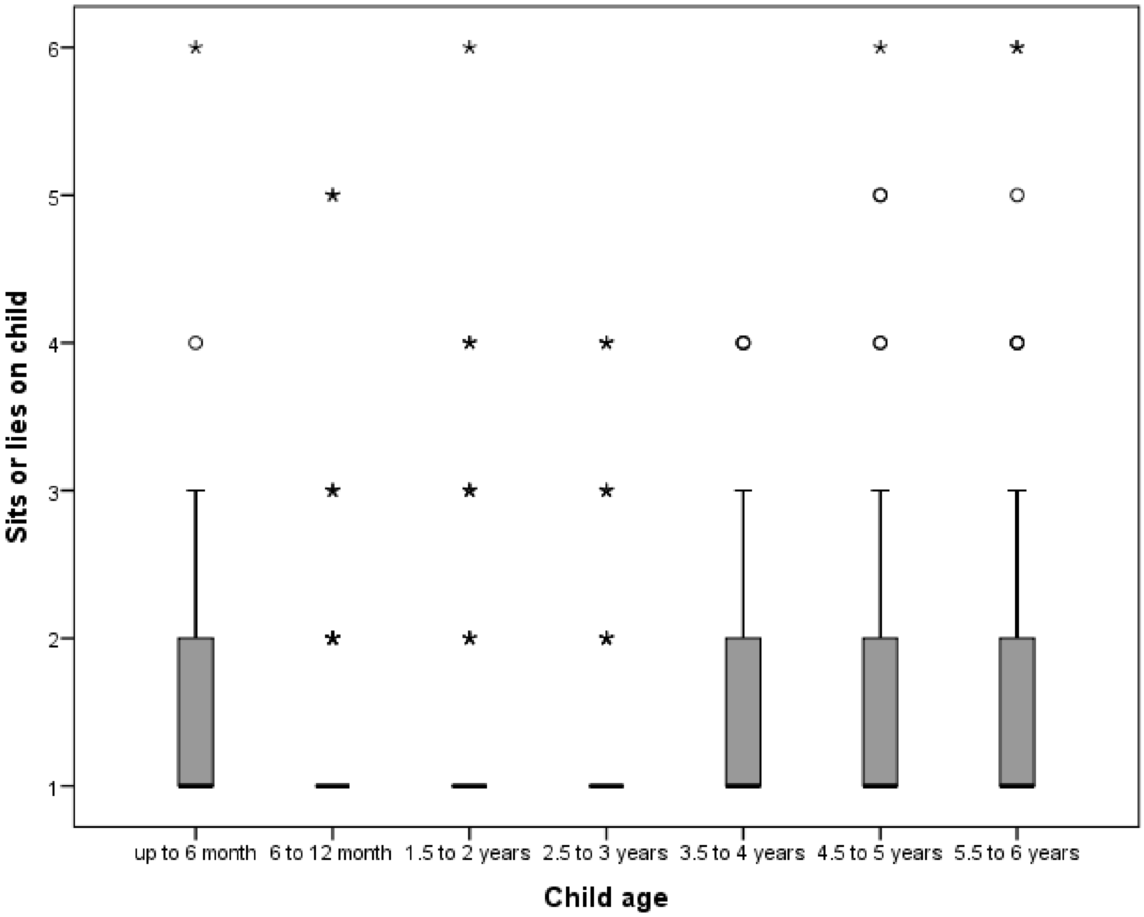

Supplementary Figure 37: Frequency of the dog sitting or lying on the child grouped by age of the child (“Never” = 1 to “Very often” = 6)

9.5 Jumps up

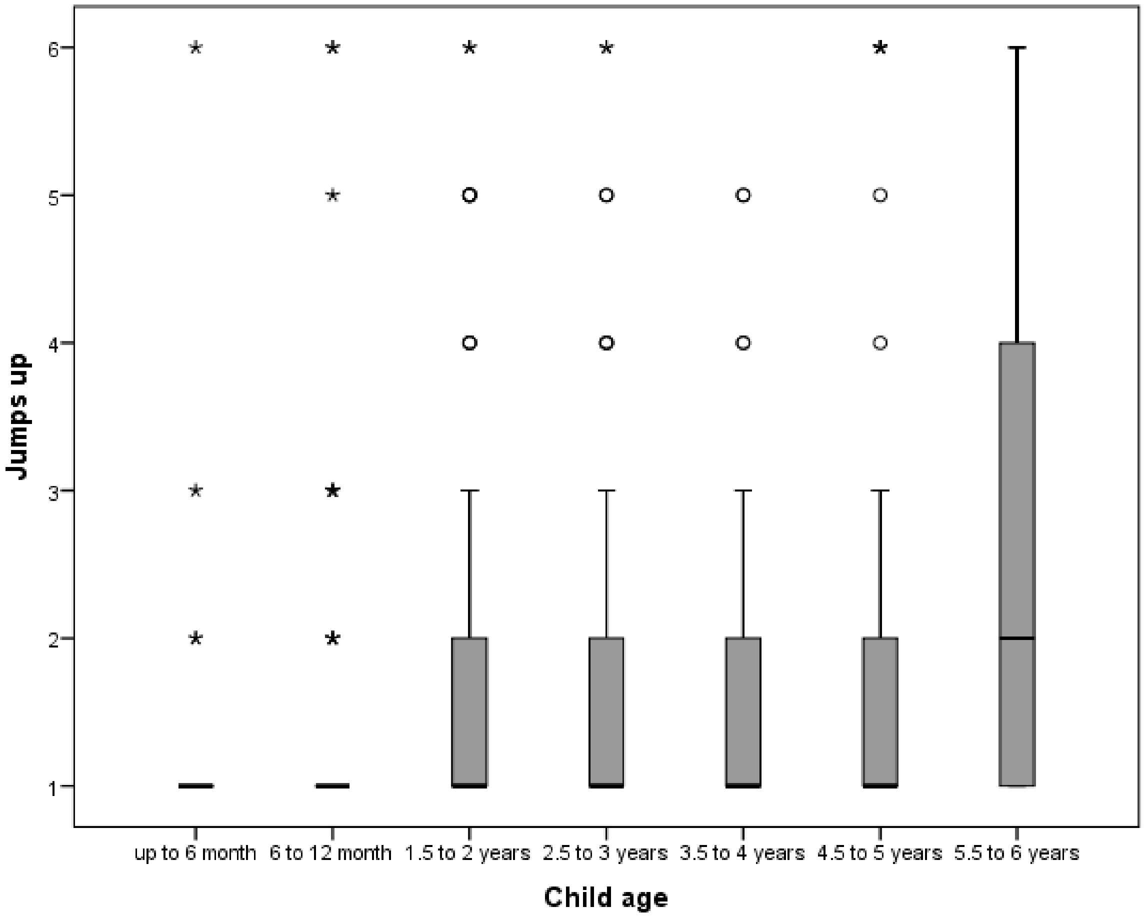

Supplementary Figure 38: Frequency of the dog jumping up on the child grouped by age of the child (“Never” = 1 to “Very often” = 6)

9.6 Knocks child over

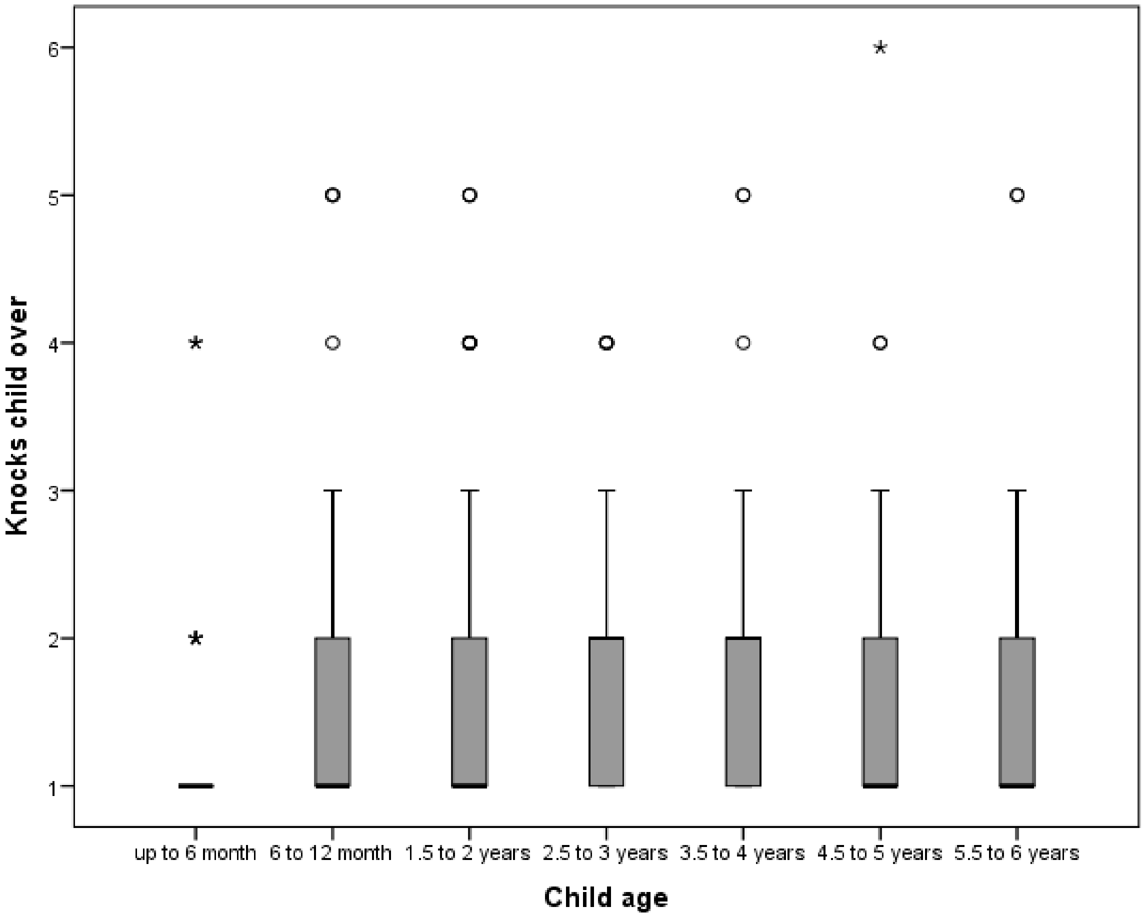

Supplementary Figure 39: Frequency of the dog knocking the child over grouped by age of the child ("Never" = 1 to "Very often" = 6)

10 Dog - resources

10.1 Takes food away from child

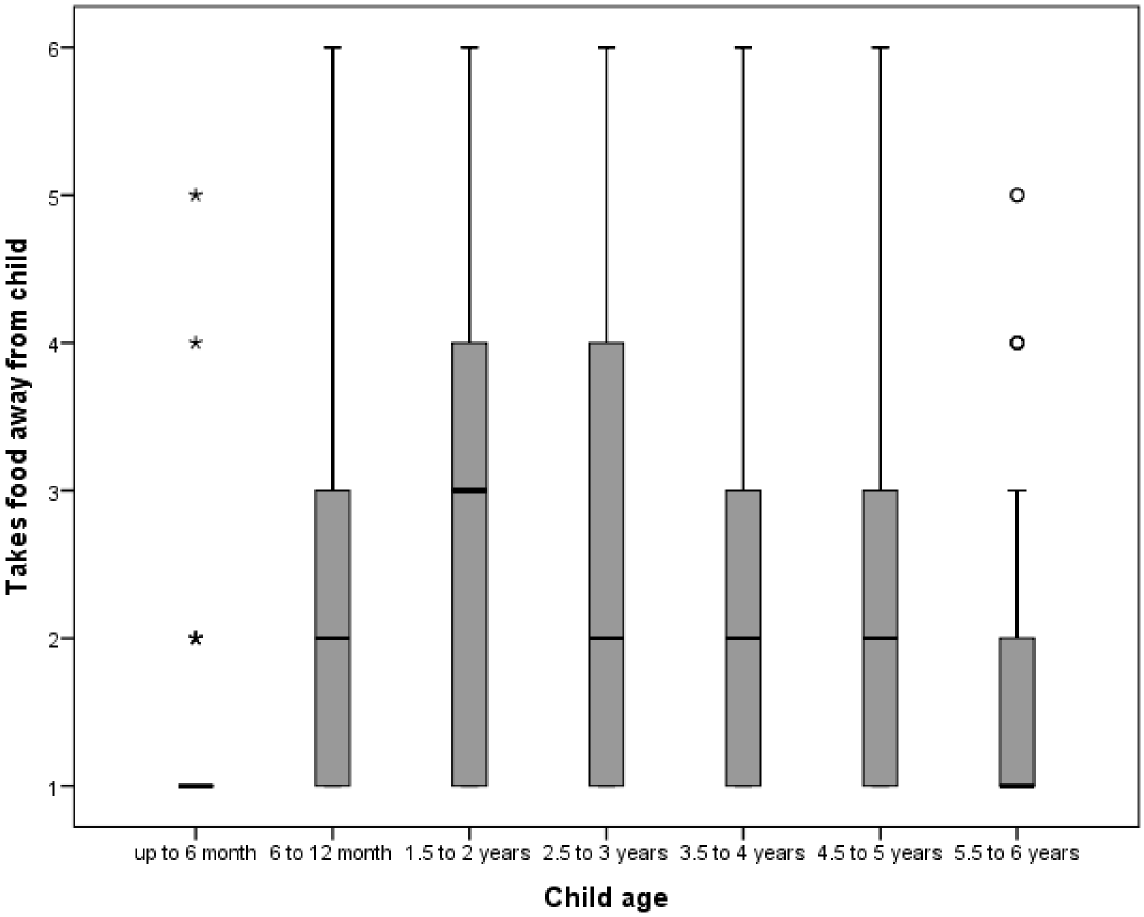

Supplementary Figure 40: Frequency of the dog taking food away from the child grouped by age of the child (“Never” = 1 to “Very often” = 6)

10.2 Takes child toys from environment

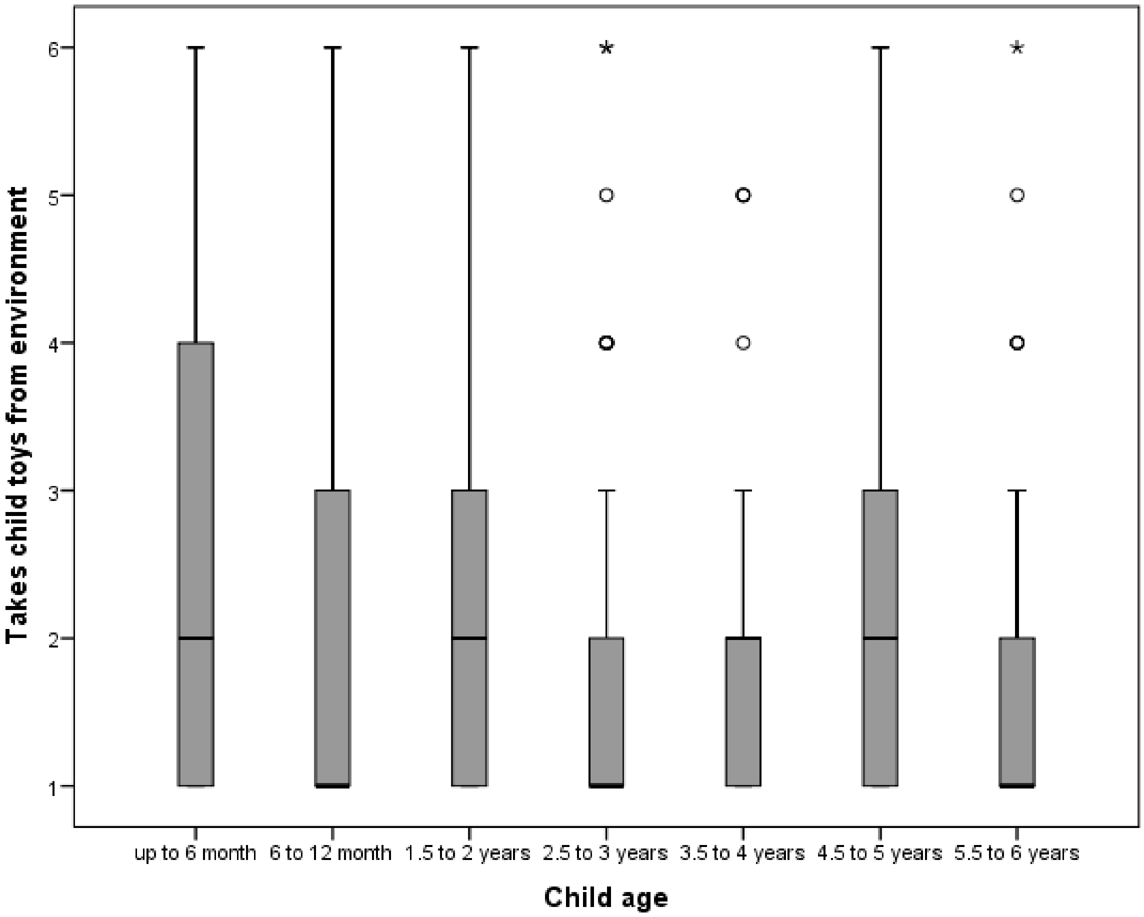

Supplementary Figure 41: Frequency of the dog taking child toys from the environment grouped by age of the child (“Never” = 1 to “Very often” = 6)

10.3 Takes child toys away from child

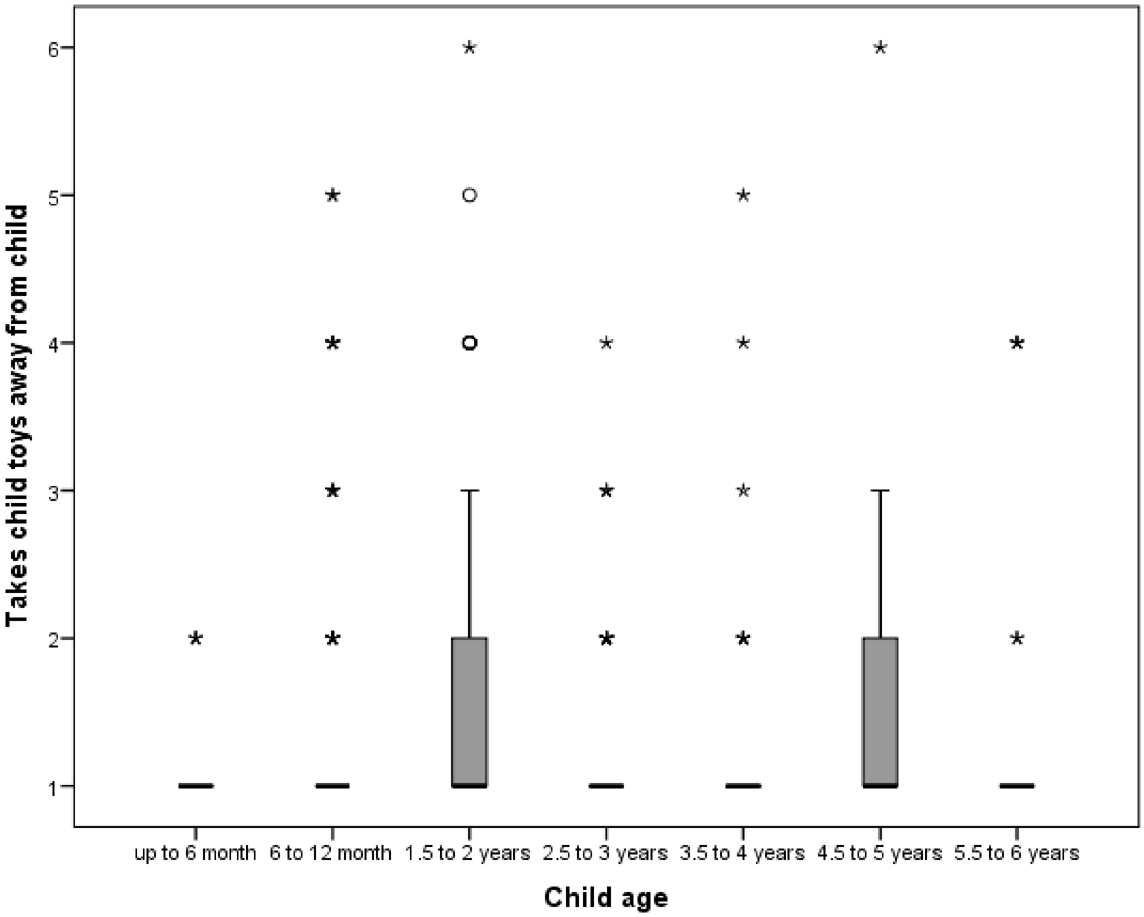

Supplementary Figure 42: Frequency of the dog taking child toys from the child grouped by age of the child (“Never” = 1 to “Very often” = 6)

10.4 Allows child to take things from dog mouth

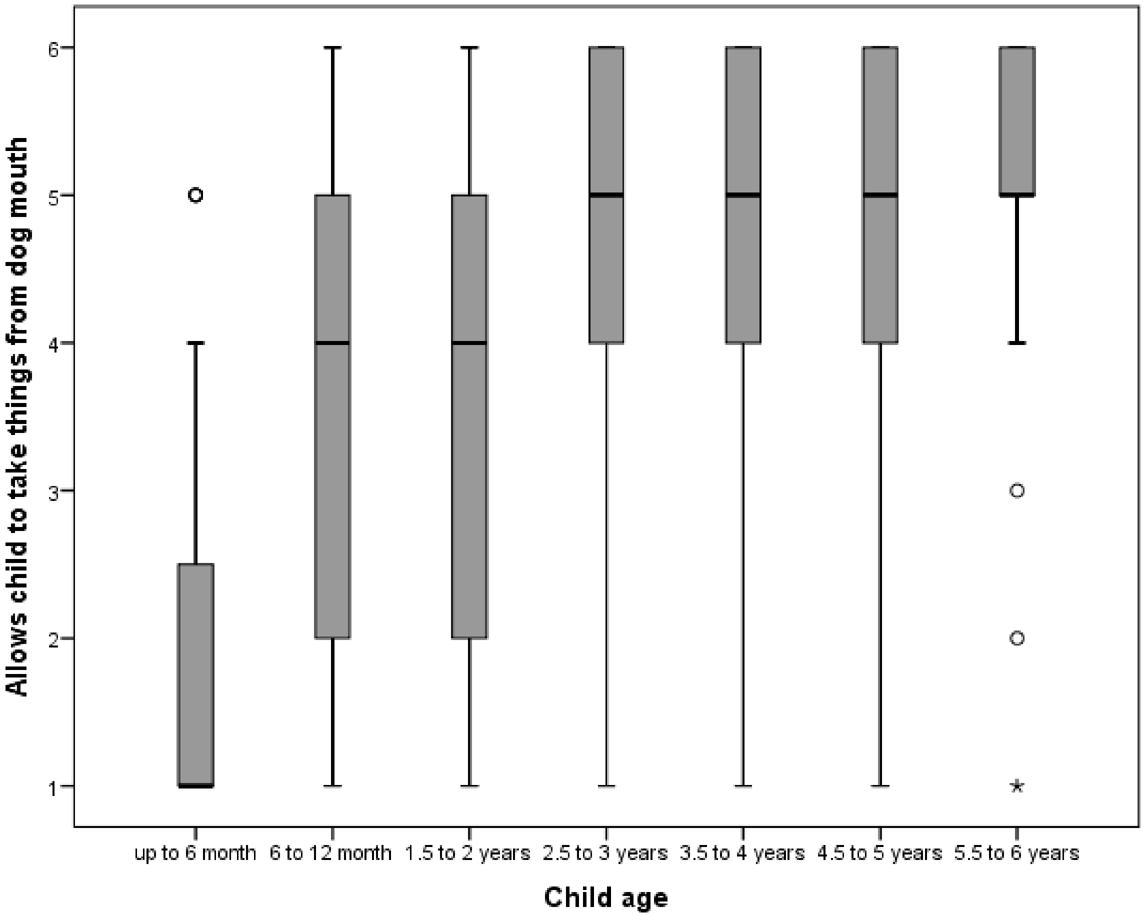

Supplementary Figure 43: Frequency of the dog allowing the child to take objects from the dogs mouth grouped by age of the child (“Never” = 1 to “Very often” = 6)

11 Dog - fear

11.1 Withdraw from child

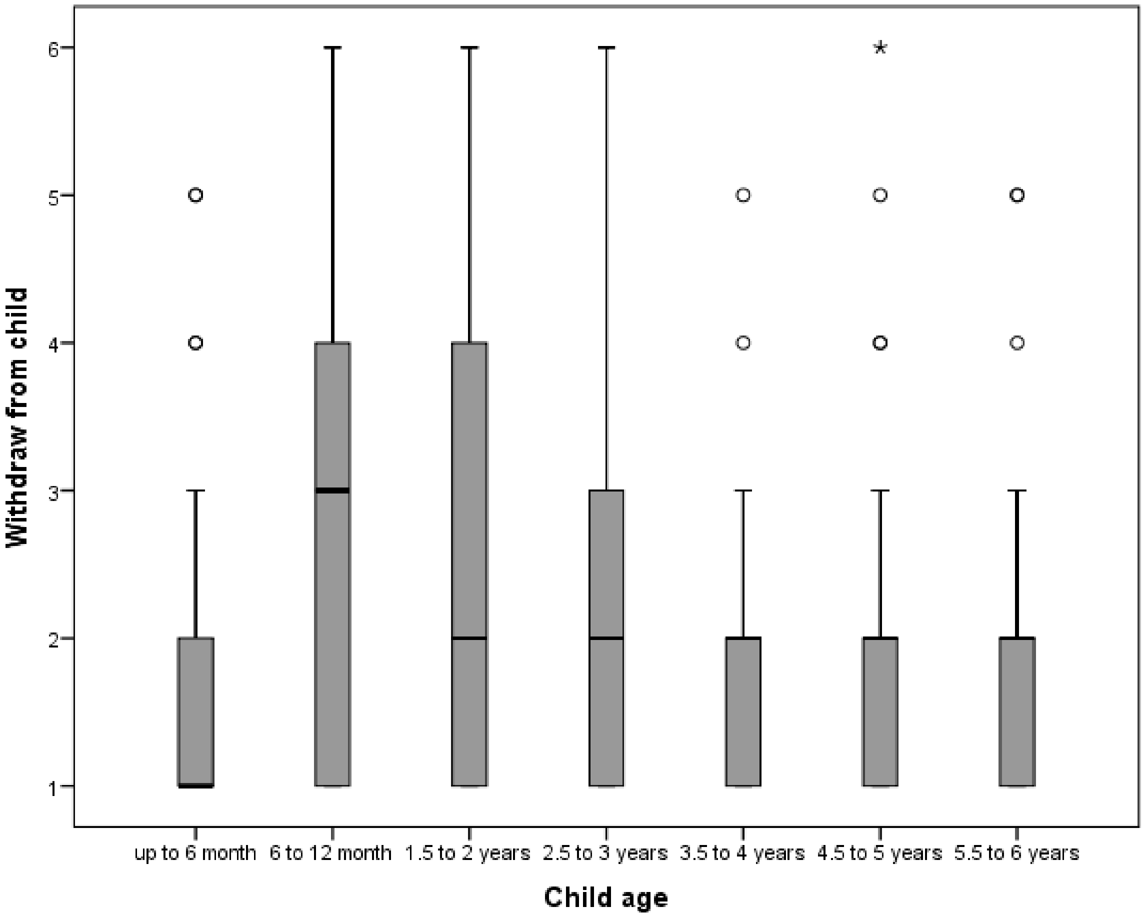

Supplementary Figure 44: Frequency of the dog withdrawing from the child grouped by age of the child (“Never” = 1 to “Very often” = 6)

11.2 Startled by child

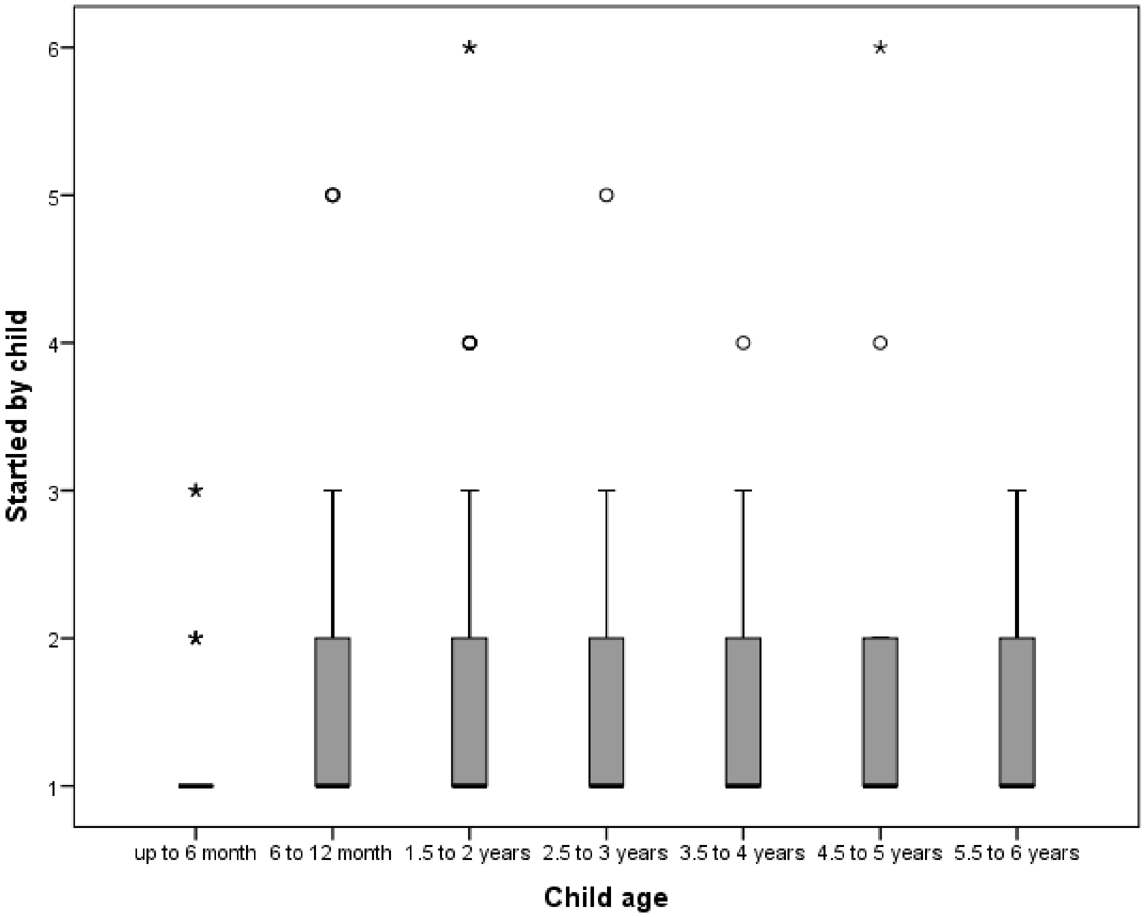

Supplementary Figure 45: Frequency of the dog being startled by the child grouped by age of the child (“Never” = 1 to “Very often” = 6)

12 Dog – aggression

12.1 Barks at child

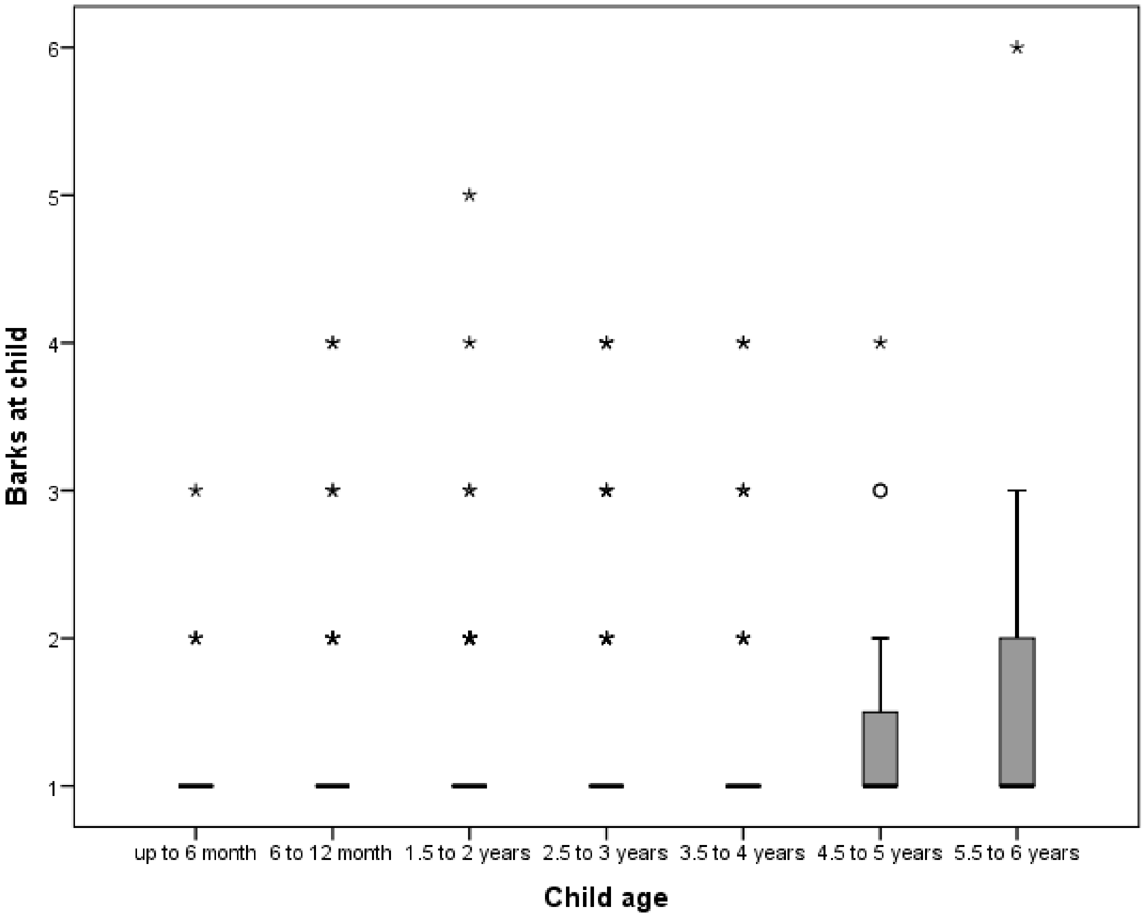

Supplementary Figure 46: Frequency of the dog barking at the child grouped by age of the child (“Never” = 1 to “Very often” = 6)

12.2 Growls during frontal approach

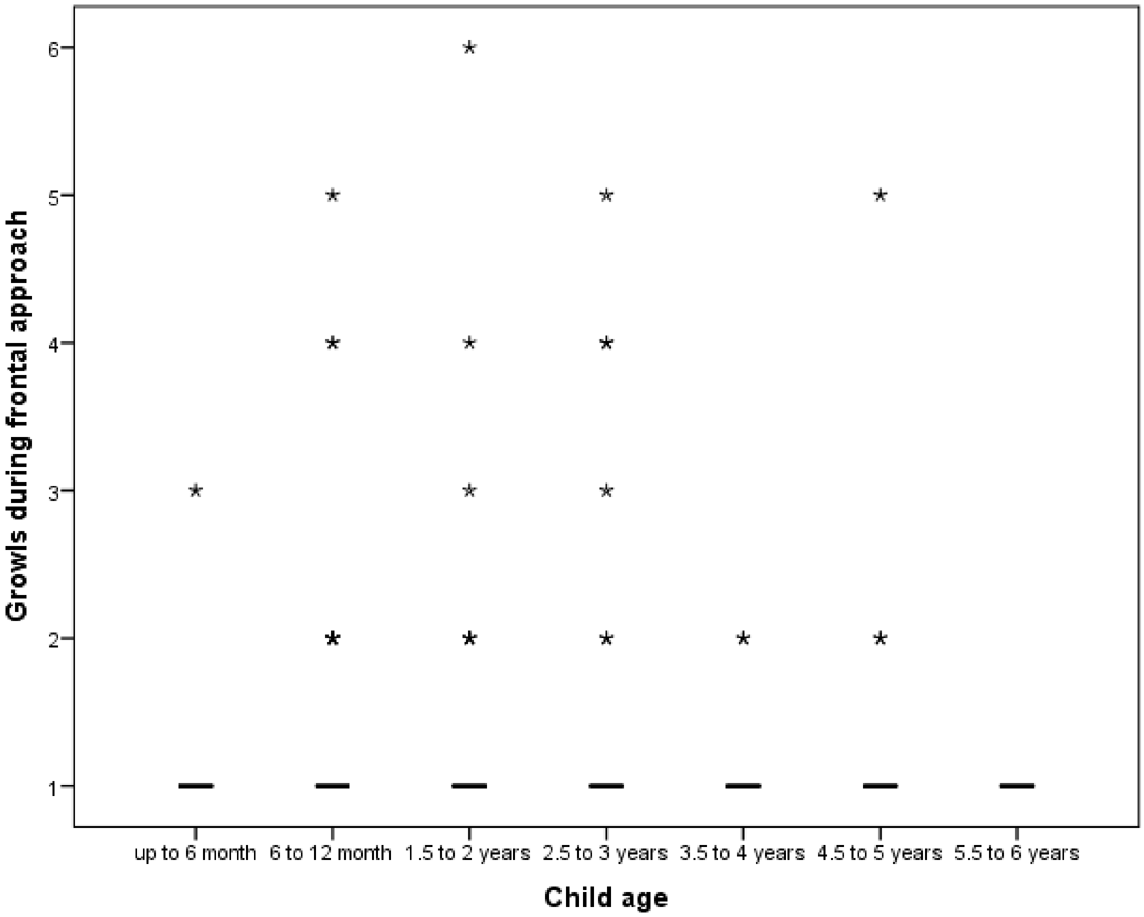

Supplementary Figure 47: Frequency of the dog growling at the child during a frontal approach grouped by age of the child (“Never” = 1 to “Very often” = 6)

12.3 Growls during passing by

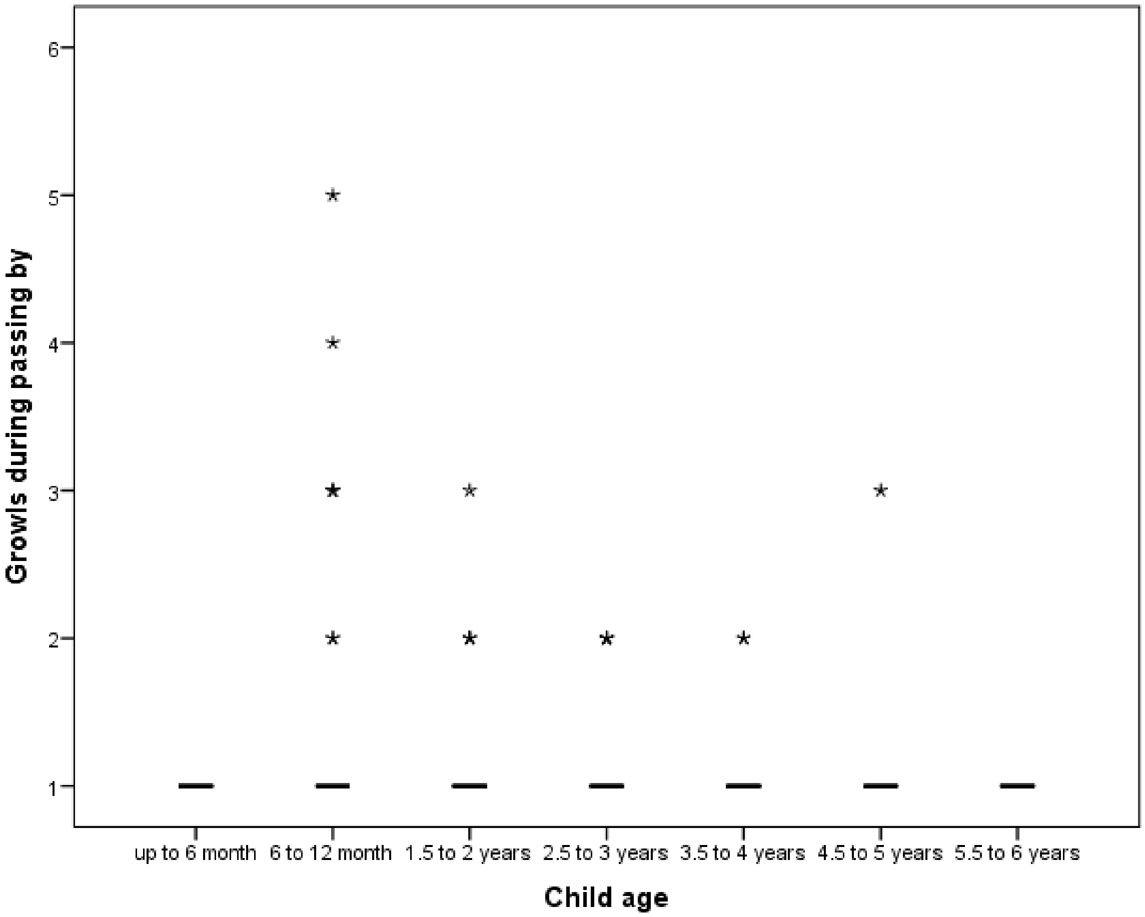

Supplementary Figure 48: Frequency of the dog growling at the child during passing by grouped by age of the child ("Never" = 1 to "Very often" = 6)

12.4 Growls with resources

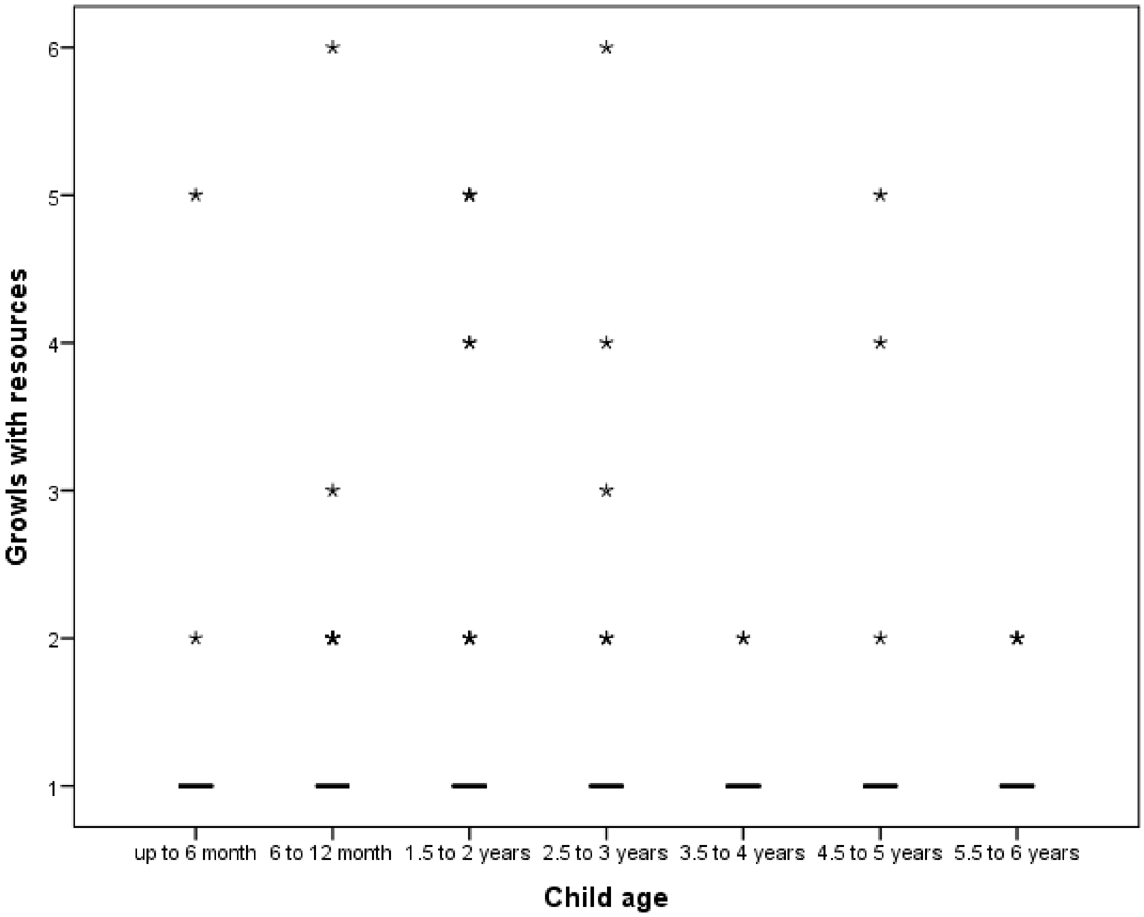

Supplementary Figure 49: Frequency of the dog growling at the child when having resources e.g. dog chew grouped by age of the child ("Never" = 1 to "Very often" = 6)

12.5 Snaps at child

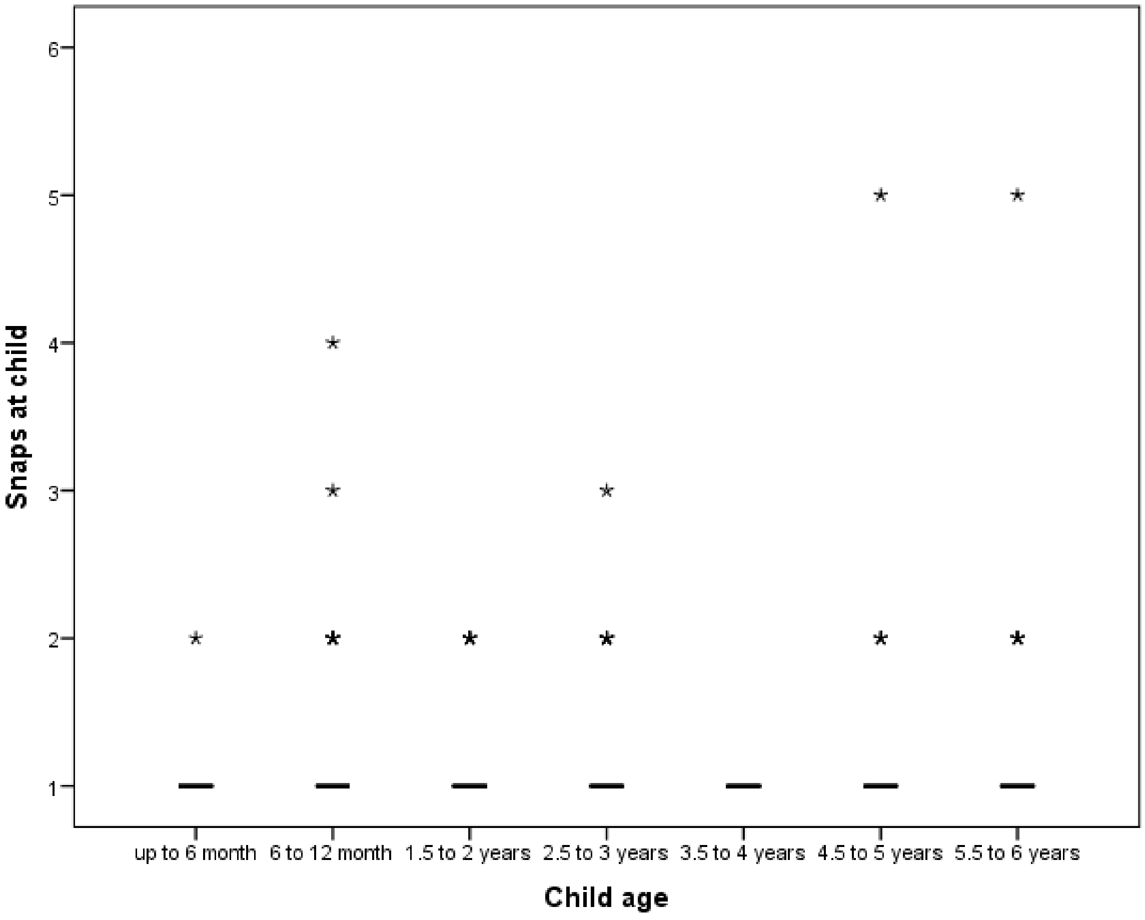

Supplementary Figure 50: Frequency of the dog snapping the child grouped by age of the child (“Never” = 1 to “Very often” = 6)
